# In-Depth Computational Analysis of Natural and Artificial Carbon Fixation Pathways

**DOI:** 10.34133/2021/9898316

**Published:** 2021-08-31

**Authors:** Hannes Löwe, Andreas Kremling

**Affiliations:** Systems Biotechnology, Technical University of Munich, Germany

## Abstract

In the recent years, engineering new-to-nature CO_2_- and C1-fixing metabolic pathways made a leap forward. New, artificial pathways promise higher yields and activity than natural ones like the Calvin-Benson-Bassham (CBB) cycle. The question remains how to best predict their *in vivo* performance and what actually makes one pathway “better” than another. In this context, we explore aerobic carbon fixation pathways by a computational approach and compare them based on their specific activity and yield on methanol, formate, and CO_2_/H_2_ considering the kinetics and thermodynamics of the reactions. Besides pathways found in nature or implemented in the laboratory, this included two completely new cycles with favorable features: the reductive citramalyl-CoA cycle and the 2-hydroxyglutarate-reverse tricarboxylic acid cycle. A comprehensive kinetic data set was collected for all enzymes of all pathways, and missing kinetic data were sampled with the Parameter Balancing algorithm. Kinetic and thermodynamic data were fed to the Enzyme Cost Minimization algorithm to check for respective inconsistencies and calculate pathway-specific activities. The specific activities of the reductive glycine pathway, the CETCH cycle, and the new reductive citramalyl-CoA cycle were predicted to match the best natural cycles with superior product-substrate yield. However, the CBB cycle performed better in terms of activity compared to the alternative pathways than previously thought. We make an argument that stoichiometric yield is likely not the most important design criterion of the CBB cycle. Still, alternative carbon fixation pathways were paretooptimal for specific activity and product-substrate yield in simulations with C1 substrates and CO_2_/H_2_ and therefore hold great potential for future applications in Industrial Biotechnology and Synthetic Biology.

## 1. Introduction

As of 2020, most of the earth’s surface is cultivated by mankind: around 50% of the habitable land is actually agricultural land with 37% of the remaining land being forests (with a decreasing trend) [1]. In order to produce more crop to feed a growing population or to fuel the bioeconomy, it is clear that either natural ecosystems have to be converted to agricultural land or agriculture has to be intensified, both with potentially tremendous downsides for nature. Crop plant areal productivity is finally limited by photosynthetic efficiency that is rather low: only around 2.4-3.7% of the incoming sunlight is converted to a usable product [2], and this is only during the growth season. The low efficiency has been attributed to the suboptimal efficiency of the reductive pentose phosphate cycle, better known as the Calvin-Benson-Bassham cycle (CBB cycle) which is the central carbon fixation pathway in plants, green algae, and cyanobacteria but also in many chemotrophic and heterotrophic microorganisms. Apart from the cycle’s high ATP demand, its key enzyme, Ribulose-1,5-Bisphosphate Carboxylase/Oxygenase (RuBisCO), is a rather slow enzyme (turnover numbers of around 1-10 s^-1^ [3]) and uses oxygen in a wasteful side reaction. The product of this reaction, phosphoglycolate, has to be recovered by the energetically wasteful photorespiratory reactions. In nature, some carboxylating enzymes can be found that have supposedly better characteristics than RuBisCO [4] which led to the question why evolution developed the dark reaction of photosynthesis the way it is. This has inspired researchers to explore whether the dark reaction of photosynthesis can be augmented to support higher productivity by mainly four mutually nonexclusive approaches: (i) enzyme engineering of RuBisCO to increase its activity [5]; (ii) implementation of CO_2_ concentration mechanisms to reduce photorespiration [6, 7]; (iii) replacing C3 with C4 photosynthesis or with completely new carbon fixation pathways, like the CETCH cycle [8]; and (iv) installing more efficient pathways for the recycling of phosphoglycolate during photorespiration [9–11]. A more detailed overview of the efforts to improve photosynthesis can be found in Bar-Even [12].

A further option to circumvent the limitations of the areal productivity of photosynthesis is to use alternative sources of energy and carbon, like the C1 compounds formic acid and methanol or a mixture of H_2_ and CO_2_ gas. These electron and carbon sources can be derived from electrolysis of water or from CO_2_ which increases the areal productivity compared to crop-based primary production by an order of magnitude [13]. Alternatively, methanol or syngas can be produced from waste or biomass which are abundantly available and do not need additional area. For the assimilation of these compounds, nature also has evolved specialized microorganisms with dedicated pathways like the Serine cycle or the Ribulose Monophosphate (RuMP) cycle that are intrinsically more electron-efficient than the traditional CBB cycle [14]. For biotechnological purposes, it was tried to implant these pathways into *Escherichia coli* with mixed success in the beginning ([15]; H. [16, 17]), but finally, the RuMP cycle could be implemented in *E. coli* to support growth on methanol as the sole carbon and energy source (F. Y. H. [18]). Additionally, multiple optimized pathways for C1-assimilation have been proposed for bioproduction, and their basic working principle could be shown [19–21]. Among them, the reductive glycine pathway (rGlyP) was first proposed as an alternative pathway used for CO_2_ fixation in acetogens [22] and later engineered in aerobic hosts by the group of Arren Bar-Even and others. This pathway shines as it is the potentially most efficient aerobic formate-dependent pathway and one of the few pathways to be integrated *in vivo* to support growth with formate as the sole carbon and energy source [23–25]. To acknowledge this important achievement, this work is dedicated to Arren Bar-Even, a visionary in pathway design and metabolic engineering, who passed away far too early in the zenith of his scientific achievements.

Throughout this article, we will call biochemical pathways simply “pathways,” which may be linear biochemical pathways or cycles, for simplification. When comparing different pathways, metabolic routes were usually thought to be better because of the following reasons: (i) a lower demand of ATP to form a product [4, 8, 20], (ii) key enzymes with a higher activity ([26], and [4, 12]), (iii) a higher mean thermodynamic driving force (i.e., free energy ΔG) of the reactions [21, 27, 28], or (iv) a higher affinity for CO_2_ or HCO_3_^-^ of the carboxylating enzymes ([26], and [4, 12]). All these criteria have limitations, however, and there hasn’t been a conclusive comparison to our knowledge that systematically took into account the enzyme kinetics apart from the forward rate constant. As more efficient pathways are supposed to have lower energy losses and thus usually operate closer to thermodynamic equilibrium, reversibility of reactions accounts for a higher demand for the respective enzymes. Additionally, kinetic undersaturation will limit a pathway’s activity with RuBisCO’s affinity for CO_2_ being a prominent example. Noor et al. developed a method to integrate these additional thermodynamic and kinetic constraints by means of the Enzyme Cost Minimization (ECM) algorithm [29]. This algorithm predicts optimal pathway activities if thermodynamic and kinetic data are available. Missing kinetic parameters can be estimated using the Parameter Balancing algorithm [30] to yield a complete and consistent set of parameters for each reaction.

In this work, we made an attempt to objectively compare natural occurring and artificial pathways considering their specific activity and the predicted yield on different industrially relevant electron and carbon sources with a computational approach. Only oxygen-tolerant pathways were considered since data for most of the anaerobic pathways are scarce and ATP-intensive metabolites cannot be produced without by-product formation [31, 32]. By recombining reactions of existing pathways and by adding reactions that were introduced in recent publications, we present 2 new artificial pathways: the reductive citramalyl-CoA cycle (rCCC) and the 2-hydroxyglutarate-reductive tricarboxylic acid cycle (2-HG-rTCA cycle). Both were designed taking into account common thermodynamic and kinetic bottlenecks of other pathways, resulting in a good trade-off between activity and product-substrate yield. Finally, we compare natural and artificial pathways using the ECM algorithm and draw conclusions about evolution, design principles, and future perspectives of these pathways.

## 2. Material and Methods

### 2.1. Pathway Selection and Construction of New Routes

The metabolic pathways that were explored in this study were chosen based on the following criteria:

(i) The metabolic routes needed to include a reaction that uses CO_2_ or a C1 compound (formate, methanol) as a substrate
(ii) The enzymes constituting the pathways are active under aerobic conditions
(iii) Sufficient kinetic data were available from the literature
(iv) Additionally, more common routes that connect isolated pathways were added, like glycolysis or the TCA cycle

To design modified versions or combinations of pathways, we followed an intuitive, manual approach in contrast to the systematic methodology suggested in former studies [4, 33] as we sought new routes aside of the previously proposed ones. This was achieved by providing new reaction stoichiometries, oftentimes side reactions of known enzymes, but also by the theoretical dissection of pathways into functional modules that could be freely combined.

### 2.2. Data Collection

**Figure 1:**
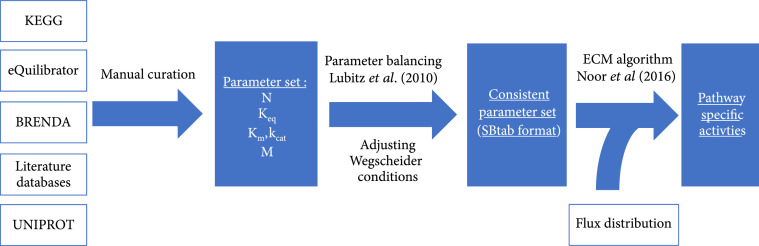
Schematic workflow that was applied to calculate the pathway-specific activities from the kinetic parameter set and the flux distributions of each pathway. Box with white-colored filling indicate input data(sources), blue boxes indicate processed data. N: stoichiometric matrix; $K_{\text{eq}}$: equilibrium constants; $K_{m}$: Michaelis constants; $k_{\text{cat}}$: forward rate constants; M: molecular weight.

In order to quantify the pathway-specific activities of CO_2_- and C1-fixing pathways, a comprehensive data set was collected from different sources which was then used for further calculations. Thermodynamic and enzymatic data were extracted from the databases KEGG [34], eQuilibrator [35], BRENDA [36] and UniProt [37]. The details are listed in Supplementary Table S1 specifying which kinetic data were extracted from each source. Some equilibrium constants of reactions that were critical for certain carbon fixation pathways were adjusted based on enzymatic data as described in Section 3 in the Supplementary Information. For most enzymes, kinetic data exists for enzymes from various organisms under varying conditions. We chose parameters of optimal enzymes (i.e., those that allow for the highest activity) to explore the potential of each pathway. These enzymes could be derived from any organisms in all domains of life. All primary sources were manually curated from original publications since parameters in BRENDA are often stored with wrong units or are only partially extracted. The complete workflow is illustrated in Figure 1. During data collection, kinetic parameter sets with more data for Michaelis constants and those with forward and backward rate constants were preferred over sets with less measured data. The most important parameters which are decisive for a good estimate are the rate constant of the direction the reaction is supposed to operate and the $K_{m}$ of the primary substrate which is usually the carbon intermediate in the respective pathway.

All kinetic data were brought together in an SBtab model file and SBtab data file as specified in [29, 38]. The templates of the respective SBtab files were provided by the ECM pathway analysis platform on the eQuilibrator website (http://equilibrator.weizmann.ac.il/pathway/). An additional column for parameter uncertainties was added, and table headings were slightly modified to match the current implementation of the Metabolic Network Toolbox (https://github.com/liebermeister/metabolic-network-toolbox) in MATLAB that was used to process the data. The final SBtab files are included in the Supplementary Information (available here) and also at https://github.com/hannesloewe/pathway-comparison-ECM.

### 2.3. Integration of Quinone-Dependent and Proton-Translocating Reactions

During data collection, it had to be decided which enzymes are used for each reaction as there are cases in which a reaction with identical main substrate and product is catalyzed by different enzymes. The only difference in these cases might be the use of different cofactors or whether the reaction is coupled to ion translocation through the membrane. This was especially important for the electron transport chain. It is known that depending on the organism and the conditions, different variants of the electron transport chain occur [39]. Additionally, different quinones with varying redox potentials might be used. For the analysis of pathways, quinones were treated as cofactors like other electron carriers, e.g., NADH. In the Tricitric Acid cycle (TCA cycle), the direction of the succinate dehydrogenase complex is towards the oxidation of succinate which is usually accompanied by ubiquinone reduction due to the high redox potential of the ubiquinone (UQ)/ubiquinol (UQH2) redox couple [40]. To poise the reaction in the reverse, reductive direction, bacteria, e.g., *E. coli* [40], usually use quinones with a lower redox potential like the menaquinone couple. Irrespective, whether ubiquinone, menaquinone, or even rhodoquinone is used, the oxidation of succinate was reported to be reversible [41–43]. The direction of the reaction is rather determined by the redox potential and ratio of the reduced and oxidized form of the quinone. The use of a soluble NADH-dependent fumarate reductase that is operating in the rTCA cycle of certain bacteria [44] was suggested for the implementation of new pathways by others as an alternative with a higher driving force due to the higher redox potential of NADH compared to quinones [4]. This enzyme was not considered here since the reaction is practically irreversible and hence energetically more wasteful. Instead, we used the data of the succinate dehydrogenase of *Bacillus subtilis* that uses menaquinone as it reversibly works under aerobic conditions and kinetic data were available for forward and backward reaction [42]. In *B. subtilis*, the oxidation of succinate is accompanied by the translocation of 2 protons from the periplasm to the cytosol. To account for the 2 protons that are pumped by the enzyme through the membrane, the equilibrium constant of this reaction was adjusted since the proton gradient was not included in the equilibrium constant taken from eQuilibrator. The change in the equilibrium constant was calculated according to the following formula, assuming a membrane potential ∆ψ of -150 mV:

(1)Keq′=Keq0 ezF∆ψ/RT,

in which $K_{\text{eq}}^{\prime }$ is the adjusted equilibrium constant, $K_{\text{eq}}^{0}$ is the original equilibrium constant provided by eQuilibrator, F is the Faraday constant, z is the number of protons transferred from the cytosol to the periplasm, ∆ψ is the transmembrane potential, R is the general gas constant, and T is the temperature.

Depending on the direction of the proton translocation, the exponent of the exponential function in Equation (1) will have a positive or negative exponent (per definition, z is positive for transport from the cytosol to the periplasm). When the reaction is using succinate as the substrate and fumarate as the product, the equilibrium constant has to be higher than without considering the proton gradient (2 protons from the periplasm are transferred to the cytoplasm), and hence, the exponent is positive. Apart from the succinate dehydrogenase, only one other type reaction with quinones was implemented: acyl-CoA dehydrogenases that desaturate carbon-carbon single bonds, as found, e.g., in *β*-oxidation. These enzymes were assumed to channel the electrons to the electron transport chain at the level of ubiquinone which is an exergonic reaction under physiological conditions.

### 2.4. Kinetic and Thermodynamic Parameter Estimation by Parameter Balancing

The SBtab files with the collected data were used as the input for the Parameter Balancing algorithm [30] to estimate the missing kinetic parameters following essentially the approach used in a former study [29]. The theoretical background concerning this algorithm is explained in detail in the supplementary information in Section 4 and in the original publication. Each reaction was balanced alone without considering the other reactions in the network using the MATLAB implementation of Parameter Balancing. The equilibrium constants were not adjusted but kept fixed during balancing to ensure thermodynamic consistency across reaction networks. This was done by setting the option “fix_Keq_in_sampling” to 1 in the MATLAB code. After Parameter Balancing, the resulting parameters should fulfil the Haldane relationship which can be formulated as follows [29]:

(2)Keq=kcat+∏iKiPmiPkcat−∏iKiSmiS,

in which $K_{\text{eq}}$ is the equilibrium constant, $k_{\text{cat}}$ is the forward and backward rate constants, $K_{i}^{P}$ is the Michaelis constant of product i, $m_{i}^{P}$ is the stoichiometric factor of product i, $K_{i}^{S}$ is the Michaelis constant of substrate i, and $m_{i}^{S}$ is the stoichiometric factor of substrate i.

For some reactions, the algorithm did not produce consistent sets of parameters because the collected parameter values were contradictory with respect to the equilibrium constants of the reactions. This would typically happen if forward and backward kinetic constants were both available. In these cases, the uncertainties of the measured constants as well as the parameter prior uncertainties of kinetic rate constants and Michaelis constants were iteratively increased and balanced until the Haldane relationships were fulfilled. The Parameter Balancing algorithm did not give estimates only for parameter values but also for their uncertainties which were used later to estimate the error of optimization results. Both parameter values and their uncertainties were stored in data structures for further calculations. After this step, the data structure with the network kinetics contained a complete set of parameters for all reactions and all reactants. The corresponding MATLAB code can be found at https://github.com/hannesloewe/pathway-comparison-ECM.

### 2.5. Determination of Pathway-Specific Activities

To calculate the pathway-specific activity of a selected pathway, the MATLAB implementation of the ECM algorithm was used [29]. The MATLAB function for ECM needs four arguments: (i) a biochemical network containing the reaction stoichiometries, (ii) the kinetic parameters of the reactions, (iii) a flux distribution representing the selected pathway, and (iv) an option data structure which can be used to specify upper and lower bounds for metabolites and cost weights for enzyme concentrations as well as further options. These four arguments were provided as follows: The reaction stoichiometries (i) were specified in the SBtab model file that was assembled during data collection. The stoichiometries were loaded into a data structure with the Metabolic Network Toolbox. The data structure with the kinetic parameters (ii) were obtained by Parameter Balancing (for details, compare the respective section in the Material and Methods). For each pathway, a flux distribution (iii) was set up as a vector that reflects fluxes in the selected pathway. For the upper and lower bounds of metabolite concentrations (iv), the standard bounds provided by the ECM pathway analysis platform of eQuilibrator were taken and slightly modified with values from the literature to represent a realistic profile of metabolite concentrations (detailed values for metabolite bounds can be found in the Supplementary Information in Table S3). Depending on the analysis, CO_2_ was either assumed to equal 10 *μ*M which is in the order of air-saturated water or 1 mM to account for scenarios where CO_2_ can be actively fed (e.g., in bioreactors). HCO_3_^-^ which is used by many carboxylating enzymes instead of CO_2_ was assumed to be approximately in equilibrium with CO_2_ at a pH of 7.5. As enzyme cost weights (iv), the molecular weights of the enzymes were taken as it was assumed that bigger enzymes will be costlier to synthetize for the cells [29, 45]. The molecular weights of enzymes are stored in a table in the SBtab model file and were loaded into a data structure in MATLAB.

The ECM algorithm returns the minimal enzyme concentrations to support the given flux distribution and thereby offers a measure of the optimal pathway activity per milligram of total enzyme. In addition, the enzyme capacities, reversibility, and kinetic factors [29] (also compare Supplementary Equation S2) are returned by the algorithm. With these factors, it can be evaluated if a certain reaction is thermodynamically or kinetically limited as described in the section on Parameter Balancing. Uncertainties of pathway-specific activities were estimated by a Monte Carlo method, where in every iteration, the input parameters (i.e., the kinetic parameters) were randomly varied according to their geometric standard deviations assuming a log-normal distribution. The final values that are presented in Results are the geometric mean values and the geometric standard deviations of 100 iterations.

### 2.6. Calculation of Projected ATP Costs and Product-to-Substrate Yields of Pathways

To compare the pathways in terms of efficiency, a common criterion for all pathways had to be found as they use different cofactors. This was done by calculating the maximal predicted yield of a desired product from a given substrate. To cover the ATP demand of pathways, it was assumed that it is regenerated by the respiratory chain from NADH to yield a total of 2.5 ATP during optimal aerobic respiration [46] which represents an optimistic estimate. In reactions that produce AMP, it was assumed that two equivalents of ATP are required for its regeneration to ATP. FADH2 which is a cofactor of the succinate dehydrogenase complex is supposed to yield 1.5 ATP when it is fully oxidized with O_2_ [46]; the same should be true for electrons derived from the oxidation of FADH2-dependent acyl-CoA dehydrogenases that are found in *β*-oxidation. This results in a yield of 1.5 ATP per ubiquinol as ubiquinone is the direct acceptor of electrons from FADH2. The oxidation of methanol by PQQ-dependent methanol dehydrogenases (MDH) was assumed to yield 1 ATP per methanol by the oxidation of reduced cytochrome $c_{L}$. The NADH:ubiquinone oxidoreductase complex is supposed to transport 4 protons from the cytosol to the periplasm, equaling around 1 ATP [47]. During fumarate respiration in *E. coli*, the proton translocating NADH dehydrogenase (type I) is used [48] which leads to the assumption that the maximal achievable stoichiometry would be

(3)fumarate+NADH+4H+in⟶succinate+NAD++4H+out

Assuming a H^+^/ATP ratio of the ATP synthase of 4, this results in the generation of 1 ATP. The difference in the costs of NADPH and NADH depends on the ratio of their reduced and oxidized form. NADPH is usually more reduced than NADH, i.e., the NADPH/NADP^+^ ratio is thought to be a lot higher than the NADH/NAD^+^ ratio in many bacteria [49]. The ratio of the two cofactors can be balanced by the membrane-bound transhydrogenase:

(4)NADH+NADP++H+out⟶NAD++NADPH+H+in

Using this stoichiometry, all needed NADPH could be regenerated from NADH and the cofactors could be poised to the optimal ratios. The regeneration of one NADPH from NADH thus comes at the cost of 0.25 ATP (H^+^/ATP ration of 4). It also has to be stated that different organisms use different respiratory chains [39, 50] and the stoichiometry of ATP synthase may also vary, e.g., in chloroplasts, the H^+^/ATP ratio is 4.67 rather than 4 [51] or even 3.3 [46]. Therefore, the projected ATP costs (and the product-substrate yields) represent rather optimistic estimates. This does not influence the overall comparison between pathways too much, however.

As electron sources, either methanol, formate, or H_2_ was used to regenerate NADH in some of the following analyses. Methanol was oxidized via a NADH- or PQQ-dependent dehydrogenase and formaldehyde dehydrogenase. Formate was oxidized by the action of formate dehydrogenase and H_2_ via an oxygen-tolerant hydrogenase. These electron-delivering reactions are summed up in Figure S10 in the Supplementary Information. To support formate-dependent pathways with H_2_ as a substrate, the formate dehydrogenase was used in the reductive direction with NADPH (X. [52]) assuming that a NADPH-dependent version of the enzyme with equal kinetics can be found in nature or evolved in the laboratory.

## 3. Results

### 3.1. Overview of the Natural and Artificial Pathways Used in This Study

**Figure 2:**
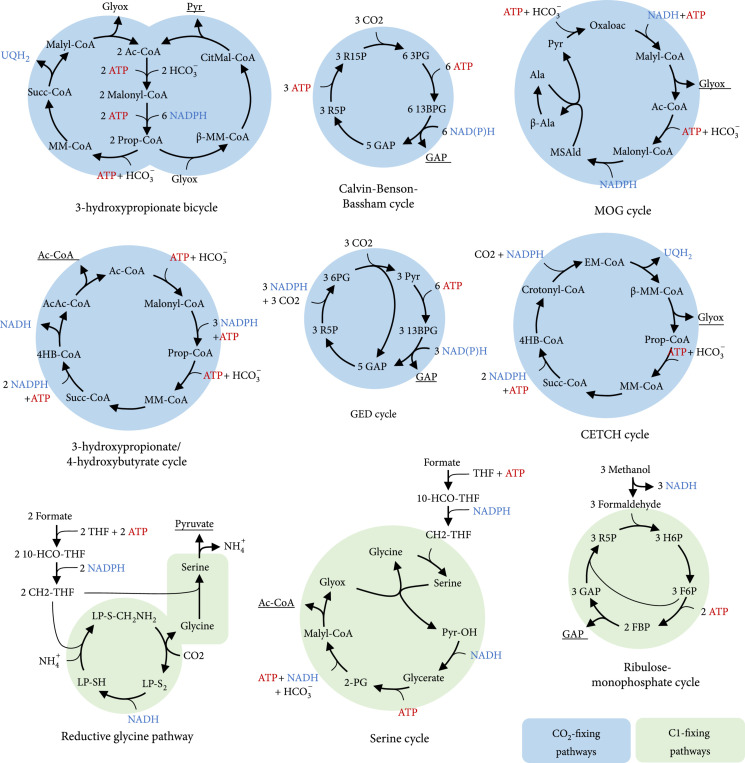
Overview of natural occurring and artificial CO_2_- and C1-fixing pathways that were integrated in the model. For simplification, reaction arrows can include multiple reactions and skip some metabolites. ADP, AMP, phosphate, water, and oxidized forms of electron carriers were also left out to improve clarity. Primary products of the pathways are underlined. Abbreviations: Ac-CoA: acetyl-CoA; Prop-CoA: propanoyl-CoA; *β*-MM-CoA: *β*-methylmalyl-CoA; CitMal-CoA: citramalyl-CoA; Pyr: pyruvate; MM-CoA: methylmalonyl-CoA; Succ-CoA: succinyl-CoA; Glyox: glyoxylate; 4HB-CoA: 4-hydroxybutyrate; AcAc-CoA: acetoacetyl-CoA; R15P: ribulose-1,5-bisphosphate; 3PG: 3-phosphoglycerate; 13BPG: 1,3-bisphophoglycerate; GAP: glyceraldehyde-3-phosphate; R5P: ribulose-5-phosphate; H6P: hexulose-6-phosphate; MSAld: malonate semialdehyde; Ala: alanine; *β*-Ala: *β*-alanine; Oxaloac: oxaloacetate; 6-PG: 6-phosphoglycerate; OH-Pyr: hydroxypyruvate; 2-PG: 2-phosphoglycerate; 10-CHO-THF: 10-formyltetrahydrofolate; CH2-THF: 5,10-methylenetetrahydrofolate; LP-S_2_: [glycine-cleavage complex H protein]-N6-lipoyl-L-lysine; LP-S-CH_2_NH_2_: [glycine-cleavage complex H protein]-S-aminomethyl-N6-dihydrolipoyl-L-lysine; LP-SH: [glycine-cleavage complex H protein]-dihydrolipoyl-L-lysine.

Nine original pathways were chosen from the literature and implemented by adding their stoichiometries and relevant kinetic and thermodynamic parameters to the SBtab model file and SBtab data file as described in Material and Methods. An overview of these pathways is given in Figure 2. We included three natural occurring, oxygen-insensitive CO_2_-fixing pathways: the Calvin-Benson-Bassham cycle (CBB cycle), the 3-hydroxypropionate bicycle (3-HP bicycle), and the 3-hydroxypropionate/4-hydroxybutyrate cycle (3-HP/4-HB cycle). For the 3-HP/4-HB cycle, two different variants have been described [53], both of which were integrated. They were designated as crenarchaeal or thaumarchaeal, respectively. Another CO_2_-fixing cycle, the reverse TCA (rTCA) operates in several organisms with an aerobic lifestyle in the genera *Nitrospira*, *Aquifex*, and among the Epsilonproteobacteria. Although the rTCA and the recently discovered roTCA variant [54] are both highly efficient, they were not included in this work because of insufficient kinetic data of the oxygen-tolerant 2-oxoglutarate:ferredoxin oxidoreductase and because it is still unclear how the necessary ferredoxin is generated under aerobic conditions. Additionally, two common C1-fixing pathways were added to the SBtab model file: the Serine cycle that is known for its efficient assimilation of formate and the Ribulose Monophosphate cycle (RuMP cycle) that uses methanol as the main substrate. The RuMP cycle exists in various variants: we integrated the sedoheptulose-1,7-bisphosphatase variant with either 2-dehydro-3-deoxy-phosphogluconate or fructose-1,6-bisphosphat aldolase. Ribulose-5-phosphate was regenerated with transketolase and not with transaldolase. Furthermore, a NAD-dependent methanol dehydrogenase (MDH) as found, e.g., in the thermophilic *Bacillus methanolicus*, and a PQQ-dependent MDH as found in many mesophilic methanotrophs were used as options in pathway analysis.

Apart from the presented natural pathways, four less common pathways that were described as potential candidates for efficient bioproduction were also included in the SBtab files: the CETCH cycle, the linear reductive glycine pathway (rGlyP), the Gnd-Entner-Doudoroff cycle (GED cycle) [28], and the aminomutase variant of the malonyl-CoA-oxaloacetate-glyoxylate (MOG) pathways [4]. Although there is a number of possible carbon fixation pathways that have been proposed or discovered in the past, the four chosen pathways have favorable characteristics for the analysis in this work. All pathways are supposed to work under aerobic conditions, and sufficient kinetic data are available. In addition, the CETCH cycle is one of the few artificial CO_2_-fixing pathways that have been implemented experimentally [8]. The rGlyP was engineered *in vivo* to support growth on formate or methanol as the sole carbon and energy source and is also the potentially most efficient aerobic formatotrophic pathway. The GED cycle shows structural resemblance to the CBB cycle with the carboxylation of a C5 sugar and the subsequent cleavage into two C3 bodies, followed by regeneration of the C5 sugar. The key reaction of the cycle, the carboxylation of ribulose-5-phosphate which was characterized well, avoids photorespiration, and the activity of the whole pathway was already shown *in vivo*. The artificial MOG pathways were described as an optimal compromise between kinetics and stoichiometry [4], and therefore, one of its variants (using alanine aminomutase) was used as a benchmark for the predictions as a purely *in silico*-designed pathway. Exotic pathways that rely on high concentrations of formaldehyde [19–21] and pathways that are variations of existing pathways ([55]; H. [17]) were also not considered. The detailed pathway maps that were integrated here, as well as the corresponding metabolites and enzymes, the respective kinetic data, and their literature sources, can be found in the Supplementary Information in Supplementary Figures S1-10 and Table S2.

### 3.2. Design of New Pathways Related to the Reverse TCA Cycle

**Figure 3:**
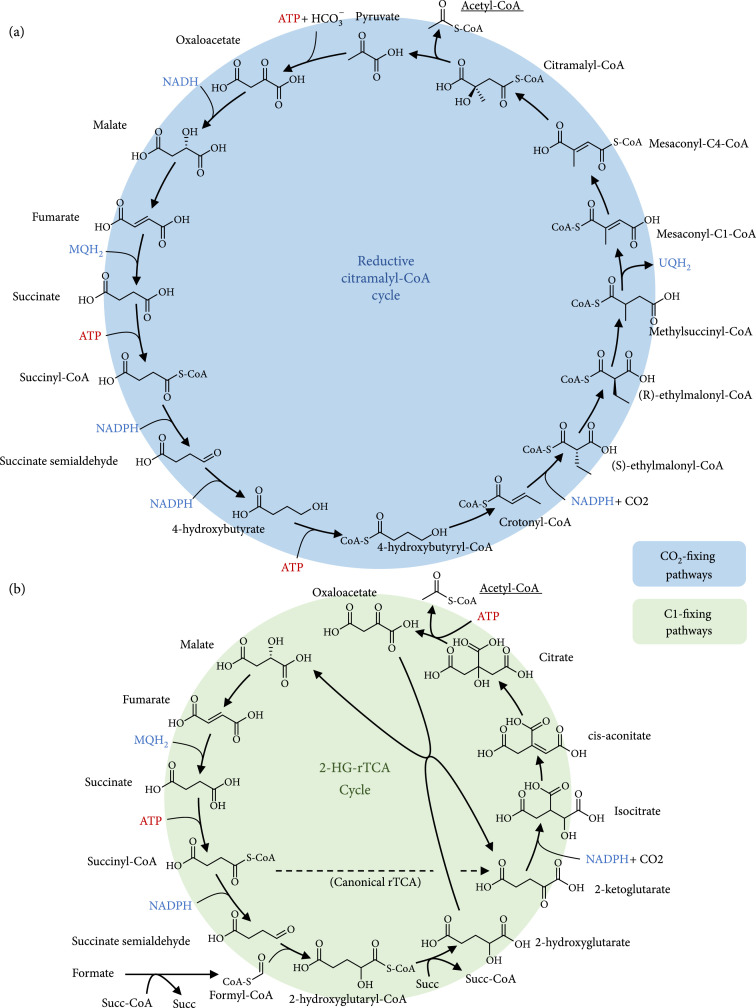
Overview of (a) the reductive citramalyl-CoA cycle (rCCC) and (b) the 2-hydroxyglutarate-reverse TCA cycle (2-HG-rTCA cycle) that were designed in this work. For simplification, reaction arrows can include multiple reactions and skip some metabolites. ADP, AMP, phosphate, water, and oxidized forms of electron carriers were also left out to improve clarity. Abbreviations: Succ-CoA: succinyl-CoA; Succ: succinate.

In addition to pathways that were described by other authors or found in nature, we also designed two completely new pathways that are insensitive to oxygen and feature a favorable stoichiometry: the reductive citramalyl-CoA cycle (rCCC) and the 2-hydroxyglutarate-reverse TCA cycle (2-HG-rTCA cycle) that are depicted in detail in Figure 3. Both pathways share a common structure with the reverse TCA cycle that is regarded as one of the most efficient pathways for CO_2_ fixation [4, 21, 54] but is limited to a very specialized group of microorganisms. The rCCC is a composite of existing pathways: it combines one part of the reverse TCA cycle (from oxaloacetate to succinyl-CoA), one part of the CETCH cycle (from succinyl-CoA to mesaconyl-C1-CoA), and one part of the 3-HP bicycle (from mesaconyl-C1-CoA to pyruvate and acetyl-CoA). The cycle is closed by pyruvate carboxylase, a carboxylating enzyme with high activity that is supposed to be superior to RuBisCO ([4, 26], and [12]). The cycle only needs 3 ATP to produce 1 acetyl-CoA and is thus even more efficient than the thaumarchaeal 3-HP/4-HB cycle that was described as the most ATP-efficient aerobic CO_2_-fixing pathway [53] and needs 4 ATP to produce 1 acetyl-CoA. The 2-HG-rTCA cycle, the second pathway that was designed, has an even higher overlap with the reverse TCA cycle (from ketoglutarate to succinyl-CoA in the reductive direction). Hence, the only reaction that is missing from the rTCA cycle is the ferredoxin-dependent 2-oxoglutarate synthase which is also the only oxygen-sensitive enzyme of the cycle. This carboxylating enzyme was replaced in the 2-HG-rTCA cycle by an enzyme that extends the C4-carboxylic acid not by CO_2_ but with formyl-CoA in the following fashion:

(5)succinate semialdehyde+formyl‐CoA⟶2‐hydroxyglutaryl‐CoA

This reaction is catalyzed by a 2-hydroxyacyl-CoA lyase, an enzyme that has recently gained attention as an enzyme to assimilate C1 bodies [19, 56]. In their original work, Chou et al. use this enzyme to extend formaldehyde with formyl-CoA. Besides the high toxicity of formaldehyde, the enzyme also has a very high $K_{m}$ for this substrate, making it less than ideal. Longer chain length aldehydes show lower $K_{m}$ values which help to limit toxic aldehyde concentrations. We assumed the $K_{m}$ value and turnover number for succinate semialdehyde to be the geometric mean of propanal and pentanal since succinate semialdehyde has 4 carbon atoms. For future applications, this is probably a conservative estimate since the enzyme was not yet engineered for the shorter chained substrates and is actually supposed to catalyze the reverse reaction. In fact, enzyme engineering was successfully used to reduce the $K_{m}$ of formaldehyde of this enzyme [56]. So higher affinities and turnover numbers for succinic semialdehyde should be possible. In the 2-HG-rTCA cycle, 2-hydroxyglutaryl-CoA is then supposed to transfer its CoA group to an acceptor carboxylic acid. Here, it was assumed to be transferred to succinate. Considering the kinetics, it was estimated that similar kinetic parameters as determined for succinyl-CoA:malate CoA-transferase can be achieved. In a recent study, it could be demonstrated that highly active 2-hydroxyacyl-CoA transferases can be found in nature [57], so from a design perspective, this should not be a problematic reaction step. Next, 2-hydroxyglutarate is oxidized to 2-ketoglutarate. This thermodynamically difficult step (at least with NAD^+^ as the oxidizing agent) is catalyzed by the lactate-malate transhydrogenase that also accepts 2-hydroxyglutarate as a substrate, with a lower specificity and rate, however. Again, this is a conservative estimation as the enzyme has not yet been optimized for the substrates. From 2-ketoglutarate on, the pathway follows the traditional reverse TCA cycle.

The detailed pathway maps, as well as the corresponding enzymes, the respective kinetic data, and their sources can be found in the Supplementary Information in Figure S4 and Table S2. An overview on the overall stoichiometries of all pathways is listed in Tables S4 and S5 in the Supplementary Information.

### 3.3. Design of a New Phosphatase-Less Calvin-Benson-Bassham Cycle Variant

As a thought experiment to analyze and to understand the natural design of the CBB cycle, a hypothetically augmented version of the pathway was designed that uses 2 mol ATP less for the production of 1 mol C3 sugar. This is achieved by replacing the irreversible phosphatase reactions with phosphotransferase reactions in the following fashion:

(6)seduheptulose‐1,7‐bisphosphate+phosphate⇌seduheptulose‐7‐phosphate+pyrophosphatefructose‐1,6‐bisphosphate+phosphate⇌fructose‐6‐phosphate+pyrophosphate2 ribulose‐5‐phosphate+2 pyrophosphate⇌2 ribulose‐1,5‐bisphosphate

**Figure 4:**
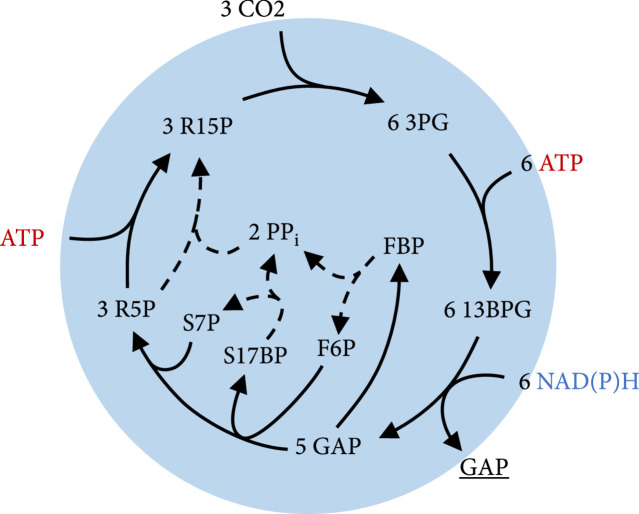
Overview of the phosphatase-less Calvin-Benson-Bassham cycle variant that was designed in this work. Dashed lines represent the phosphotransferase reactions that were added which replace the phosphatase reaction and transfer 2 phosphate groups to ribulose-5-phosphate (R5P). For simplification, reaction arrows can include multiple reactions and skip some metabolites. ADP, AMP, phosphate, water, and oxidized forms of electron carriers were also left out to improve clarity. Abbreviations: R15P: ribulose-1,5-bisphosphate; 3PG: 3-phosphoglycerate; 13BPG: 1,3-bisphosphoglycerate; GAP: glyceraldehyde-3-phosphate; FBP: fructose-1,6-bisphosphate; F6P: fructose-6-phosphate; S17BP: seduheptulose-1,7-bisphosphate; PP_i_: pyrophosphate; S7P: seduheptulose-7-phosphate; R5P: ribulose-5-phosphate.

This net phosphate transfers are exergonic under standard conditions with $\Delta_{r}G^{\prime {}^{\circ}}=-11\pm 4\;\text{kJ}/\text{mol}$ for the transfer of phosphate from seduheptulose-1,7-bisphosphate to ribulose-5-phosphate and with $\Delta_{r}G^{\prime {}^{\circ}}=-4\pm 5\;\text{kJ}/\text{mol}$ for transfer of the phosphate from fructose-bisphosphate to ribulose-5-phosphate. While the phosphotransferase reaction with fructose-1,6-bisphosphate is known to operate in plants and even replaces the fructose-1,6-bisphosphate phosphatase in some organisms [58], the phosphorylation of ribulose-5-phosphate has not been observed to our best knowledge. A simplified pathway map of this imaginary cycle is illustrated in Figure 4, and a more detailed map specifying the enzymes for each reaction can be found in the Supplementary Information in Figure S5.

Enzymes were found that are able to catalyze the described phosphate transfer reactions via pyrophosphate [59] in a promiscuous fashion. Nevertheless, enzymes that can directly transfer the phosphate group from one sugar to the next might also appear in nature and thus might avoid possible regulatory problems associated with the use of pyrophosphate as a substrate.

### 3.4. Connecting Products of Pathways with Each Other in a Modular Fashion

The primary products of the pathways presented in the last sections are different for each pathway when looking at the illustrated stoichiometries (Figures 2–4):

(i) The 3-HP/4-HB cycle, the rCCC, the 2-HG-rTCA cycle, and the Serine cycle produce acetyl-CoA
(ii) The CBB cycle and RuMP cycle produce glyceraldehyde-3-phosphate
(iii) The 3-HP bicycle and rGlyP produce pyruvate
(iv) The CETCH cycle produces glyoxylate as the primary product

This is a challenge for the comparison of the pathways as it is unclear how the “value” of a produced metabolite relates to another. To solve this, pathways were added to the SBtab model file that connect the primary products with each other. These additional connecting pathways will be called “connecting modules” as they can modularly be combined with the original primary pathways. The connecting modules that were implemented are described in the following sections and summed up with their net stoichiometries in Table 1. Again, the respective reactions, their stoichiometries, and kinetic parameters were added to the SBtab model and data files. Some of these connections are obvious at first: pyruvate can be turned into glyceraldehyde-3-phosphate via gluconeogenesis, for instance. Glyceraldehyde-3-phosphate can inversely be converted back to pyruvate or oxaloacetate via the last steps of glycolysis. For other primary pathway products, the situation is more difficult, however: assimilation of acetyl-CoA to C4 dicarboxylic acids is done by many organisms with the glyoxylate cycle that follows stoichiometry:

(7)2 acetyl‐CoA+NAD++UQ⟶malate+NADH+UQH2+2CoA

where UQ is ubiquinone and UQH_2_ is ubiquinol.

**Table 1:** Modules connecting pathways that were implemented in the SBtab model file.

Originating pathway ^e^	Stoichiometry (main substrates underlined, main products bold)	$\Delta_{r}G^{\prime 0}$ (kJ/mol)
*Glyoxylate-converting modules*		
*β*-Hydroxyaspartate cycle	2 glyoxylate+NADH⟶**oxaloacetate**+NAD^+^	−71±6
3-HP-bicycle (modified)	glyoxylate+3NADPH+2HCO_3_^-^+ATP⟶ ⟶**oxaloacetate**+3NADP^+^+ADP+P_i_	−28±13
Reverse glyoxylate shunt	Glyoxylate+ATP+MQH_2_+NADH+CoA+2H^+^_in_⟶ ⟶**acetyl-CoA**+ADP+P_i_+MQ+NAD^+^+2H^+^_out_	−15±7 ^a^
Serine cycle	Glyoxylate+formate+NADH+NADPH+HCO_3_^-^+2ATP⟶ ⟶**oxaloacetate**+NAD^+^+NADP^+^+2ADP+2P_i_	−76±8
Glyoxylate carboligase/glycerate dehydrogenase	2 glyoxylate+NADH⟶**glycerate**+NAD^+^+CO_2_	−46±8
*Acetyl-CoA-converting modules*		
Ethylmalonyl-CoA pathway (modified)	2 acetyl-CoA+2NADPH+0.5NAD^+^+UQ+MQ+HCO_3_^-^+CO_2_+2H^+^_out_⟶ ⟶1.5 **oxaloacetate**+2NADP^+^+0.5NADH+UQH_2_+MQH_2_+2CoA+2H^+^_in_	−65±13 ^a^
3-HP/4-HB cycle (modified) ^b^	Acetyl-CoA+3ATP+3NADPH+NAD^+^+MQ+2HCO_3_^-^+2H^+^_out_⟶ ⟶**oxaloacetate**+3ADP+3P_i_+3NADP^+^+NADH+MQH_2_+CoA+2H^+^_in_	−101±14 ^a^
Reductive citramalyl/ethylmalonyl-CoA pathway	Acetyl-CoA+HCO_3_^-^+2NADPH+UQ⟶ ⟶**pyruvate**+2NADP^+^+UQH_2_+CoA	−39±9
*Pyruvate-converting modules* ^c^		
4-HB pathway^b^ (+pyruvate carboxylase)	Pyruvate+2CoA+3ATP+HCO_3_^-^+MQH_2_+2NADPH+2H^+^_in_⟶ ⟶2 **acetyl-CoA**+3ADP+3P_i_+MQ+2NADP^+^+2H^+^_out_	−42±10 ^a^
MCG-like cycle^d^ (+pyruvate carboxylase)	Pyruvate+3ATP+HCO_3_^-^+2CoA+3NADH⟶ ⟶2 **acetyl-CoA**+3ADP+3P_i_+3NAD^+^	−115±8
*General modules*		
Glycolysis (starting from GAP)	Glyceraldehyde-3-phosphate+ADP+NAD(P)^+^+HCO_3_^-^⟶ ⟶**oxaloacetate**+NAD(P)H+ATP	−49±6
Gluconeogenesis (starting from oxaloacetate)	Oxaloacetate+2ATP+NADH⟶ ⟶**glyceraldehyde-3-phosphate**+2ADP+P_i_+NAD^+^+CO_2_	28±6

^a^The free energy was calculated assuming a transmembrane potential of -150 mV. ^b^This module exists in two variants (like the pathway it is derived from), depending on whether an AMP- or ADP-producing 3-hydroxypropyl-CoA/4-hydroxybutyryl-CoA synthase is used or not. In the here presented form, it corresponds to the crenarchaeal form. Both variants were considered, however, for the following analyses. ^c^In addition to the modules listed here, single reactions such as the pyruvate dehydrogenase complex or the pyruvate carboxylase were also used to connect metabolites. ^d^This cycle has the same working principle as the malyl-CoA-glycerate cycle (MCG cycle) presented in Yu et al. [66]. Instead of turning 2 glyoxylate into glycerate which is then converted to oxaloacetate, this cycle directly uses the *β*-hydroxyaspartate cycle to turn 2 glyoxylate into oxaloacetate. A detailed pathway map can be found in Supplementary Figure S8B. ^e^Original pathway in this context relates to the pathway in which the respective enzymes are usually found. Many of the connecting modules are subnetworks of the full, original pathways.

**Figure 5:**
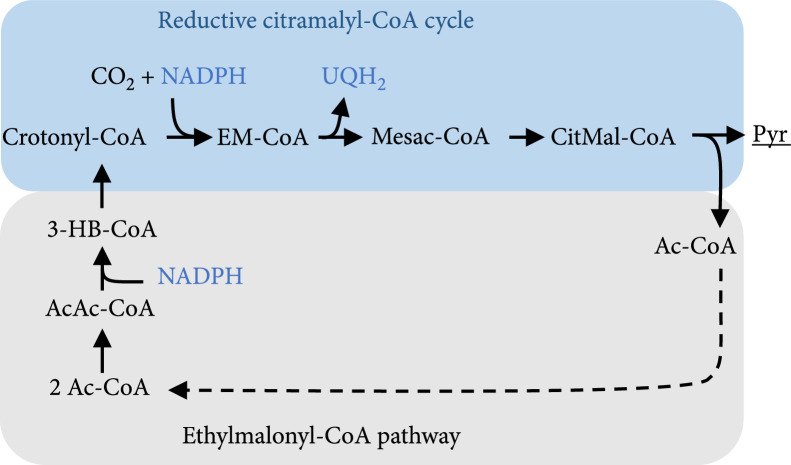
Design of a pathway to convert acetyl-CoA (Ac-CoA) to pyruvate (Pyr) using parts of the reductive citramalyl-CoA cycle (blue) and the ethylmalonyl-CoA pathway (grey). The dashed arrow denotes the possible recycling of acetyl-CoA for the first reaction of the ethylmalonyl-CoA pathway. Abbreviations: EM-CoA: ethylmalonyl-CoA; Mesac-CoA: mesaconyl-CoA; CitMal-CoA: citramalyl-CoA; Ac-CoA: acetyl-CoA; Pyr: pyruvate; AcAc-CoA: acetoacetyl-CoA; 3-HB-CoA: 3-hydroxybutyryl-CoA.

This pathway is oxidative (i.e., electrons are freed) which is contrary to the reductive nature of carbon fixation. Besides, for gluconeogenesis, two equivalents of CO_2_ are produced per molecule of formed C6 sugar using the glyoxylate cycle. This is why some bacteria use different pathways for the assimilation of acetyl-CoA that are reductive and even fix CO_2_, like the ethylmalonyl-CoA pathway [60]. Archaea with the 3-HP/4-HB cycle can turn acetyl-CoA to succinyl-CoA with the 3-HP route that is an intrinsic part of their cycle [61]. In case of the rCCC, a part of the pathway can be used for the conversion of acetyl-CoA to pyruvate if 3 reactions from the ethylmalonyl-CoA pathway are added as showcased in Figure 5. This pathway module does not need ATP. Following the calculation of projected ATP costs presented in Material and Methods, the net transfer of 2 electrons from ubiquinone to NADPH comes at a projected cost of 1.25 ATP, however. This is stoichiometrically on par with the most efficient modules introduced by Bar-Even et al. [4] in their seminal work almost a decade ago as part of the MOG pathways albeit using more reactions. In contrast to their approach that uses an aminomutase or acryloyl-CoA hydratase that is more or less oxygen sensitive and features low specific activity, the design presented in Figure 5 is assembled purely from oxygen-insensitive reactions that appear in actual carbon fixation pathways in nature.

For the conversion of glyoxylate to other metabolites, microorganisms employ different strategies: glyoxylate can be turned to glycine and then sequestered by the glycine cleavage system to finally yield glycerate [62]; alternatively, 2 mol of glyoxylate can be converted to 1 mol of glycerate and CO_2_ with tartronate-semialdehyde as an intermediate [4, 63] by tartronate-semialdehyde synthase and glycerate dehydrogenase. Recently, it was found that glyoxylate can also be sequestered to oxaloacetate by the *β*-hydroxyaspartate cycle (Schada [64]). Both glycerate and oxaloacetate can easily be converted to phosphoenolpyruvate at the cost of one ATP, but glycerate needs one ATP to react to oxaloacetate (via 2-phosphoglycerate). Here, the *β*-hydroxyaspartate cycle and the pathway using tartronate-semialdehyde synthase were both implemented in the SBtab model file. The reverse glyoxylate shunt introduced by Mainguet et al. [65] was also implemented as a module that turns glyoxylate to acetyl-CoA.

Another interesting case is the conversion of pyruvate or C4 dicarboxylic acids to acetyl-CoA. Plants use the pyruvate dehydrogenase complex, an oxidative decarboxylation reaction, which is in contrast to the reductive carbon fixing CBB cycle. To capitalize on this potential, it was proposed to rather split a C4 body into two C2 bodies, e.g., in the synthetic malyl-CoA-glycerate carbon fixation pathway (MCG pathway) (H. [66]). In this pathway, malate is split into acetyl-CoA and glyoxylate. Glyoxylate is then fixed to glycerate as described earlier. Finally, malate can be regenerated from glycerate. We implemented a variant of this pathway in the SBtab model file that uses the *β*-hydroxyaspartate cycle for glyoxylate assimilation instead which results in a cycle that needs 1 mol of ATP less than the original pathway per 1 mol of acetyl-CoA. A detailed map of this MCG-like pathway can be found in the Supplementary Information in Figure S8B. Nature also has invented a way to convert C4 dicarboxylic acids to 2 acetyl-CoA as part of the 3-HP/4-HB cycle (compare Figure 2), starting from succinyl-CoA and ending in two molecules of acetyl-CoA. Both pathway modules were added to the SBtab model file. Detailed pathway maps of the connecting modules with the respective enzymes can be found in Supplementary Figures S6-S10.

### 3.5. General Comparison of CO_2_ Pathways at Atmospheric CO_2_ Concentration

Many pathway designs implemented in this work bear the potential to be superior to the pathways that are widespread in nature—at least in certain scenarios, like biotechnological applications. But how can we actually predict whether or not these pathways are feasible or even better apart from their stoichiometry? To rule out that pathways are thermodynamically and kinetically limited or even infeasible, the ECM algorithm was used which is able to factor in the thermodynamics and kinetics of the reactions based on all parameters that were collected. After assembly of all pathways and connecting modules, the SBtab model file comprised 113 reactions with 104 metabolites and 615 enzymatic parameters (before Parameter Balancing). The data file was then processed by Parameter Balancing to yield a complete and consistent parameter set for all reactions. Next, we evaluated the pathway-specific activities of all pathways with ECM towards the production of a certain metabolite.

**Figure 6:**
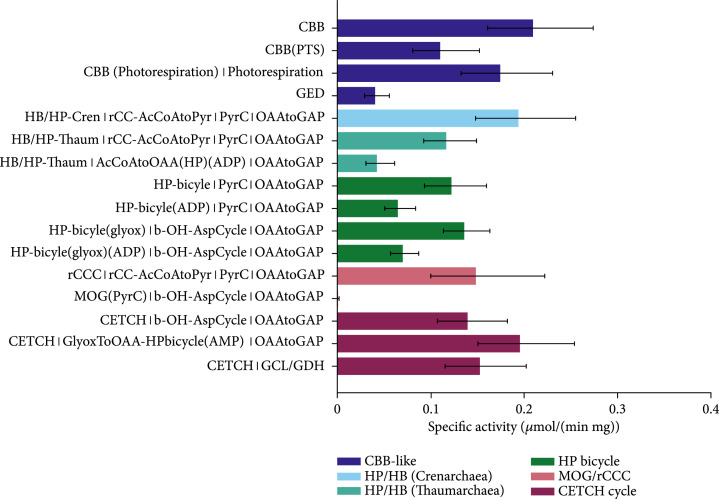
Comparison of the specific activity of natural and artificial carbon fixation pathways with the Enzyme Cost Minimization algorithm. The final product of choice was glyceraldehyde-3-phosphate in this case.. The concentrations of CO_2_ and HCO_3_^-^ were assumed to not exceed 10 *μ*M and 100 *μ*M, respectively, corresponding approximately to air saturation. Pathway-specific activities with standard deviations and the full name of the main pathways and the connecting modules to transform their primary product to glyceraldehyde-3-phosphate. Pathway abbreviations: CBB(PTS): phosphatase-free CBB cycle; HP-bicycle(glyox): glyoxylate-producing subcycle of the 3-HP bicycle; PyrC: pyruvate carboxylase; OAAtoGAP: gluconeogenesis; b-OH-AspCycle: *β*-hydroxyaspartate cycle; AcCoAtoOAA(HP): acetyl-CoA to oxaloacetate-converting module derived from the 3-HP/4-HB cycle using either ADP- or AMP-producing CoA-ester synthases; rCC-AcCoAtoPyr: acetyl-CoA to pyruvate-converting module derived from the rCCC; GlyoxToOAA: glyoxylate- to oxaloacetate-converting module based on the Serine cycle; GlyoxToGAP(GCL/GDH): glyoxylate conversion by glyoxylate carboligase and glycerate dehydrogenase. The overall stoichiometries of all pathways are listed in supplementary Table S5.

First, only the CO_2_-fixing pathways were compared for the production of glyceraldehyde-3-phosphate at atmospheric CO_2_ partial pressure as shown in Figure 6 in order to reflect the conditions found in nature without an external CO_2_ source like hydrothermal vents. The flux distributions that were assumed for each pathway are defined by the pathway stoichiometries combined with the connecting modules, as listed on the left side of Figure 6 and detailed in the Supplementary Information (available here). The source of reducing equivalents (mainly NAD(P)H) was not considered in the beginning to get a picture of the capacity and limitations of the core pathways without interference of redox metabolism. The pathway-specific activity of the CBB cycle variants was in the order of 0.25 *μ*mol min^-1^ mg^-1^ which is in line with former estimates [4]. The phosphatase-less CBB cycle (CBB(PTS)) exhibited a slightly lower activity compared to the canonical CBB cycle while the GED cycle only reached around 0.04 *μ*mol min^-1^ mg^-1^ which makes sense given the low CO_2_ partial pressure. Most pathways were predicted to have pathway activities in the same order of magnitude; some, however, were considerably slower: the MOG cycle was predicted to have only marginal activity which is in sharp contrast to the former predictions [4]. Even though the kinetic data of an oxygen-tolerant aminomutase was taken using its optimal substrate (as data for alanine is sparse), the kinetics and thermodynamics of both the aminomutase and the aminotransferase reaction are so unfavorable that the cycle is severely limited by them. Methods of enzyme engineering might solve these shortcomings in the future, however. Comparing the CO_2_-fixing pathways, the rCCC, the CETCH cycle, and the crenarchaeal 3-HB/4-HB cycle showed pathway-specific activities equal to the CBB cycle with 10% of photorespiration.

Glyceraldehyde-3-phosphate is the main product of the CBB cycle which is why this pathway was likely favored when choosing it for comparison. Therefore, the same analysis was repeated using acetyl-CoA as the product of choice which is shown in Figure S12 in the Supplementary Information. As expected, the acetyl-CoA-producing pathways performed better for the production of acetyl-CoA in this case compared to the glyceraldehyde-3-phosphate-producing ones. The general trend of pathway-specific activities is not influenced by the choice of product, however: again, the rCCC and the CETCH cycle are on par with the CBB cycle in terms of activity. For all pathways that produce C3 or C4 bodies, different connecting modules were tested that were able to generate 2 acetyl-CoA molecules from one C3 or C4. Among them, the route via 4-hydroxybutyrate from the Thaumarchaeota showed the highest activity and yield. Since the CBB cycle is mainly limited by RuBisCO, lowering the CO_2_ concentration has a dramatic impact on its pathway-specific activity which is less of a concern in alternative CO_2_-fixing pathways.

### 3.6. Evaluation of the Potential of Aerobic CO_2_- and C1-Fixing Pathways for Biotechnological Applications

In most biotechnological scenarios, bioreactors can be gasified with CO_2_, and thus, respective limitations and also photorespiration can be avoided to a certain degree which will likely have a big influence on the resulting activities. Therefore, three different electron and carbon sources that are regarded as possible platform substrates for the future bioeconomy were evaluated as feedstock for the pathways: methanol, formate, and a mixture of CO_2_ and H_2_. The concentrations of CO_2_ and carbonate were increased by a factor of 100 to simulate active gassing with CO_2_.

**Figure 7:**
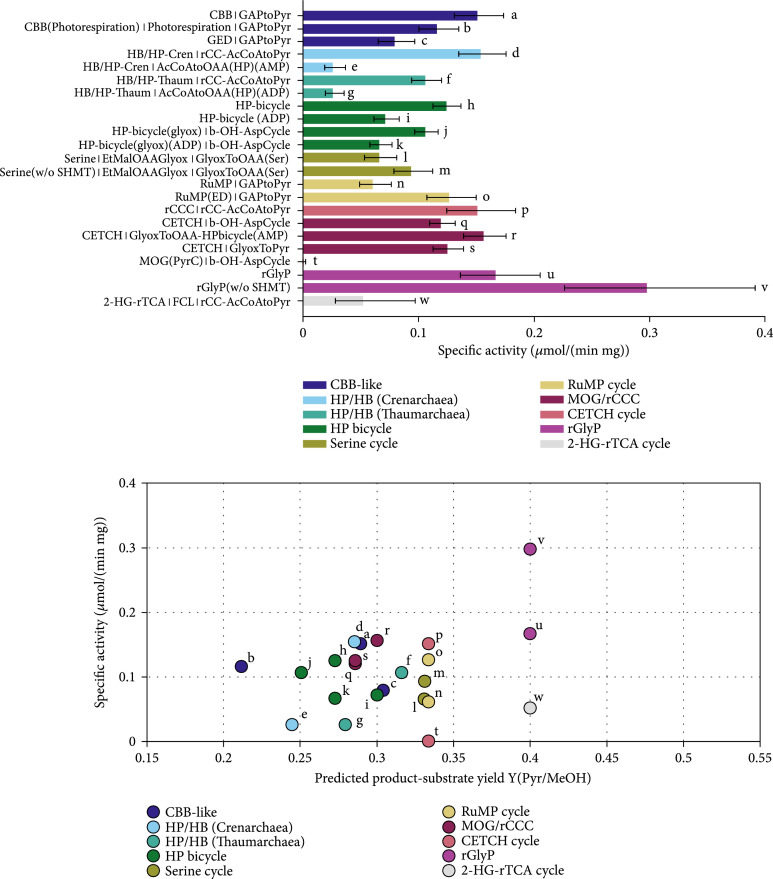
Comparison of carbon fixation pathways using methanol as a substrate for the production of pyruvate with the Enzyme Cost Minimization algorithm. “(w/o SHMT)” indicates that the cost of the serine hydroxymethyltransferase are not included in the respective pathway’s activity. The concentration of CO_2_ was assumed to be 1 mM and the concentration of HCO_3_^-^ to be 10 mM. (a) Pathway-specific activities with standard deviations and the full name of the main pathways and the connecting modules to transform their primary product to pyruvate. Pathway abbreviations: CBB(PTS): phosphatase-free CBB cycle; HP-bicycle(glyox): glyoxylate producing subcycle of the 3-HP bicycle; GAPtoPyr: glycolysis; FCL: formaldehyde:NADP+ oxidoreductase (formyl-CoA-forming); GlyoxToPyr: GCL/GDH route; b-OH-AspCycle: *β*-hydroxyaspartate cycle; AcCoAtoOAA(HP): acetyl-CoA to oxaloacetate-converting module derived from the 3-HP/4-HB cycle using either ADP- or AMP-producing CoA-ester synthases; rCC-AcCoAtoPyr: acetyl-CoA to pyruvate-converting module derived from the rCCC; GlyoxToOAA: glyoxylate- to oxaloacetate-converting module based on the Serine cycle. (b) Pathway-specific activities compared to product-substrate yield. The overall stoichiometries of all pathways and modules are listed in Table 1 and supplementary Table S4 and S5. The small letters label each pathway and correspond to the labels and the respective pathway combinations in (a).

To use methanol as an electron source, MDH was employed in this study. According to the calculations performed here, the RuMP cycle is heavily limited by the NAD-dependent MDH reaction which is thermodynamically and kinetically very unfavorable. Also, the forward rate constant at a temperature of 37°C was taken which is a lot lower than the temperature of its native host [67]. Apart from the unfavorable kinetics of the MDH, the RuMP cycle using NAD-dependent MDH was only thermodynamically feasible if the upper and lower bounds for NAD/NADH and NADP/NADPH were extended further by a factor of 10. It remains enigmatic how microorganisms like *Bacillus methanolicus* use this enzyme at a high specific rate, but the elevated temperature requirement might play a role here. Alternatively, the enzyme kinetics and maybe even the thermodynamics could be fundamentally different *in vivo* since, for engineered *E. coli* with a functional RuMP cycle, it was speculated that formaldehyde was accumulating, and thus, the MDH was not the limiting factor (F. Y. H. [18]). The PQQ-dependent MDH catalyzes an exergonic reaction, and thus, the pathways with this enzyme are in general predicted to have a higher specific activity, but with a lower product yield as the electrons are directly channeled to the respiratory chain. Indeed, all pathways using the NAD-dependent MDH were limited by the methanol oxidation reaction rather than by the enzymes of the pathways themselves in our calculations. Therefore, only the results with the PQQ-dependent MDH are shown in Figure 7. The NAD-dependent specific activities and product-substrate yields can be found in the supplement in Figure S13, though. Considering the calculations with the PQQ-dependent MDH, the rGlyP (w/o SHMT) showed the highest specific activity and yield. The “(w/o SHMT)” label denoted that the serine hydroxymethyltransferase was not included when calculating the pathway-specific activity. As already noted by others [68], the kinetics of this enzyme seem to be estimated inaccurately by the usual assays which is why the enzyme accounted for a major fraction of enzyme demand in the respective pathways. The rGlyP (w/o SHMT) showed a pathway-specific activity around twice as high as the next best pathway, albeit with high uncertainty. This can be attributed to the small number of reactions steps and the favorable thermodynamics and kinetics of the rGlyP although it has to be pointed out that the kinetic parameters from the literature were mainly based on activity measurements with unnatural substrates. Kinetic data of the assembled glycine cleavage system, especially in the carboxylating direction, are sparse. Additionally, eQuilibrator had trouble estimating the free energy of the carboxylation reaction that is catalyzed by the P-protein ($\Delta_{r}G^{\prime {}^{\circ}}=-17.3\pm 15.9\;\text{kJ}/\text{mol}$):

(8)CO2+aminomethyldihydrolipoamide‐cofactor⟶glycine+lipoamide‐cofactor

As this is one of the key reactions and we also did not find a value for the forward kinetic constant, this standard deviation has a crucial impact on the total estimation of pathway-specific activity. All in all, the estimation for the rGlyP are consequently not completely trustworthy which stresses how urgently kinetic data for this system are needed to make accurate predictions. Disregarding the rGlyP, the rCCC, the 2-HG-rTCA cycle, and the CETCH cycle were paretooptimal for both criteria. The 2-HG-rTCA cycle in conjunction with the acetyl-CoA conversion module from the rCCC has a very low ATP-requirement per product. Although it has a lower pathway activity, it might still be a promising route, considering that conservative assumptions were made about the kinetic parameters of the enzymes.

**Figure 8:**
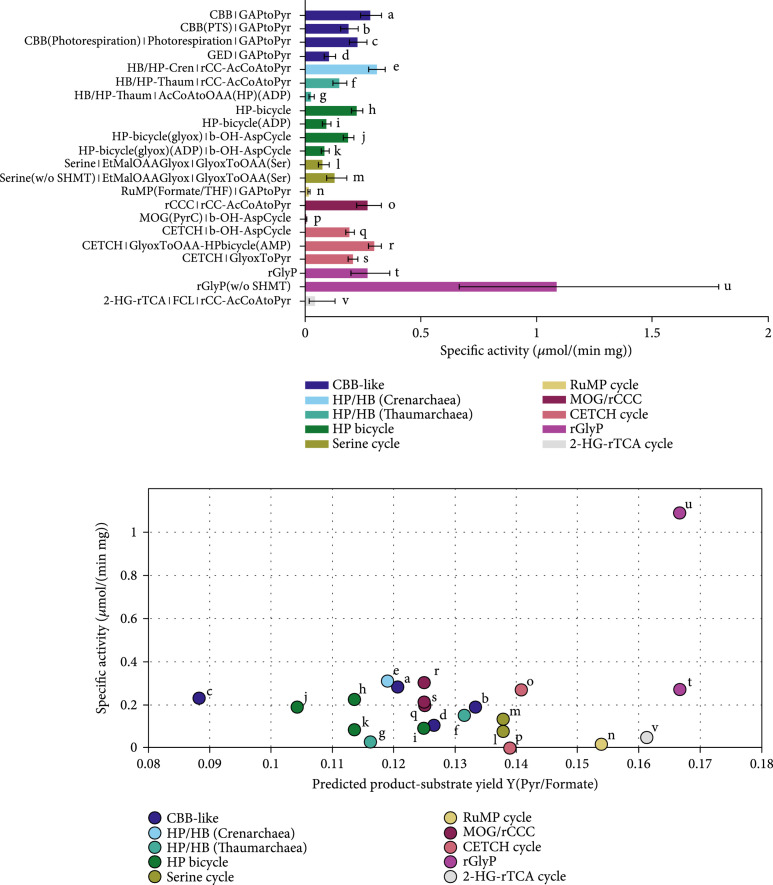
Comparison carbon fixation pathways using formate as a substrate for the production of pyruvate with the Enzyme Cost Minimization algorithm. “(w/o SHMT)” indicates that the cost of the serine hydroxymethyltransferase is not included in the respective pathway’s activity. The concentration of CO_2_ was assumed to be 1 mM and the concentration of HCO_3_^-^ to be 10 mM. (a) Pathway-specific activities with standard deviations and the full name of the main pathways and the connecting modules to transform their primary product to pyruvate. Pathway abbreviations: CBB(PTS): phosphatase-free CBB cycle; HP-bicycle(glyox): glyoxylate producing subcycle of the 3-HP bicycle; GAPtoPyr: glycolysis; FCL: formaldehyde:NADP+ oxidoreductase (formyl-CoA-forming); GlyoxToPyr: GCL/GDH route; b-OH-AspCycle: *β*-hydroxyaspartate cycle; AcCoAtoOAA(HP): acetyl-CoA- to oxaloacetate-converting module derived from the 3-HP/4-HB cycle using either ADP- or AMP-producing CoA-ester synthases; rCC-AcCoAtoPyr: acetyl-CoA- to pyruvate-converting module derived from the rCCC; GlyoxToOAA: glyoxylate to oxaloacetate converting module based on the Serine cycle. (b) Pathway-specific activities compared to product-substrate yield. The overall stoichiometries of all pathways and modules are listed in Table 1 and supplementary Table S4 and S5. The small letters label each pathway and correspond to the labels and the respective pathway combinations in (a).

When formate was used as the substrate of choice, NADH was regenerated via formate dehydrogenase, a highly active enzyme in this reaction direction. Therefore, the pathway activity is in general less limited by the supply of reducing equivalents compared to methanol. The RuMP cycle needs formaldehyde which is difficult to produce from formate because formaldehyde oxidation with NAD^+^ is almost irreversible. Likewise, reduction of formyl-CoA to formaldehyde that has been proposed by others [19, 21] was strongly limited by the formaldehyde: NADP+ oxidoreductase (CoA-acetylating) (data not shown). The only route that was functional in our simulation was the production of formaldehyde from 5,10-methylenetetrahydrofolate by the combined action of L-serine formaldehyde-lyase (glycine-forming) and 5,10-methylenetetrahydrofolate:glycine hydroxymethyltransferase. The former enzyme was also proposed for an optimized methanol assimilation pathway recently [20]. This variant of the RuMP cycle is denoted as RuMP (formate/THF). Compared to the general analysis in the previous section, the pathways’ activity profited from the higher concentration of CO_2_/HCO_3_^-^ which can be seen in Figure 8. The RuMP cycle starting from formate showed little activity and performed worse than the 2-HG-rTCA cycle and the rGlyP as it was again limited by the reactions supplying the formaldehyde.

**Figure 9:**
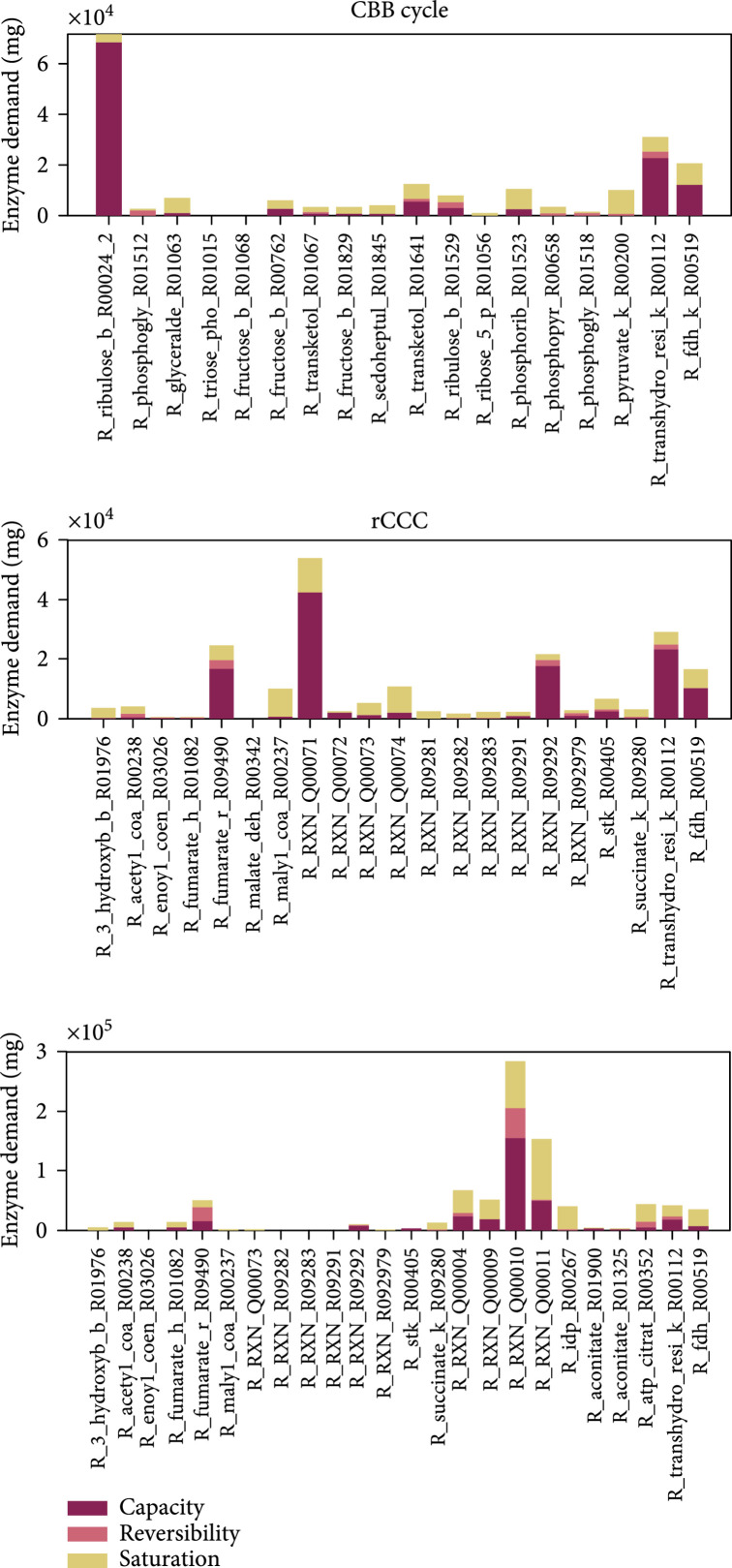
Enzyme demands to sustain a total pathway activity of 1 mmol product per second. Contribution of the capacity, the reversibility, and the saturation with substrates or products of each reaction to the demand of enzymes for the CBB cycle, the rCCC, and the 2-HG-rTCA cycle. The figure follows the wording of Noor et al. [29]. “Capacity”: demand of enzyme caused by a limitation by the catalytic rate constant; “Reversibility”: extra amount of enzyme needed because of a backward flux; “Saturation”: additional enzyme necessary because of undersaturation with a substrate or oversaturation with a product. The values present the optimized state as predicted by the ECM algorithm assuming a CO_2_ concentration of 10 *μ*M and HCO_3_^-^ concentration of 100 *μ*M. Glyceraldehyde-3-phosphate was chosen as a product for the pathways. Reaction names correspond to their identifiers in the SBtab model file (Supplementary file Reactions_Composite22_model.tsv (available here)).

Looking at the single contributions of the reactions to the enzyme demand, the Enzyme Cost Minimization algorithm makes consistent predictions as can be seen in Figure 9. The formate-based pathway analysis was well suited to evaluate the new proposed pathways in this regard because they were not supposed to be limited by the electron supply (i.e., formate) in this case. For the CBB cycle, RuBisCO (identifier “R_ribulose_b_R00024_2”) is the enzyme with the highest demand in the cycle where most of the demand is due to the relatively low turnover number (represented by “Capacity”), and a small fraction is caused by undersaturation with CO_2_ (represented by “Saturation” which is considerably higher when the CO_2_ partial pressure is low). In comparison to the CBB cycle, the new rCCC and the 2-HG-rTCA cycle (Figure 9) do not have a single limiting enzyme, but the enzyme demand is more evenly distributed among the enzymes. For the rCCC, the enzymes with the highest demand are the pyruvate carboxylase (Q00074) and the 4-hydroxybutyrate-CoA ligase (ADP-forming) (Q00071). While the pyruvate carboxylase is limited by the low concentration of carbonate, the 4-hydroxybutyrate-CoA ligase is supposed to be slower and thermodynamically more challenging than the AMP-forming variant [53]. Considering the 2-HG-rTCA cycle, the enzyme with the highest demand is the succinyl-CoA:2-hydroxyglutarate CoA-transferase (identifier Q00010). As noted before, this enzyme activity is just a side reaction of the succinyl-CoA:(S)-malate CoA-transferase, and thus, with a more specific enzyme, the pathway activity will be higher. In the 2-HG-rTCA cycle, the isocitrate dehydrogenase (carboxylating) is severely limited by the low availability of CO_2_ ($K_{m}$ of 1.3 mM) and is therefore the second costliest enzyme in the cycle. Taken together, the estimates for the new pathways show a lot of potential for the new artificial pathways. The contributions of each reaction on the other pathways can be found in the Supplementary Information in Figures S17-S24.

**Figure 10:**
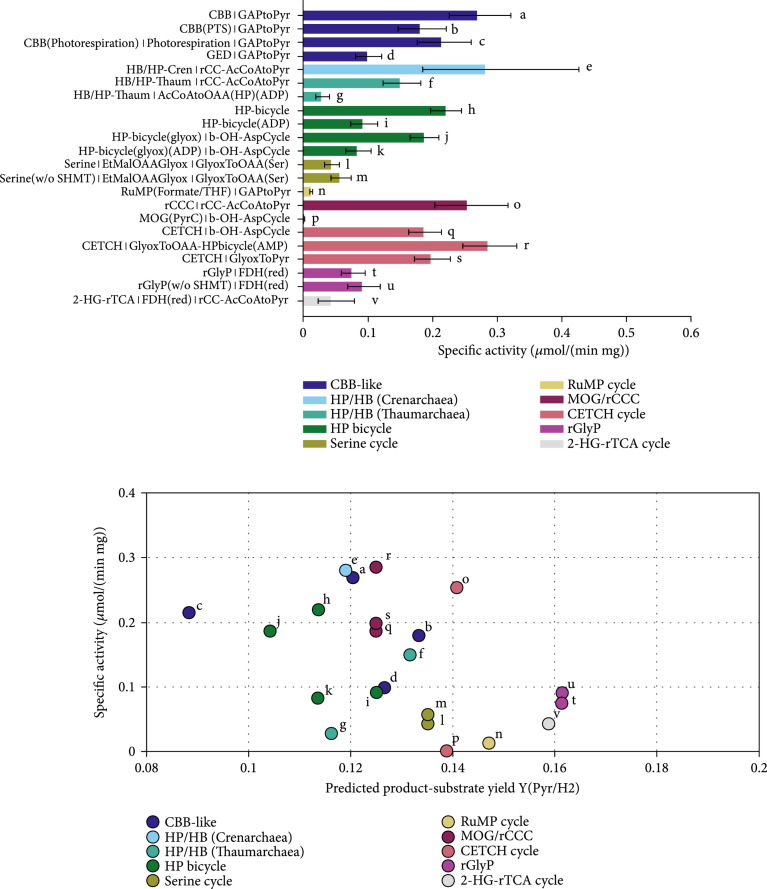
Comparison carbon fixation pathways using H_2_/CO_2_ as a substrate for the production of pyruvate with the Enzyme Cost Minimization algorithm. “(w/o SHMT)” indicates that the cost of the serine hydroxymethyltransferase is not included in the respective pathway’s activity. The concentration of CO_2_ was assumed to be 1 mM and the concentration of HCO_3_^-^ to be 10 mM. (a) Pathway-specific activities with standard deviations and the full name of the main pathways and the connecting modules to transform their primary product to pyruvate. Pathway abbreviations: CBB(PTS): phosphatase-free CBB cycle; HP-bicycle(glyox): glyoxylate-producing subcycle of the 3-HP bicycle; GAPtoPyr: glycolysis; FCL: formaldehyde:NADP+ oxidoreductase (formyl-CoA-forming); GlyoxToPyr: GCL/GDH route; b-OH-AspCycle: *β*-hydroxyaspartate cycle; AcCoAtoOAA(HP): acetyl-CoA- to oxaloacetate-converting module derived from the 3-HP/4-HB cycle using either ADP- or AMP-producing CoA-ester synthases; rCC-AcCoAtoPyr: acetyl-CoA- to pyruvate-converting module derived from the rCCC; GlyoxToOAA: glyoxylate- to oxaloacetate-converting module based on the Serine cycle. (b) Pathway-specific activities compared to product-substrate yield. The overall stoichiometries of all pathways and modules are listed in Table 1 and supplementary Table S4 and S5. The small letters label each pathway and correspond to the labels and the respective pathway combinations in (a).

For the biotechnological scenario with a mixture of CO_2_ and H_2_, an oxygen-tolerant soluble hydrogenase (kinetic data from *C. necator* H16) was assumed to regenerate NADH. In order to also include formate-dependent pathways, the formate dehydrogenase was run in the reductive direction as described or proposed by others (X. [26, 52], and [12]). This is only possible at elevated CO_2_ concentrations, however, as activity at atmospheric concentrations showed only marginal activity when formate dehydrogenase was run in “reverse.” The kinetic data of the *C. necator* enzyme were taken as it exhibited reasonably high activity in the reductive direction with NADH and is stable under aerobic conditions. It was assumed that a NADPH-dependent variant can be found in nature, engineered and/or evolved with similar characteristics, and that diaphorase activity can be neglected at high CO_2_ concentrations. For the RuMP cycle, again the formate/THF variant was evaluated. At high CO_2_/HCO_3_^-^ and with NADPH as the reducing equivalent, the formate dehydrogenase could indeed reduce CO_2_ to formate to power the formate-dependent pathways according to our calculations as shown in Figure 10. The activity of these pathways was in general lower, however, than when formate was already present (Figure 8). The rGlyP still showed acceptable activity with the highest yield among all pathways making it a viable option even for H_2_/CO_2_-based processes. Apart from that, the general trend of the performance of different pathways was unchanged compared to formate. Again, the 2-HG-rTCA cycle, rCCC, and CETCH cycle were pareto-optimal for both criteria when not considering the rGlyP. Given the high rate constant of the hydrogenase, the supply of reducing equivalents was not the limiting factor in this case in contrast to the methanol-based calculation.

All three scenarios presented in this section have also been performed with acetyl-CoA as the product of choice (compare Supplementary Figures S14-S16). Once more, the acetyl-CoA-generating pathways performed better than the production of pyruvate as in Section 3.5. In particular, the Serine cycle had a much improved activity, indicating that for this cycle, the ethylmalonyl-CoA pathway was the limiting part in the calculations for GAP production. However, the general trend of the pathways’ performances did not change much.

## 4. Discussion

### 4.1. Strengths, Weakness, and Niches of Carbon Fixation Pathways

In the simulations described in the last sections, it could be seen that the orders of magnitude of both specific activity and product-substrate yield are similar for most pathways. Given the uncertainties of the enzymatic parameters that were used and the fact that enzyme kinetics were measured in vitro, there is no clear “optimal” pathway. It is rather likely that most pathways could have a niche in nature in which they are competitive which is in agreement with the sheer existence of many of them. The Serine cycle was very efficient and highly active for production of acetyl-CoA from formate, and the RuMP pathway was very efficient with methanol as a substrate which fits their natural roles. In the biotechnological scenarios described in the last section, the 3-HP/4-HP cycle and the 3-HP bicycle were predicted to be as active as the CBB cycle variants. Since these pathways occur in aquatic microorganisms and all carboxylation reactions are dependent on HCO_3_^-^, they can profit from the option to actively transport carbonate into the cell. For a similar concentration mechanism for CO_2_, dedicated (micro-) compartments are necessary. Additionally, carbonate concentrations at a pH greater than 7—as found in most aquatic habitats—are generally higher than the CO_2_ concentrations at saturation, and therefore, the Michaelis constants of carbonate-fixing enzymes are not as restricted to low values.

The alternative and engineered carbon-fixing pathways match the activity of the CBB cycle but yield more product per substrate: the rCCC and the CETCH cycle outperform the CBB cycle in this regard. With formate as a substrate, the rGlyP was superior to the Serine cycle. Taken together, the rCCC, CETCH cycle, and rGlyP pathways have a high potential for the implementation in living organisms to create chassis organisms for the fixation of CO_2_ and C1 compounds in Industrial Biotechnology. The GED pathway and the phosphatase-less CBB cycle were shown to not fall short off the CBB cycle in specific activity and might be interesting alternatives if better enzymes can be found for the critical reactions. However, the aminomutase variant of the MOG cycle seemed to be infeasible judging from our calculations. All model substrates—formate, methanol, and H_2_/CO_2_—showed good specific activities and hence are promising options for sustainable bioprocesses.

For future applications, the CO_2_-fixing pathways might also be implemented in crop plants which can be regarded as the Holy Grail of plant biotechnology and might boost crop productivity. To achieve this, the plant and chloroplast genome would have to be radically changed on a metabolic and regulatory level which, as of today, seems out of range of the methods of Synthetic Biology. First applications will consequently rather be found be in microorganisms that can be manipulated more easily and cultivated under controlled conditions.

### 4.2. The Activity of the Calvin-Benson-Bassham Cycle Has Been Underestimated

Apart from the reductive glycine pathway which was difficult to predict because of a lack of kinetic data, most pathways actually have a pathway-specific activity in the same order of magnitude (compare Figures 6–8). This is in contrast to previous reports that concentrated on the weaknesses of the CBB cycle [4, 69–71]. So what is the reason that the activity of the CBB cycle was higher than expected? Judging from the results in this work, apart from RuBisCO, all enzymes in the CBB cycle are highly active and thermodynamics and kinetics are favorable. All other pathways were suffering either from kinetic or thermodynamic limitations or because of enzymes with equally low activity as RuBisCO. These enzymes should be better characterized in the future to exclude that they are indeed the limiting factor for the respective pathways. In Figures S17-S24 in the Supplementary Information, a detailed overview on pathway bottlenecks can be found. While the activity of the CBB cycle is higher than expected, this does not change its relatively high demand of ATP resulting in a lower product-substrate yield compared to other alternative pathways that were part of this work. The CBB cycle performed best when the glyceraldehyde-3-phosphate was chosen as the product of choice. In fact, the glyceraldehyde-3-phosphate as a product is a realistic case for comparison since plants rely on sucrose as a transport sugar. As sucrose is chemically relatively inert, it can be used in high concentrations in the conducting vascular cells of plants in which sucrose accumulates up to a concentration of one molar [72]. This “high-voltage” power transmission of the plant can hardly be realized with fats or acids. Therefore, the advantages that other carbon fixation pathways show for the production for nonsugar products might not be relevant for plants. In addition, the CBB cycle generates sugars that can be directly used as a precursor for different biomolecules, like ribose that is part DNA and RNA. It also has to be noted that only C3 photosynthesis was included in the analysis which is strongly limited by low CO_2_ concentration and low RuBisCO activity (Figure 9). Using CO_2_ concentration mechanisms and RuBisCO variants of C4 plants with a higher $K_{m}$ and $k_{\text{cat}}$, the pathway-specific activity would be significantly higher and thus probably surpass even the best designed pathways. Consequently, the CBB cycle is not as bad as commonly assumed, according to our calculations, and there is a good reason that most of the carbon fixation on this planet is facilitated by this pathway. After all, RuBisCO might not be as “bad” as previously thought as others have already noted [73].

### 4.3. Plant-Like Photosynthesis Is Not Designed to Be as ATP-Efficient as Possible

Although the CBB cycle has a higher ATP demand for 1 mol of a specific product than other pathways, this difference is actually not very high from an energetic perspective: the free burning energy of glyceraldehyde-3-phosphate is around -1500 kJ/mol, for instance, while the energy of ATP hydrolysis is around -40 kJ/mol. So the energy that is saved by more efficient pathways might not be as important as other factors which will be discussed in the next section. In fact, there are indications that ATP efficiency is not a decisive factor for the design of carbon fixation in photosynthetic organisms. The phosphatase-less CBB cycle that we introduced in this work is a good example for this. With its modified reactions, the CBB cycle would save 2 ATP per formed C3 sugar. In addition, the pathway also did not lose much of its activity, although enzymes with promiscuous activity were taken that were not specialized to run this cycle. Why has nature not made this “easy” step in evolution to optimize the CBB cycle? While it might well be possible that there are actually organisms that use this modified cycle which just have not been found yet, there is no evidence so far that this variant is used in photosynthetic organisms. Not having the irreversible phosphatase reactions might disrupt the regulation and/or robustness of the cycle which might be more important.

A second example that ATP efficiency is not the main driver in the evolution of photosynthesis can be found in C4-plant metabolism. C4 plants use a CO_2_ concentration mechanism that gives these plants a selective advantage in certain climates [74]. CO_2_ concentration in the mesophyll cells can be achieved by the actions of PEP synthase, PEP carboxylase, and malate dehydrogenase (malic enzyme variant of C4 photosynthesis):

(9)pyruvate+ATP⟶phosphoenolpyruvate+AMP+phosphatephosphoenolpyruvate+HCO3−⟶oxaloacetate+phosphateoxaloacetate+NADPH⟶malate+NADP+

This set of reactions is quite exothermic in total with $\Delta_{r}G^{\prime {}^{\circ}}=-75\pm 7\;\text{kJ}/\text{mol}$. Malate is then shuttled to the bundle sheath cells where CO_2_ is released by the malic enzyme and refixed by the CBB cycle. Although a total minimum of 15 ATP instead of 9 ATP is necessary for the formation of one C3 sugar, C4 plants easily grow faster than C3 plants in most situations. Additionally, thinking of optimal design, the first steps in a CO_2_ concentration mechanism could also be catalyzed by pyruvate carboxylase:

(10)pyruvate+ATP+HCO3−⟶oxaloacetate+ADP+phosphateoxaloacetate+NADPH⟶malate+NADP+

These reactions are also practically irreversible $\left(\Delta_{r}G^{\prime {}^{\circ}}=-35\pm 7\;\text{kJ}/\text{mol}\right)$ with the difference that one ATP is saved. In total, using pyruvate carboxylase instead of the natural occurring enzymes would hence save 3 ATP per C3 sugar in C4 plants. Again, this variant has not been found in nature although all enzymes exist in plants. Therefore, it seems that plants prefer to have a slight edge in driving force or activity instead of being more efficient ATP-wise.

### 4.4. Unconsidered Factors in Pathway Comparison

Specific activity and yields of different metabolic pathways were the main criteria for comparison in this work. While those are important factors, they are not necessarily decisive. One unconsidered factor in this context are the vitamins: interestingly, cobalamin (vitamin B12) is necessary for all CO_2_-fixing pathways except for the CBB cycle and the rGlyP. Being the most structurally complex of all vitamins, B12 biosynthesis is a complex pathway that does not occur in plants and animals. In addition, it also contains cobalt. The costs associated to biosynthesis of B12 and other important cofactors were not taken into account in this work. The other vitamins are structurally more compact, less demanding in their biosynthesis, and independent of metal ions and are needed for many other vital reactions in the metabolism as well. In the future, this requirement could be addressed by adding B12 as a metabolite and its biosynthesis to the SBtab model file if adequate data are available. This will help to evaluate whether or not B12 requirement has a critical impact.

Other unconsidered factors in the analysis presented in this work are the controllability and robustness of the pathways. Previous works have shown that these factors are an essential part of the design principles in nature [75–77]. Allosteric control of enzymes was also not included in the reaction kinetics. More dedicated models and algorithms that combine pathway performance and criteria for the robustness could be established in the future. This way, good trade-offs between the two criteria could be predicted in artificial pathways or be explained in naturally occurring pathways. New efforts in the design of artificial allosteric control of enzymes [78, 79] could even make possible the prediction and design of a regulatory layer on top of the reactivity layer in the network to build pathway designs that are optimal for both criteria.

So far, metabolite toxicity has also not been addressed in this work. Toxic metabolites in the pathways include, for example, aldehydes like formaldehyde, glyoxylate, succinate semialdehyde, malonate and semialdehyde, but also propionyl-CoA. To include this in the future, tighter constraints on these toxic compounds may be implemented, possibly based on concentrations that are actually measured within cells with a respective metabolism.

### 4.5. Implications on Future Design and Implementation of Pathways for Carbon Fixation

Although the design space for artificial CO_2_-fixing pathways was thoroughly investigated in a former study [4], we could still find several new routes that have not yet been addressed. This is probably due to missing reactions in the metabolic networks the authors used which were collected from KEGG as the remaining reactions of the 3-hydroxypropionate bicycle were just described at the same time. Likewise, side reactions that were used to construct the pathways here (2-HG-rTCA cycle and phosphatase-less CBB cycle) are also not annotated. Using more complete, updated versions of reaction stoichiometries or even extending the network on all possible reactivities with tools of retrosynthesis like RetroPath [33, 80] will possibly open up many new possible routes that should be explored in the future.

Using ECM and other advanced tools for pathway analysis can predict how to best implement a metabolic pathway based on measured data and which ones are most promising. This relies heavily on the quality of the kinetic and thermodynamic data that are used. For some sensitive parameters of reactions, there were only single studies available in which the enzymes were characterized. The ECM algorithm can predict, however, which reactions will be likely limiting the pathways and thereby gives good suggestions for enzymes that need to be better characterized and/or engineered. This stresses how important enzymology is for the understanding and the design of biochemical pathways as has also been noted by Erb recently [81]. In fact, the predictions made in this work are only possible because of the thorough characterization of all the enzymes by others. In the future, omics data in addition to more enzyme kinetics should be integrated into the analysis to make use of *in vivo* data which might be very different from *in vitro* enzyme kinetics as has been pointed out for the ethylmalonyl-CoA pathway in this study. In this context, modelling approaches that are able to quantify uncertainties and identify missing data are ideal. This way, models and simulations can be iteratively improved to get closer and closer to the biological reality and a deeper understanding of metabolism and design principles of natural pathways.

## Data Availability

The kinetic and thermodynamic data of all enzymes and reactions used to support the findings of this study are included within the supplementary information file "Reactions_Composite22_model.tsv" and in the supplementary information document. The MATLAB code that was used to calculate pathway activities is deposited at https://github.com/HannesLoewe/pathway-comparison-ECM and can be reused under the GNU General Public License.

## Supplementary Materials

Supplementary Materials The kinetic and thermodynamic data of all enzymes and reaction used to support the findings of this study are included within the supplementary information file "Reactions_Composite22_model.tsv" and in the supplementary information document.
