# Corrigendum to “In-Depth Computational Analysis of Natural and Artificial Carbon Fixation Pathways”

**DOI:** 10.34133/2021/9756012

**Published:** 2021-11-16

**Authors:** Hannes Löwe, Andreas Kremling

**Affiliations:** Systems Biotechnology, Technical University of Munich, Germany

In the article titled “In-Depth Computational Analysis of Natural and Artificial Carbon Fixation Pathways” [1], there were errors in Figures 7–10.

In the label of Figure 9, it reads: “Glyceraldehyde-3-phosphate was chosen as a product for the pathways,” but this is corrected to “Pyruvate was chosen as a product and formate as a substrate for the pathways.”

In the lower panels of Figures 7, 8, and 10, the labels “MOG/rCCC” and “CETCH cycle” in the figure key should be inverted. Also, in these figures, the panel indicators (a) and (b) were missing.

**Figure 7:**
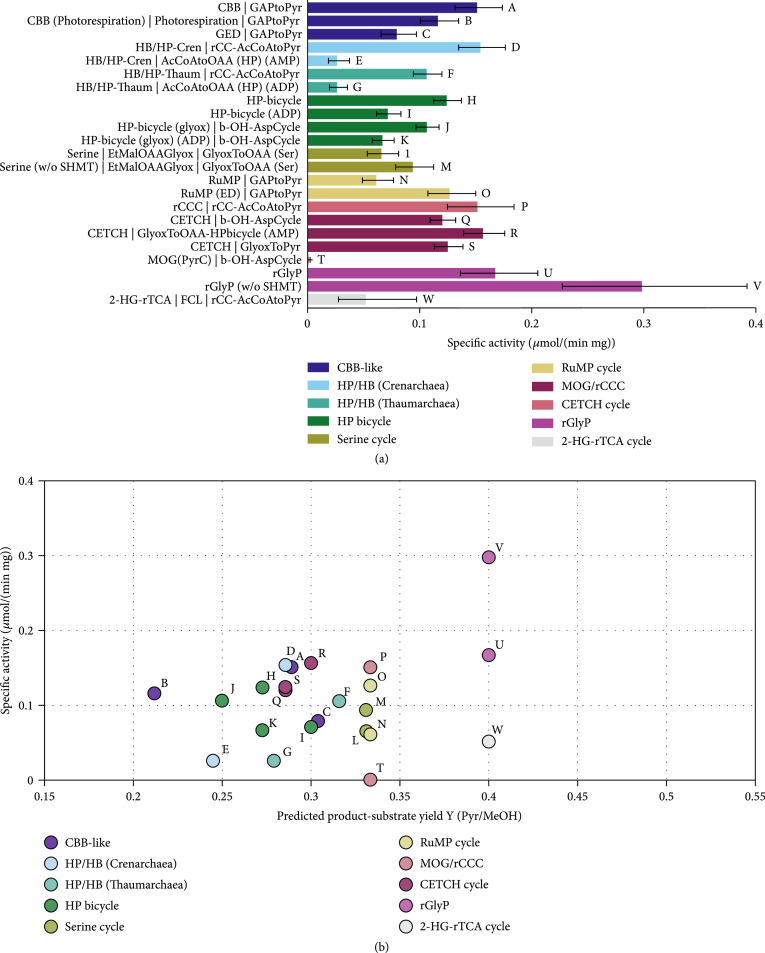
Comparison of carbon fixation pathways using methanol as a substrate for the production of pyruvate with the Enzyme Cost Minimization algorithm. “(w/o SHMT)” indicates that the costs of the serine hydroxymethyltransferase are not included in the respective pathway’s activity. The concentration of CO_2_ was assumed to be 1 mM and the concentration of HCO_3_^-^ to be 10 mM. (a) Pathway-specific activities with standard deviations and the full name of the main pathways and the connecting modules to transform their primary product to pyruvate. Pathway abbreviations: CBB(PTS): phosphatase-free CBB cycle; HP-bicycle(glyox): glyoxylate producing subcycle of the 3-HP bicycle; GAPtoPyr: glycolysis; FCL: formaldehyde:NADP+ oxidoreductase (formyl-CoA-forming); GlyoxToPyr: GCL/GDH route; b-OH-AspCycle: *β*-hydroxyaspartate cycle; AcCoAtoOAA(HP): acetyl-CoA to oxaloacetate-converting module derived from the 3-HP/4-HB cycle using either ADP- or AMP-producing CoA-ester synthases; rCC-AcCoAtoPyr: acetyl-CoA to pyruvate-converting module derived from the rCCC; GlyoxToOAA: glyoxylate- to oxaloacetate-converting module based on the serine cycle. (b) Pathway-specific activities compared to product-substrate yield. The overall stoichiometries of all pathways and modules are listed in Table 1 and supplementary Tables S4 and S5. The small letters label each pathway and correspond to the labels and the respective pathway combinations in (a).

**Figure 8:**
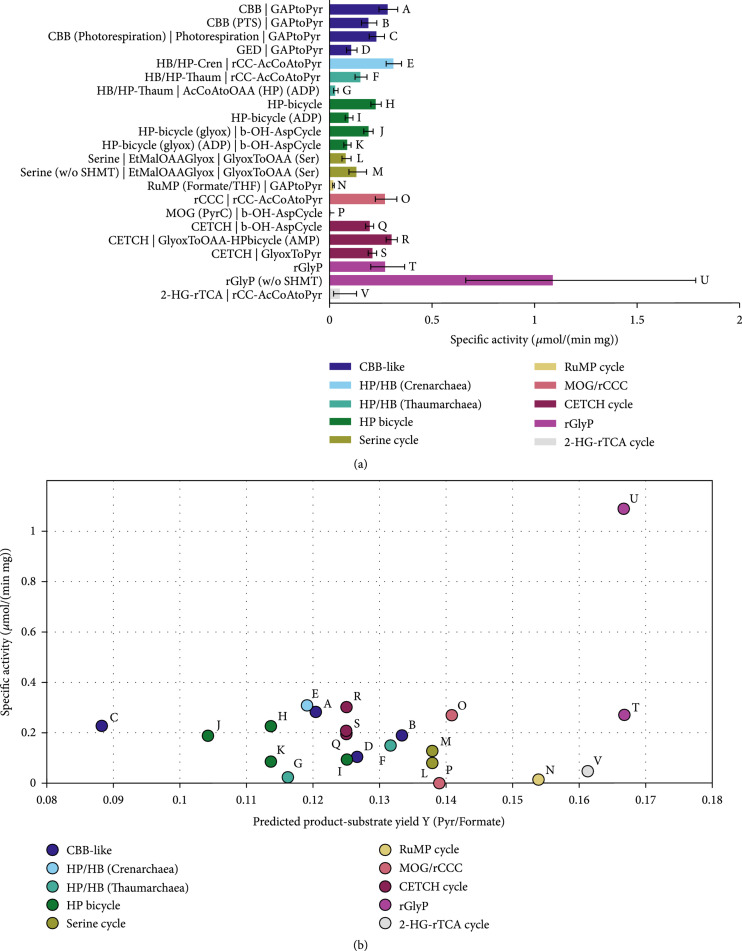
Comparison carbon fixation pathways using formate as a substrate for the production of pyruvate with the Enzyme Cost Minimization algorithm. “(w/o SHMT)” indicates that the cost of the serine hydroxymethyltransferase is not included in the respective pathway’s activity. The concentration of CO_2_ was assumed to be 1 mM and the concentration of HCO_3_^-^ to be 10 mM. (a) Pathway-specific activities with standard deviations and the full name of the main pathways and the connecting modules to transform their primary product to pyruvate. Pathway abbreviations: CBB(PTS): phosphatase-free CBB cycle; HP-bicycle(glyox): glyoxylate producing subcycle of the 3-HP bicycle; GAPtoPyr: glycolysis; FCL: formaldehyde:NADP+ oxidoreductase (formyl-CoA-forming); GlyoxToPyr: GCL/GDH route; b-OH-AspCycle: *β*-hydroxyaspartate cycle; AcCoAtoOAA(HP): acetyl-CoA- to oxaloacetate-converting module derived from the 3-HP/4-HB cycle using either ADP- or AMP-producing CoA-ester synthases; rCC-AcCoAtoPyr: acetyl-CoA- to pyruvate-converting module derived from the rCCC; GlyoxToOAA: glyoxylate to oxaloacetate converting module based on the serine cycle. (b) Pathway-specific activities compared to product-substrate yield. The overall stoichiometries of all pathways and modules are listed in Table 1 and supplementary Tables S4 and S5. The small letters label each pathway and correspond to the labels and the respective pathway combinations in (a).

**Figure 9:**
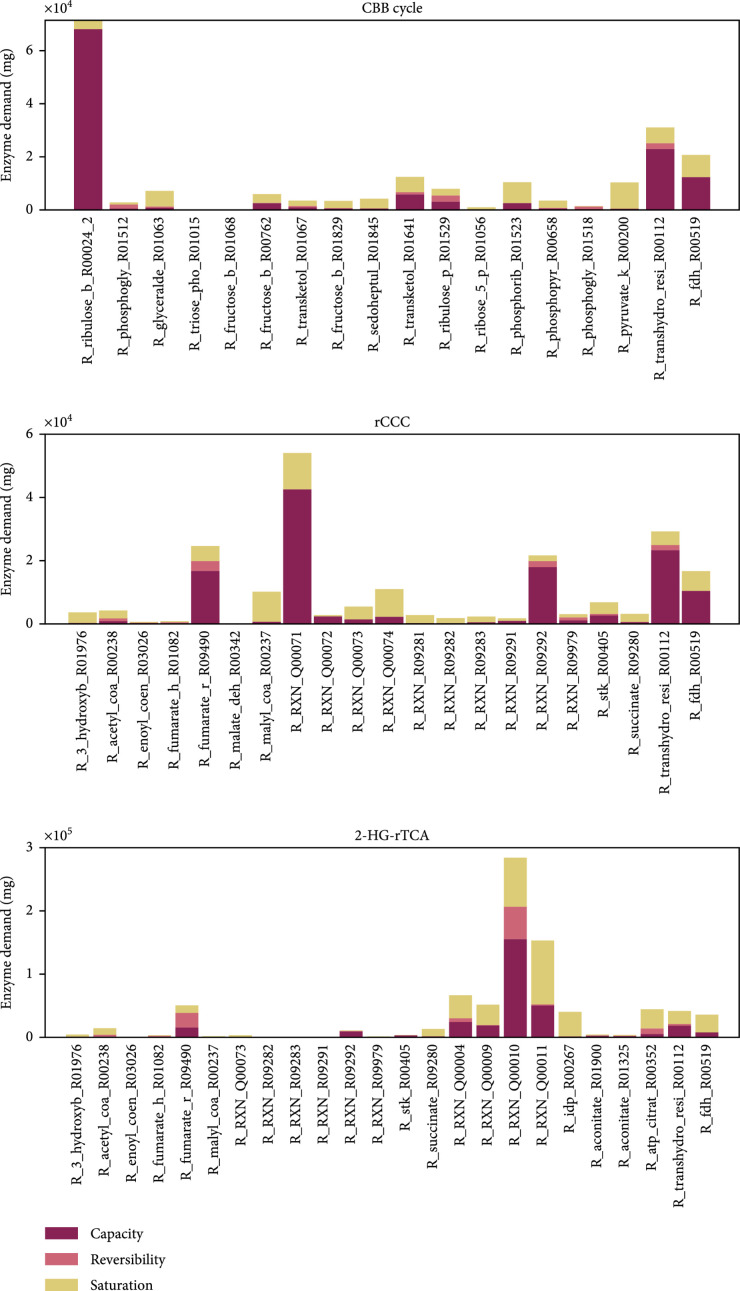
Enzyme demands to sustain a total pathway activity of 1 mmol product per second. Contribution of the capacity, the reversibility, and the saturation with substrates or products of each reaction to the demand of enzymes for the CBB cycle, the rCCC, and the 2-HG-rTCA cycle. The figure follows the wording of Noor et al. [29]. “Capacity”: demand of enzyme caused by a limitation by the catalytic rate constant; “Reversibility”: extra amount of enzyme needed because of a backward flux; “Saturation”: additional enzyme necessary because of undersaturation with a substrate or oversaturation with a product. The values present the optimized state as predicted by the ECM algorithm assuming a CO_2_ concentration of 10 *μ*M and HCO_3_^-^ concentration of 100 *μ*M. Pyruvate was chosen as a product and formate as a substrate for the pathways. Reaction names correspond to their identifiers in the SBtab model file (Supplementary file Reactions_Composite22_model.tsv (available here)).

**Figure 10:**
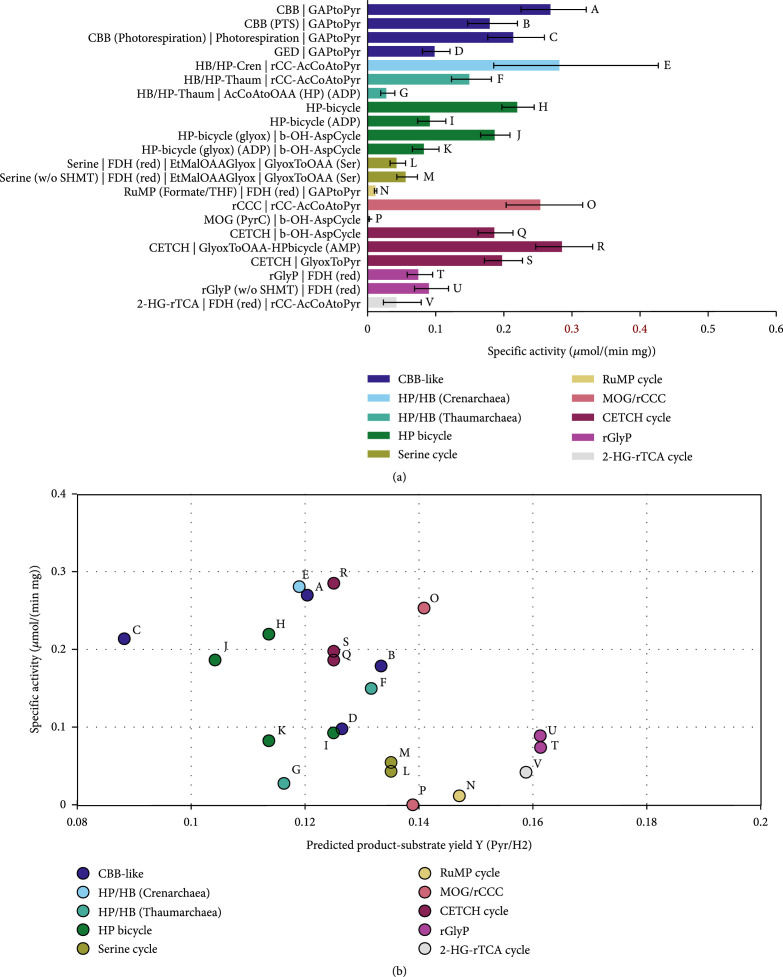
Comparison carbon fixation pathways using H_2_/CO_2_ as a substrate for the production of pyruvate with the Enzyme Cost Minimization algorithm. “(w/o SHMT)” indicates that the cost of the serine hydroxymethyltransferase is not included in the respective pathway’s activity. The concentration of CO_2_ was assumed to be 1 mM and the concentration of HCO_3_^-^ to be 10 mM. (a) Pathway-specific activities with standard deviations and the full name of the main pathways and the connecting modules to transform their primary product to pyruvate. Pathway abbreviations: CBB(PTS): phosphatase-free CBB cycle; HP-bicycle(glyox): glyoxylate-producing subcycle of the 3-HP bicycle; GAPtoPyr: glycolysis; FCL: formaldehyde:NADP+ oxidoreductase (formyl-CoA-forming); GlyoxToPyr: GCL/GDH route; b-OH-AspCycle: *β*-hydroxyaspartate cycle; AcCoAtoOAA(HP): acetyl-CoA- to oxaloacetate-converting module derived from the 3-HP/4-HB cycle using either ADP- or AMP-producing CoA-ester synthases; rCC-AcCoAtoPyr: acetyl-CoA- to pyruvate-converting module derived from the rCCC; GlyoxToOAA: glyoxylate- to oxaloacetate-converting module based on the serine cycle. (b) Pathway-specific activities compared to product-substrate yield. The overall stoichiometries of all pathways and modules are listed in Table 1 and supplementary Tables S4 and S5. The small letters label each pathway and correspond to the labels and the respective pathway combinations in (a).
