# Supplementary material for: In-Depth Computational Analysis of Natural and Artificial Carbon Fixation Pathways
Source: Biodes Res. 2021 Aug 31;2021:9898316. doi: 10.34133/2021/9898316 (PMC10521678; doi:10.34133/2021/9898316)
Supplement: Supplementary Materials — The kinetic and thermodynamic data of all enzymes and reaction used to support the findings of this study are included within the supplementary information file "Reactions_Composite22_model.tsv" and in the supplementary information document. [file 9898316.f1.zip › Supplementary Information - Revised3.pdf]

Supplementary Information for

In-depth computational analysis of natural and artificial carbon fixation pathways

Hannes Löwe* [a] and Andreas Kremling [a]

[a] Systems Biotechnology, Technical University of Munich

* Corresponding author

1. Detailed pathway maps of all pathways with respective enzymes

1.1. Artificial and natural CO2/C1 fixing pathways

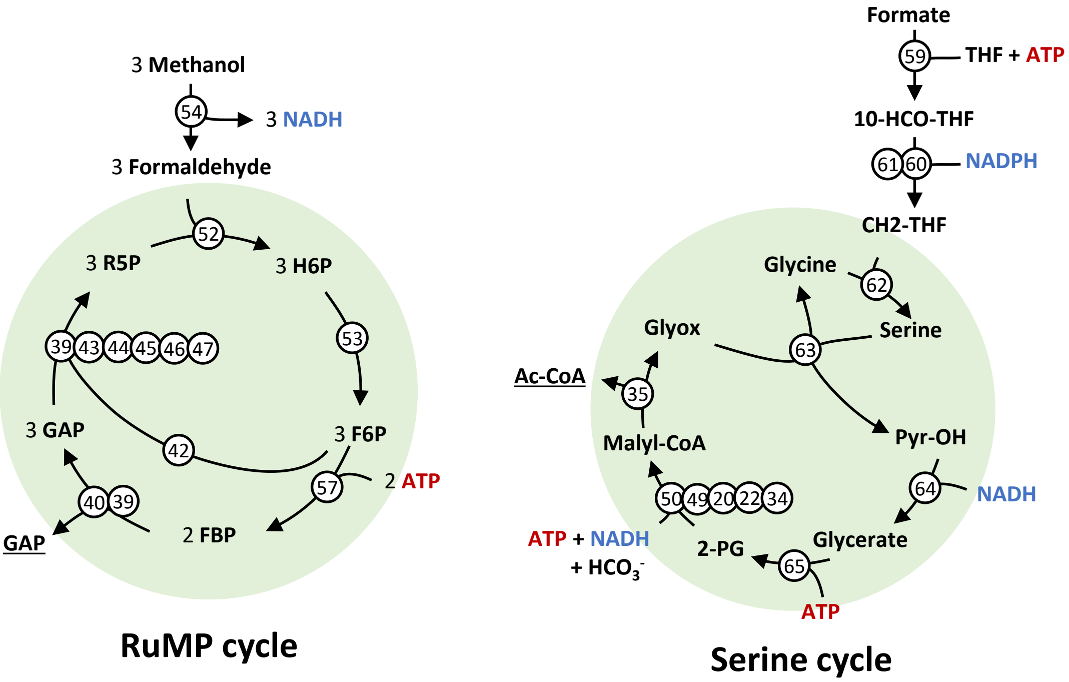

Figure S1: Flux maps of natural C1-fixing pathways, in this case methanol (RuMP-Cycle) and formate (Serine-Cycle). For simplification, reaction arrows can include multiple reactions and skip some metabolites. ADP, AMP, phosphate, water, and oxidized forms of electron carriers were also left out to improve clarity. Reactions are numbered and each number specifies the respective enzyme according to Table S1. Abbreviations: Ac-CoA, acetyl-CoA; Glyox, glyoxylate; GAP, glyceraldehyde-3-phosphate; R5P, ribulose-5-phosphate; H6P, hexulose-6-phosphate; OH-Pyr, hydroxypyruvate; 2-PG, 2-phosphoglycerate; 10-CHO-THF, 10-formyltetrahydrofolate; CH2-THF, 5,10-methylenetetrahydrofolate; LP-S_2, [glycine-cleavage complex H protein]-N6-lipoyl-L-lysine; LP-S-CH_2NH_2, [glycine-cleavage complex H protein]-S-aminomethyl-N6-dihydrolipoyl-L-lysine; LP-SH, [glycine-cleavage complex H protein]- dihydrolipoyl-L-lysine.

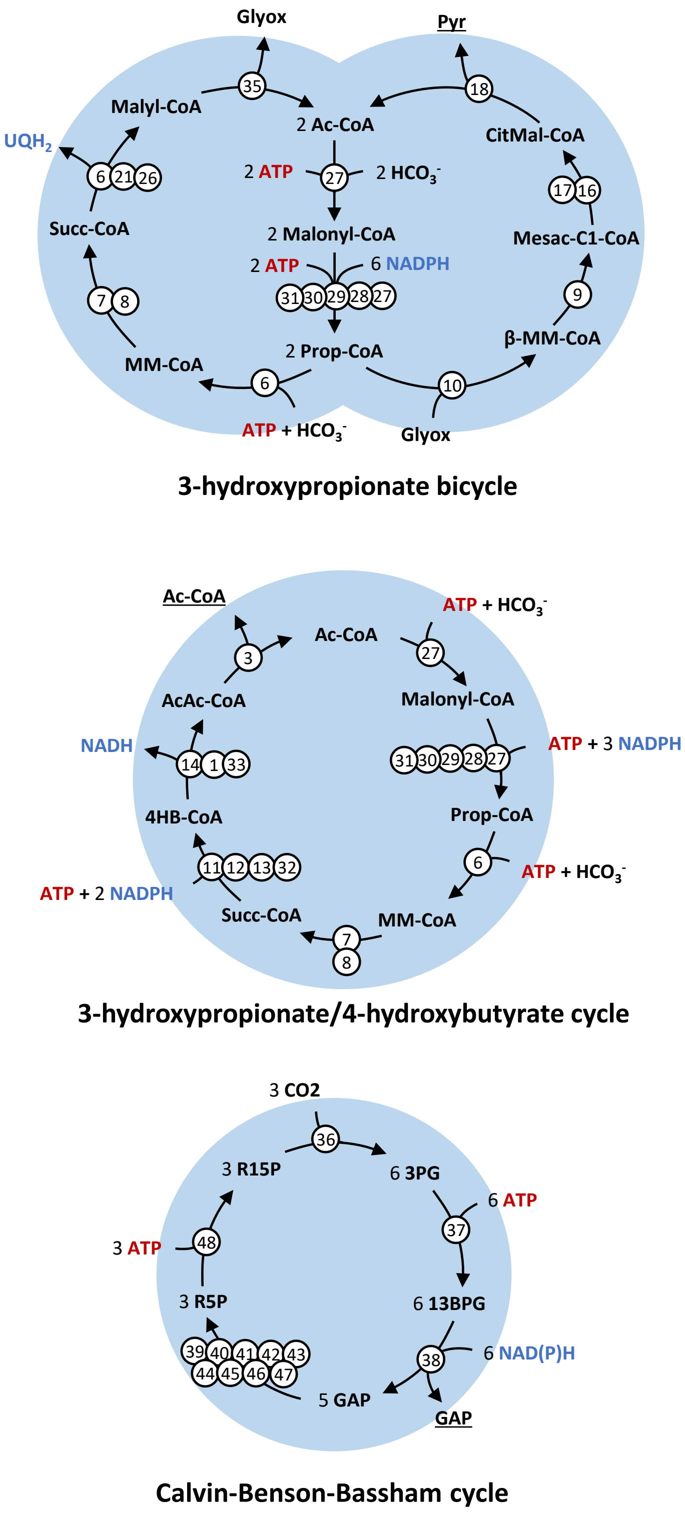

Figure S2: Flux maps of natural CO_2-fixing pathways. For simplification, reaction arrows can include multiple reactions and skip some metabolites. ADP, AMP, phosphate, water, and oxidized forms of electron carriers were also left out to improve clarity. Reactions are numbered and each number specifies the respective enzyme according to Table S1. Abbreviations: Ac-CoA, acetyl-CoA; Prop-CoA, propanoyl-CoA; β-MM-CoA, β-methylmalyl-CoA; CitMal-CoA, citramalyl-CoA; Pyr, pyruvate; MM-CoA, methylmalonyl-CoA; Succ-CoA, succinyl-CoA; Glyox, glyoxylate; 4HB-CoA, 4-hydroxybutyrate; AcAc-CoA, acetoacetyl-CoA; R15P, ribulose-1,5-bisphosphate; 3PG, 3-phosphoglycerate; 13BPG, 1,3-bisphophoglycerate; GAP, glyceraldehyde-3-phosphate; R5P, ribulose-5-phosphate.

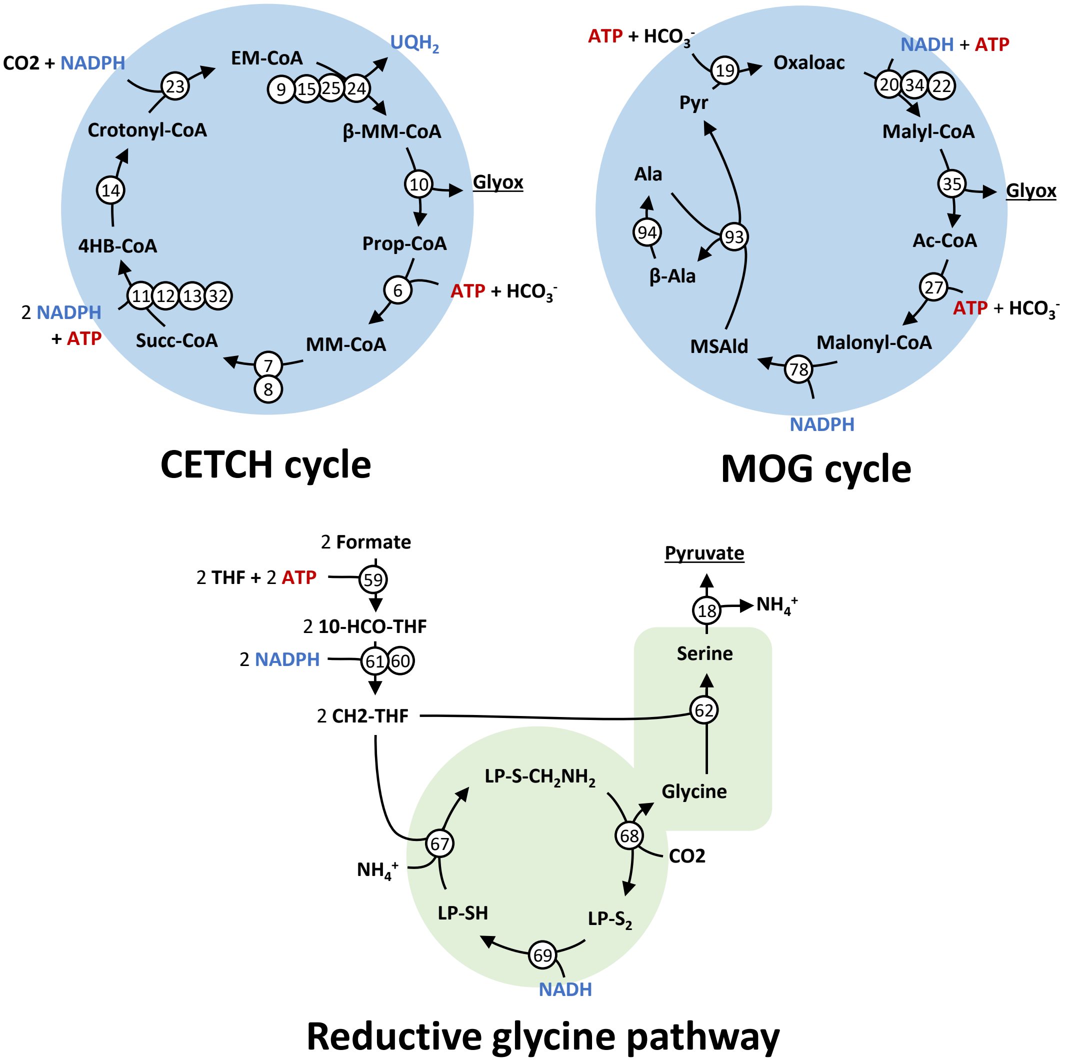

Figure S3: Flux maps of alternative CO_2- or C1-fixing pathways established or proposed in earlier works. For simplification, reaction arrows can include multiple reactions and skip some metabolites. ADP, AMP, phosphate, water, and oxidized forms of electron carriers were also left out to improve clarity. Reactions are numbered and each number specifies the respective enzyme according to Table S1. Abbreviations: Ac-CoA, acetyl-CoA; Prop-CoA, propanoyl-CoA; β-MM-CoA, β-methylmalyl-CoA; Pyr, pyruvate; MM-CoA, methylmalonyl-CoA; Succ-CoA, succinyl-CoA; Glyox, glyoxylate; 4HB-CoA, 4-hydroxybutyrate; OH-Pyr, hydroxypyruvate; MSAld: malonate semialdehyde; Ala: alanine; β-Ala: β-alanine; Oxaloac: oxaloacetate; Oxaloac: oxaloacetate; Ac-CoA: acetyl-CoA; 10-CHO-THF, 10-formyltetrahydrofolate; CH2-THF, 5,10-methylenetetrahydrofolate; LP-S_2, [glycine-cleavage complex H protein]-N6-lipoyl-L-lysine; LP-S-CH_2NH_2, [glycine-cleavage complex H protein]-S-aminomethyl-N6-dihydrolipoyl-L-lysine; LP-SH, [glycine-cleavage complex H protein]- dihydrolipoyl-L-lysine.

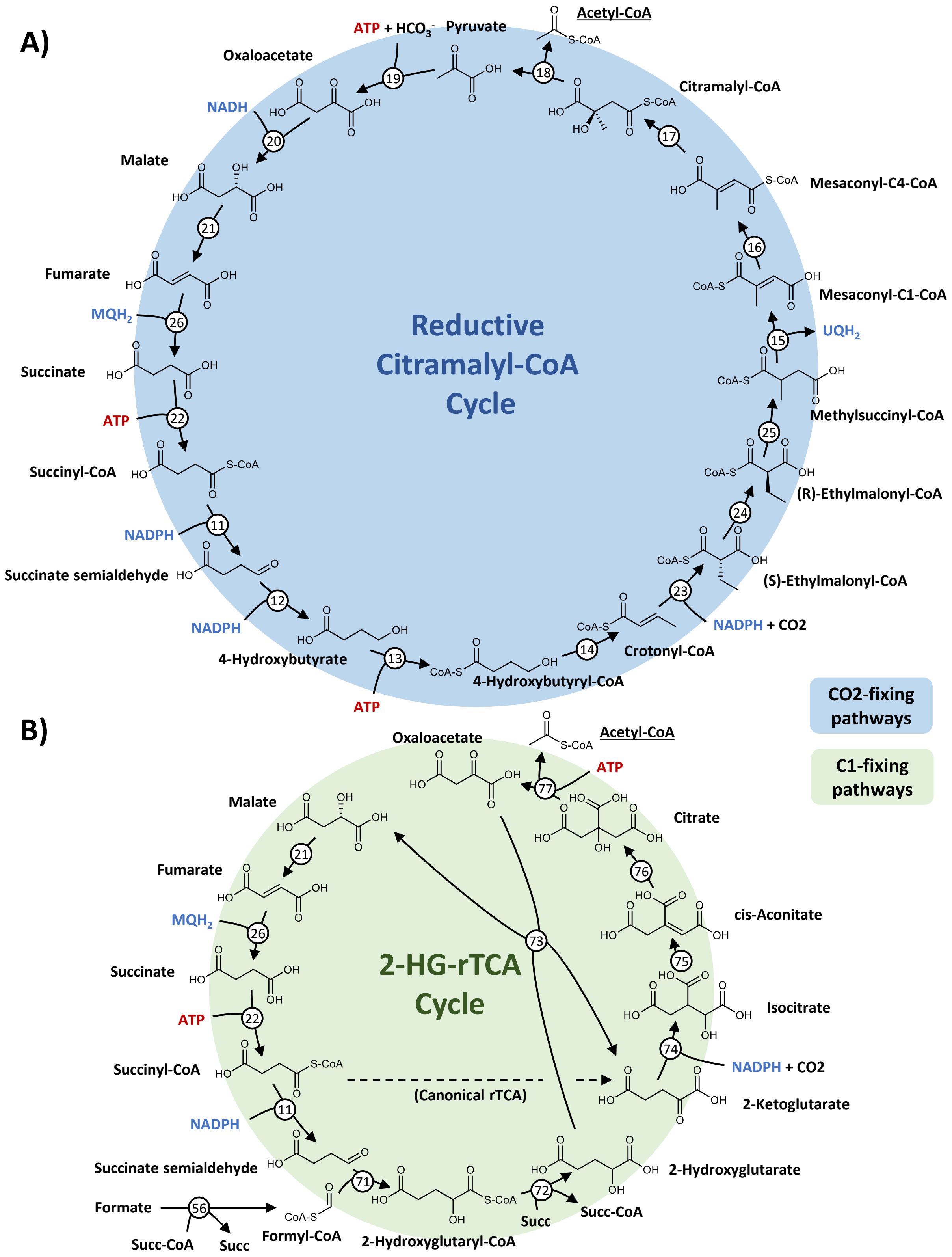

Figure S4: Flux maps of the rCCC and 2-HG-rTCA Cycle. ADP, AMP, phosphate, water, and oxidized forms of electron carriers were also left out to improve clarity. Reactions are numbered and each number specifies the respective enzyme according to Table S1. Abbreviations: Succ, succinate; Succ-CoA, succinyl-CoA.

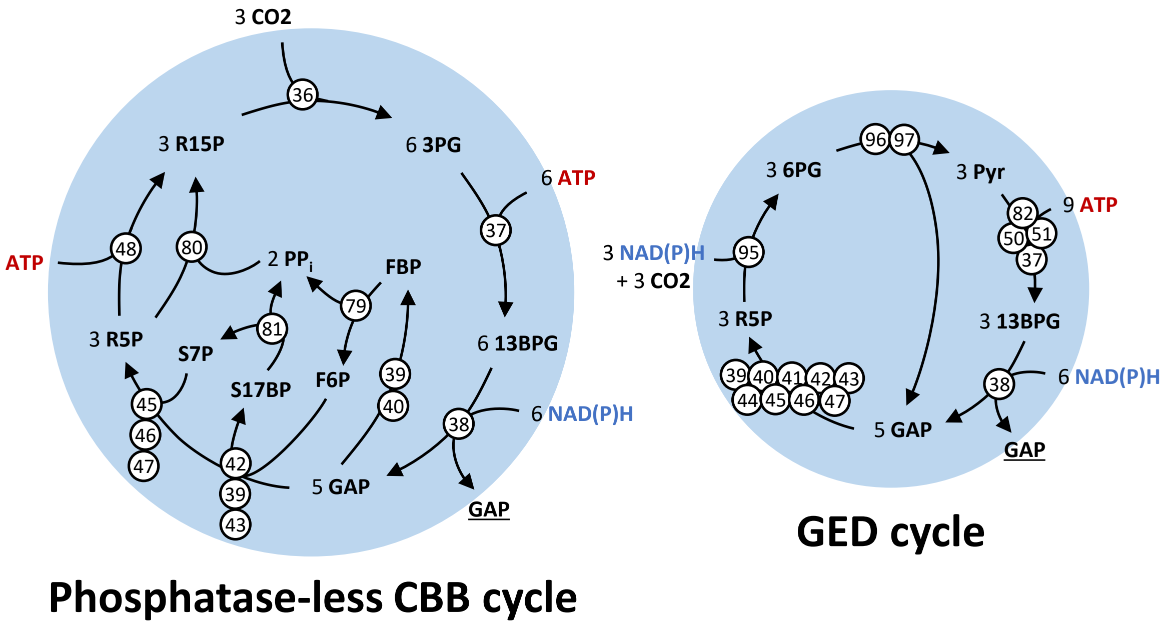

Figure S5: Flux maps of the hypothetical phosphatase-less Calvin-Benson-Bassham Cycle and the GED Cycle. For simplification, reaction arrows can include multiple reactions and skip some metabolites. ADP, AMP, phosphate, water, and oxidized forms of electron carriers were also left out to improve clarity. Reactions are numbered and each number specifies the respective enzyme according to Table S1. Abbreviations: R15P, ribulose-1,5-bisphosphate; 3PG, 3-phosphoglycerate; 13BPG, 1,3-bisphosphoglycerate; GAP, glyceraldehyde-3-phosphate; FBP, fructose-1,6-bisphosphate; F6P, fructose-6-phosphate; S17BP, seduheptulose-1,7-bisphosphate; PPi, pyrophyosphate; S7P, seduheptulose-7-phosphate; R5P, ribulose-5-phosphate; 6PG: 6-phosphogluconate; Pyr: pyruvate.

1.2. Pathway modules connecting central metabolites

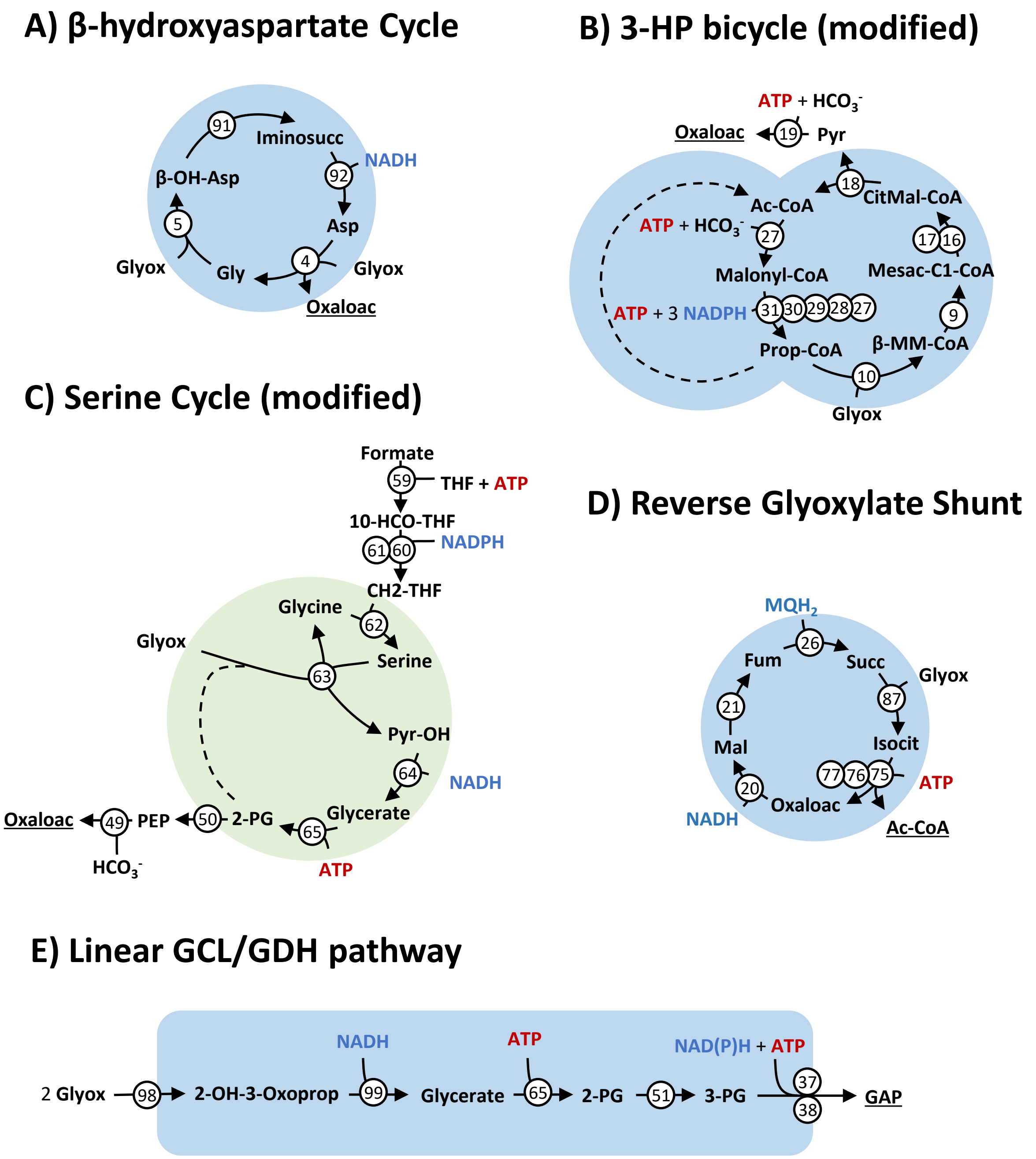

Figure S6: Flux maps of pathway subnetworks that are modules for conversion of **glyoxylate to acetyl-CoA or oxaloacetate**. For simplification, reaction arrows can include multiple reactions and skip some metabolites. ADP, AMP, phosphate, water, and oxidized forms of electron carriers were also left out to improve clarity. Reactions are numbered and each number specifies the respective enzyme according to Table S1. Abbreviations: Iminosucc, iminosuccinate; β-OH-Asp, β-hydroxyaspartate; Asp, aspartate; Glyox, glyoxylate; Oxaloac, oxaloacetate; Gly, glycine; Pyr, pyruvate; CitMal-CoA, citramalyl-CoA; Ac-CoA, acetyl-CoA; Prop-CoA, propanoyl-CoA; β-MM-CoA, β-methylmalyl-CoA; Mesac-C1-CoA, mesaconyl-C1-CoA; Fum, fumarate; Succ, succinate; Isocit, isocitrate; Mal, malate; PEP, phosphoenolpyruvate; OH-Pyr, hydroxypyruvate; 2-PG, 2-phosphoglycerate; 10-CHO-THF, 10-formyltetrahydrofolate; CH2-THF, 5,10-methylenetetrahydrofolate; LP-S_2, [glycine-cleavage complex H protein]-N6-lipoyl-L-lysine; LP-S-CH_2NH_2, [glycine-cleavage complex H protein]-S-aminomethyl-N6-dihydrolipoyl-L-lysine; LP-SH, [glycine-cleavage complex H protein]- dihydrolipoyl-L-lysine.

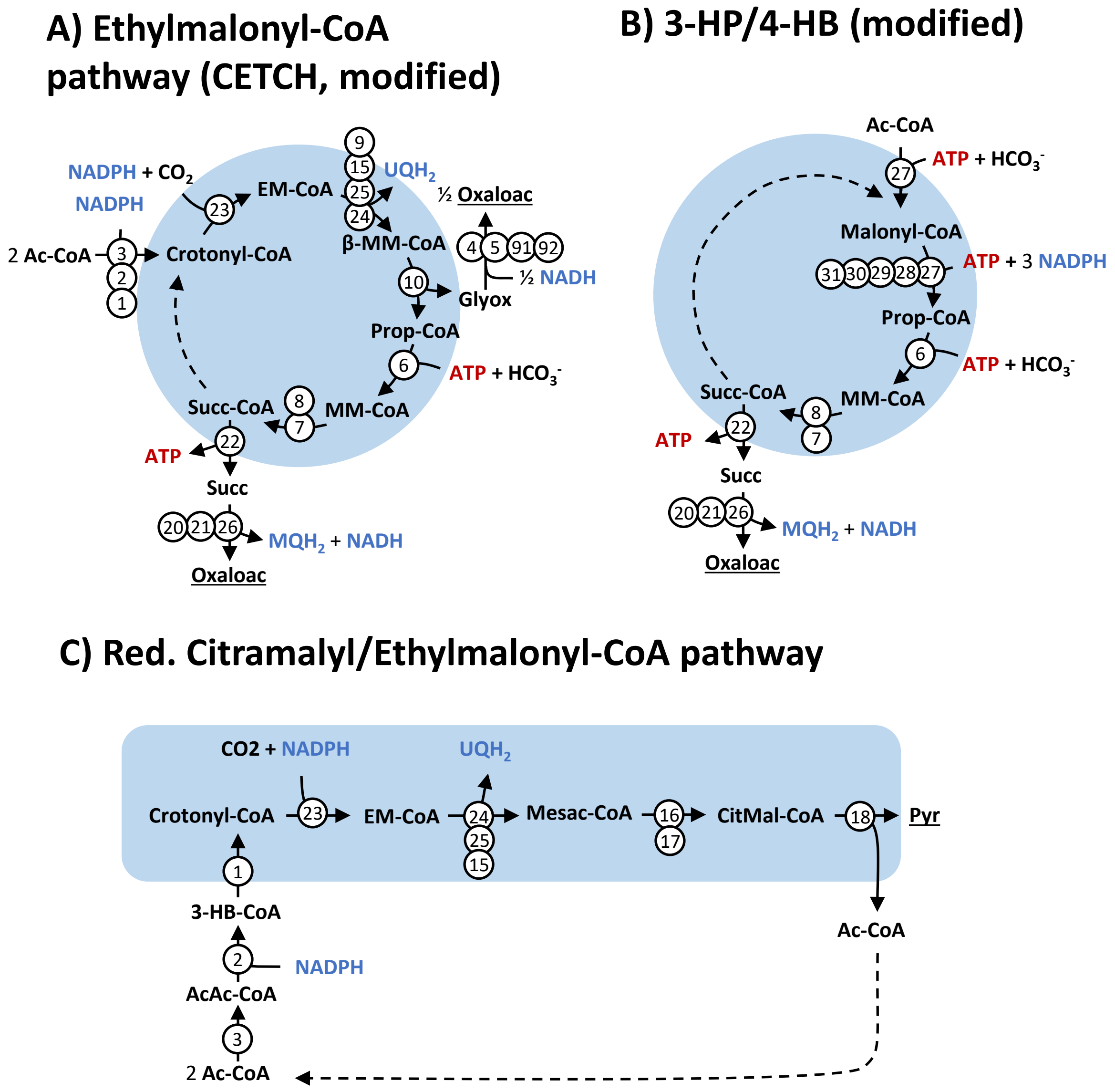

Figure S7: Flux maps of pathway subnetworks that are modules for conversion of **acetyl-CoA to pyruvate or oxaloacetate**. For simplification, reaction arrows can include multiple reactions and skip some metabolites. ADP, AMP, phosphate, water, and oxidized forms of electron carriers were also left out to improve clarity. Reactions are numbered and each number specifies the respective enzyme according to Table S1. Abbreviations: Glyox, glyoxylate; Oxaloac, oxaloacetate; Pyr, pyruvate; EM-CoA, ethylmalonyl-CoA; CitMal-CoA, citramalyl-CoA; Ac-CoA, acetyl-CoA; Prop-CoA, propanoyl-CoA; β-MM-CoA, β-methylmalyl-CoA; MM-CoA, methylmalonyl-CoA; Succ-CoA, succinyl-CoA; Mesac-CoA, mesaconyl-CoA; Succ, succinate; AcAc-CoA, acetoacetyl-CoA; 3-HB-CoA, 3-hydroxybutanoyl-CoA; 10-CHO-THF, 10-formyltetrahydrofolate; CH2-THF, 5,10-methylenetetrahydrofolate; LP-S_2, [glycine-cleavage complex H protein]-N6-lipoyl-L-lysine; LP-S-CH_2NH_2, [glycine-cleavage complex H protein]-S-aminomethyl-N6-dihydrolipoyl-L-lysine; LP-SH, [glycine-cleavage complex H protein]-dihydrolipoyl-L-lysine.

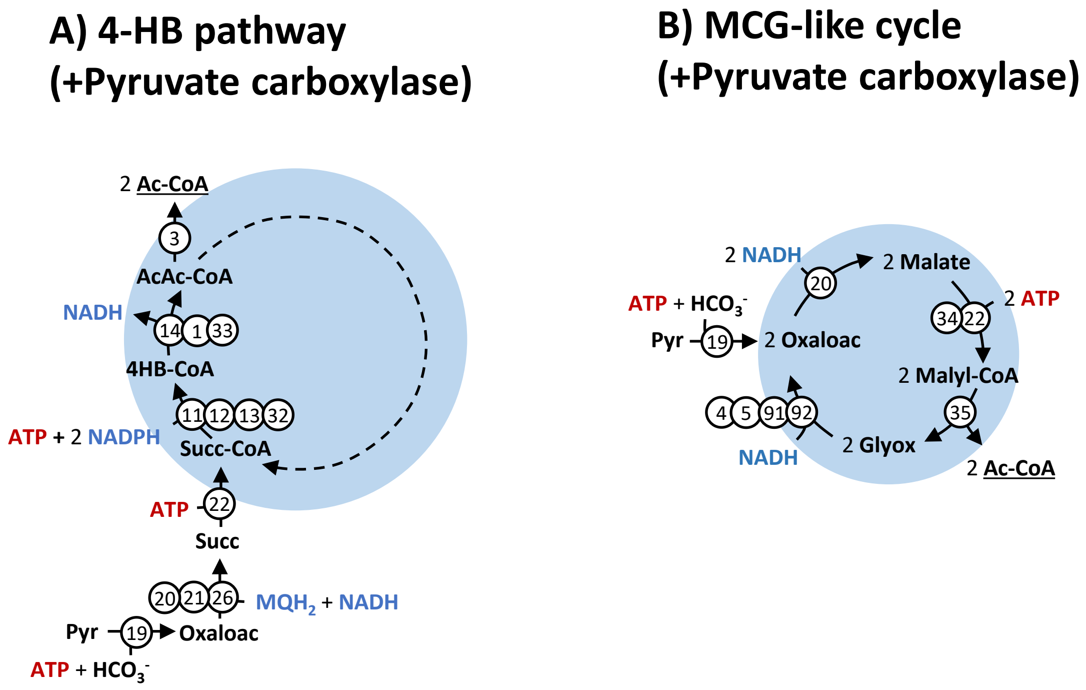

Figure S8: Flux maps of pathway subnetworks that are modules for conversion of **one mol pyruvate or oxaloacetate to 2 mol acetyl-CoA**. For simplification, reaction arrows can include multiple reactions and skip some metabolites. ADP, AMP, phosphate, water, and oxidized forms of electron carriers were also left out to improve clarity. Reactions are numbered and each number specifies the respective enzyme according to Table S1. Abbreviations: Glyox, glyoxylate; Oxaloac, oxaloacetate; Pyr, pyruvate; Ac-CoA, acetyl-CoA; Prop-CoA, propanoyl-CoA; Succ-CoA, succinyl-CoA; Malate, malate; Mesac-CoA, mesaconyl-CoA; Succ, succinate; AcAc-CoA, acetoacetyl-CoA; 4HB-CoA, 4-hydroxybutanoyl-CoA.

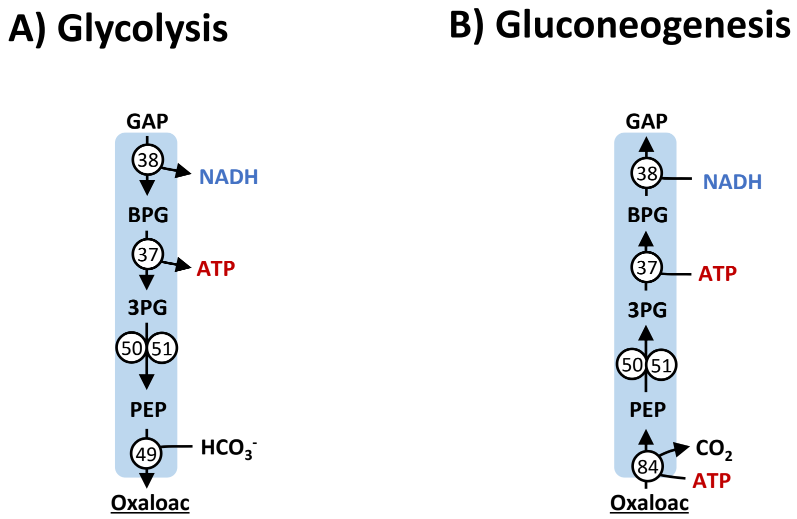

Figure S9: Flux maps of pathway subnetworks that are modules for conversion **of glyceraldehyde-3-phosphate to oxaloacetate (part of the glycolysis) and vice versa (gluconeogenesis)**. For simplification, reaction arrows can include multiple reactions and skip some metabolites. ADP, AMP, phosphate, water, and oxidized forms of electron carriers were also left out to improve clarity. Reactions are numbered and each number specifies the respective enzyme according to Table S1. Abbreviations: GAP, glyceraldehyde-3-phosphate; BPG, 1,3-bisphosphoglycerate; 3PG, 3-phosphoglycerate; PEP, phosphoenolpyruvate; Oxaloac, oxaloacetate.

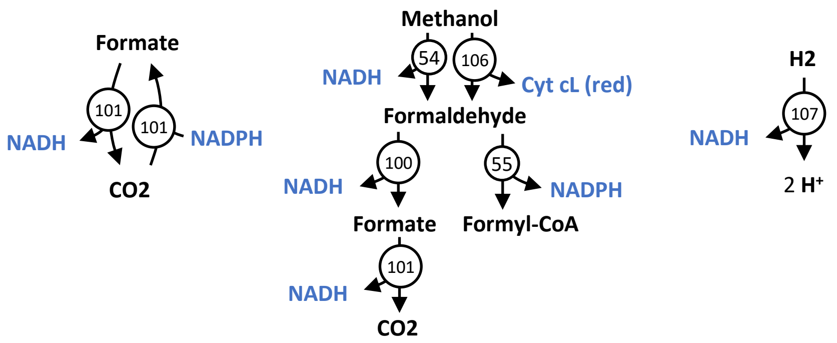

Figure S10: Electron recovery with Formate, Methanol and H_2 and generation of Formate, Formaldehyde and Formyl-CoA as substrates for C1-using metabolic pathways. For simplification, reaction arrows can include multiple reactions and skip some metabolites.

2. Data and parameters used in this study with references

Table S1: Data sources for kinetic data used in this work

Database/source	Extracted data
KEGG (Kanehisa & Goto, 2000)	• Reaction stoichiometries<br>• Systematic names of enzymes
eQuilibrator (Flamholz et al., 2012)	• Equilibrium constants and Gibbs free energies under standard conditions $\Delta_r G^0$ and respective uncertainties (pH 7.5, Ionic strength of 0.25 M)<br>• Templates for SBtab-files using the ECM pathway analysis platform
BRENDA (Jeske et al., 2019)	• Starting point for kinetic data search, using the primary sources linked with kinetic data<br>• Direct source of kinetic data when no data was available for a certain enzyme
UniProt (Bateman, 2019)	• Protein molecular weights if not specified in primary source
Primary literature focused on enzyme characterization*	• Main source for enzymatic parameters K_m, k_{cat}^+ and k_{cat}^-<br>• Molecular weight of enzymes if available

* details are listed in supplementary Table S2

2.1. Kinetic parameters of enzymes

Table S2: Kinetic parameters used in this study with their uncertainty and sources

Enzyme name/ Parameter	Value	Uncer-tainty	Unit	Comment	Source	#
**(3S)-3-hydroxyacyl-CoA hydro-lyase**						**1**
Stoichiometry: (S)-3-hydroxybutanoyl-CoA(aq) ⇌ crotonoyl-CoA(aq) + H2O(l)						
K_{eq}	0.2155				eQuilibrator	
M	15355		g mol^{-1}		UniProt	
Backward reaction:						
K_m (crotonyl-CoA)	9.26		µM		(Moskowitz & Merrick, 1969)	
K_{cat}^-	49.14		s^{-1}		(Moskowitz & Merrick, 1969)	
**(S)-3-hydroxyacyl-CoA:NADP+ oxidoreductase**						**2**
Stoichiometry: (S)-3-hydroxybutanoyl-CoA(aq) + NADP+(aq) ⇌ acetoacetyl-CoA(aq) + NADPH(aq)						
K_{eq}	$2.814 \cdot 10^{-3}$				eQuilibrator	
M	141000		g mol^{-1}		(Belova et al., 1997)	
Backward reaction:						
K_m (acetoacetyl-CoA)	11.6		µM		(Belova et al., 1997)	
K_m (NADPH)	41		µM		(Belova et al., 1997)	
K_{cat}^-	432.4		s^{-1}		(Belova et al., 1997)	
**acetyl-CoA:acetyl-CoA C-acetyltransferase**						**3**
Stoichiometry: 2 acetyl-CoA(aq) ⇌ CoA(aq) + acetoacetyl-CoA(aq)						
K_{eq}	$2.77 \cdot 10^{-5}$				eQuilibrator	
M	86497		g mol^{-1}		UniProt	
Forward reaction:						
K_m (acetyl-CoA)	600		µM		(Hedl et al., 2002)	
K_{cat}^+	122.5		s^{-1}		(Hedl et al., 2002)	
Backward reaction:						
K_m (acetoacetyl-CoA)	88		µM		(Hedl et al., 2002)	
K_m (CoA)	10		µM		(Hedl et al., 2002)	
K_{cat}^-	1802		s^{-1}		(Hedl et al., 2002)	
**L-aspartate:glyoxylate aminotransferase**						**4**
Stoichiometry: aspartate(aq) + glyoxylate(aq) ⇌ oxaloacetate(aq) + glycine(aq)						
K_{eq}	9.546				eQuilibrator	
M	42507		g mol^{-1}		(Schada von Borzyskowski et al., 2019)	
Forward reaction:						
K_m (aspartate)	2500	100	µM		(Schada von Borzyskowski et al., 2019)	
K_m (glyoxylate)	430	20	µM		(Schada von Borzyskowski et al., 2019)	
K_{cat}^+	57	1	s^{-1}		(Schada von Borzyskowski et al., 2019)	
Backward reaction:						
K_m (oxaloacetate)	2900	270	µM		(Schada von Borzyskowski et al., 2019)	
K_m (glycine)	9500	400	µM		(Schada von Borzyskowski et al., 2019)	
K_{cat}^-	0.76	0.02	s^{-1}		(Schada von Borzyskowski et al., 2019)	
**(2R,3S)-beta-Hydroxyasparate glyoxylate-lyase (glycine-forming)**						**5**
Stoichiometry: 3-hydroxyaspartate(aq) ⇌ glycine(aq) + glyoxylate(aq)						
K_{eq}	0.536				eQuilibrator	
M	41786		g mol^{-1}		(Schada von Borzyskowski et al., 2019)	
Forward reaction:						
K_m (3-hydroxyaspartate)	280	30	µM		(Schada von Borzyskowski et al., 2019)	
K_{cat}^+	33	1	s^{-1}		(Schada von Borzyskowski et al., 2019)	
Backward reaction:						
K_m (glyoxylate)	230	30	µM		(Schada von Borzyskowski et al., 2019)	
K_m (glycine)	4310	340	µM		(Schada von Borzyskowski et al., 2019)	
K_{cat}^-	89	4	s^{-1}		(Schada von Borzyskowski et al., 2019)	
**propanoyl-CoA:carbon-dioxide ligase (ADP-forming)**						**6**
Stoichiometry: ATP(aq) + propanoyl-CoA(aq) + HCO3-(aq) ⇌ ADP(aq) + phosphate(aq) + (S)-methylmalonyl-CoA(aq)						
K_{eq}	16.20				eQuilibrator	
M	510000		g mol^{-1}		(Kimura et al., 1998)	
Forward reaction:						
K_m (ATP)	36		µM		(Kimura et al., 1998)	
K_m (propanoyl-CoA)	32		µM		(Kimura et al., 1998)	
K_m (HCO_3^-)	1130		µM		(Kimura et al., 1998)	
K_{cat}^+	120.1		s^{-1}		(Kimura et al., 1998)	

Table S2 (continued)

Enzyme name/ Parameter	Value	Uncer-tainty	Unit	Comment	Source	#
**methylmalonyl-CoA epimerase**						**7**
Stoichiometry: (R)-methylmalonyl-CoA(aq) ⇌ (S)-methylmalonyl-CoA(aq)						
K_{eq}	1				eQuilibrator	
M	16081		g mol^{-1}		(Dayem et al., 2002)	
Forward reaction:						
K_m ((R)-methylmalonyl-CoA)	38		µM		(Dayem et al., 2002)	
K_{cat}^+	150		s^{-1}		(Dayem et al., 2002)	
**(R)-methylmalonyl-CoA CoA-carbonylmutase**						**8**
Stoichiometry: (R)-methylmalonyl-CoA(aq) ⇌ succinyl-CoA(aq)						
K_{eq}	22.23				eQuilibrator	
M	141774		g mol^{-1}		UniProt	
Forward reaction:						
K_m ((R)-methylmalonyl-CoA)	86	13	µM		(Padovani & Banerjee, 2006)	
K_{cat}^+	255	17	s^{-1}		(Padovani & Banerjee, 2006)	
**(2R,3S)-2-methylmalyl-CoA hydro-lyase (2-methylfumaryl-CoA-forming)**						**9**
Stoichiometry: L-erythro-3-methylmalyl-CoA(aq) ⇌ mesaconyl-C1-CoA(aq) + H2O(l)						
K_{eq}	3.668				eQuilibrator	
M	43000		g mol^{-1}		(Borjian et al., 2017)	
Forward reaction:						
K_m (L-erythro-3-methylmalyl-CoA)	350	50	µM		(Borjian et al., 2017)	
K_{cat}^+	191	9	s^{-1}		(Borjian et al., 2017)	
Backward reaction:						
K_m (mesaconyl-C1-CoA)	1000	10	µM		(Borjian et al., 2017)	
K_{cat}^-	14.8	0.5	s^{-1}		(Borjian et al., 2017)	
**L-erythro-3-methylmalyl-CoA glyoxylate-lyase (propanoyl-CoA-forming)**						**10**
Stoichiometry: L-erythro-3-methylmalyl-CoA(aq) ⇌ propanoyl-CoA(aq) + glyoxylate(aq)						
K_{eq}	$1.622 \cdot 10^{-3}$				eQuilibrator	
M	36800		g mol^{-1}		(Erb et al., 2010)	
Forward reaction:						
K_m (L-erythro-3-methylmalyl-CoA)	10		µM		(Erb et al., 2010)	
K_{cat}^+	2.76		s^{-1}		(Erb et al., 2010)	
Backward reaction:						
K_m (propanoyl-CoA)	0.2		µM		(Erb et al., 2010)	
K_m (glyoxylate)	4.1		µM		(Erb et al., 2010)	
K_{cat}^-	12.27		s^{-1}		(Erb et al., 2010)	
**succinate semialdehyde:NADP+ oxidoreductase (CoA-acylating)**						**11**
Stoichiometry: succinate semialdehyde(aq) + CoA(aq) + NADP+(aq) ⇌ succinyl-CoA(aq) + NADPH(aq)						
K_{eq}	215.0				eQuilibrator	
M			g mol^{-1}		(Yoshida et al., 2016)	
Backward reaction:						
K_m (succinyl-CoA)	550	130	µM		(Yoshida et al., 2016)	
K_m (NADPH)	1420	190	µM		(Yoshida et al., 2016)	
K_{cat}^-	109	12	s^{-1}		(Yoshida et al., 2016)	
**4-hydroxybutanoate:NADP+ oxidoreductase**						**12**
Stoichiometry: 4-hydroxybutanoic acid(aq) + NADP+(aq) ⇌ succinate semialdehyde(aq) + NADPH(aq)						
K_{eq}	3.726				eQuilibrator	
M	134000		g mol^{-1}		(Meyer et al., 2015)	
Backward reaction:						
K_m (succinate semialdehyde)	5100	600	µM		(Meyer et al., 2015)	
K_m (NADPH)	1420	190	µM		(Meyer et al., 2015)	
K_{cat}^-	666	89	s^{-1}		(Meyer et al., 2015)	
**4-hydroxybutyrate---CoA ligase (ADP-forming)**						**13**
Stoichiometry: ATP(aq) + 4-hydroxybutanoate(aq) + CoA(aq) ⇌ ADP(aq) + Phosphate(aq) + 4-hydroxybutanoyl-CoA(aq)						
K_{eq}	971.9				eQuilibrator	
M	75600		g mol^{-1}		(Könneke et al., 2014)	
Forward reaction:						
K_m (4-hydroxybutyrate)	370	60	µM		(Könneke et al., 2014)	
K_m (ATP)	220	70	µM		(Könneke et al., 2014)	
K_m (CoA)	160	50	µM		(Könneke et al., 2014)	
K_{cat}^+	1.76	0.13	s^{-1}		(Könneke et al., 2014)	

Table S2 (continued)

**Enzyme name/ Parameter**	**Value**	**Uncer-tainty**	**Unit**	**Comment**	**Source**	**#**
**4-hydroxybutanoyl-CoA hydro-lyase**						**14**
Stoichiometry: 4-hydroxybutanoyl-CoA(aq) ⇌ crotonyl-CoA(aq) + H2O(l)						
K_{eq}	22.72				eQuilibrator	
M	56765		g mol^{-1}		Könneke et al., 2014), UniProt	
Forward reaction:						
K_m (4-hydroxybutyryl-CoA)	60	20	µM		(Könneke et al., 2014)	
K_{cat}^{+}	23.7	1.9	s^{-1}		(Könneke et al., 2014)	
**(2S)-methylsuccinyl-CoA:electron-transfer flavoprotein oxidoreductase**						**15**
Stoichiometry: methylsuccinyl-CoA(aq) + ubiquinone(aq) ⇌ mesaconyl-C1-CoA(aq) + ubinquinol(aq)						
K_{eq}	28000				eQuilibrator	
M	60000		g mol^{-1}		(Schwander et al., 2018)	
Forward reaction:						
K_m (methylsuccinyl-CoA)	80	5	µM	Electron acceptor unknown	(Schwander et al., 2018)	
K_{cat}^{+}	82.3	1.4	s^{-1}		(Schwander et al., 2018)	
**2-methylfumaryl-CoA 1,4-CoA-mutase**						**16**
Stoichiometry: mesaconyl-C1-CoA(aq) ⇌ mesaconyl-C4-CoA(aq)						
K_{eq}	1				eQuilibrator	
M	47000		g mol^{-1}		(Zarzycki et al., 2009)	
Forward reaction:						
K_m (mesaconyl-C1-CoA)	240		µM	T = 55°C	(Zarzycki et al., 2009)	
K_{cat}^{+}	407.3		s^{-1}	T = 55°C	(Zarzycki et al., 2009)	
**(S)-citramalyl-CoA hydro-lyase (3-methylfumaryl-CoA-forming)**						**17**
Stoichiometry: (3S)-citramalyl-CoA(aq) ⇌ mesaconyl-C4-CoA(aq) + H2O(l)						
K_{eq}	0.2782				eQuilibrator	
M	33000		g mol^{-1}		(Zarzycki et al., 2009)	
Backward reaction:						
K_m (mesaconyl-C4-CoA)	75		µM	T = 55°C	(Zarzycki et al., 2009)	
K_{cat}^{-}	522.5		s^{-1}	T = 55°C	(Zarzycki et al., 2009)	
**(3S)-citramalyl-CoA pyruvate-lyase (acetyl-CoA-forming)**						**18**
Stoichiometry: (3S)-citramalyl-CoA(aq) ⇌ acetyl-CoA(aq) + pyruvate(aq)						
K_{eq}	$1.293 \cdot 10^{-2}$				eQuilibrator	
M	29022		g mol^{-1}		(Sasikaran et al., 2014)	
Forward reaction:						
K_m (citramalyl-CoA)	30	9	µM		(Sasikaran et al., 2014)	
K_{cat}^{+}	82	10	s^{-1}		(Sasikaran et al., 2014)	
**pyruvate:carbon-dioxide ligase (ADP-forming)**						**19**
Stoichiometry: ATP(aq) + pyruvate(aq) + HCO3-(aq) ⇌ ADP(aq) + phosphate(aq) + oxaloacetate(aq)						
K_{eq}	24.68				eQuilibrator	
M	130000		g mol^{-1}		(Gurr & Jones, 1977)	
Forward reaction:						
K_m (pyruvate)	1860		µM		(Gurr & Jones, 1977)	
K_m (ATP)	80		µM		(Gurr & Jones, 1977)	
K_m (HCO_3^-)	330		µM		(Gurr & Jones, 1977)	
K_{cat}^{+}	58.5		s^{-1}		(Gurr & Jones, 1977)	
**(S)-malate:NAD+ oxidoreductase**						**20**
Stoichiometry: (S)-malate(aq) + NAD+(aq) ⇌ oxaloacetate(aq) + NADH(aq)						
K_{eq}	$2.11 \cdot 10^{-5}$				eQuilibrator	
M	40900		g mol^{-1}		(Muslin et al., 1995)	
Forward reaction:						
K_m (malate)	2600	200	µM		(Muslin et al., 1995)	
K_m (NAD^+)	260	30	µM		(Muslin et al., 1995)	
K_{cat}^{+}	21		s^{-1}		(Muslin et al., 1995)	
Backward reaction:						
K_m (oxaloacetate)	49	3	µM		(Muslin et al., 1995)	
K_m (NADH)	61	2	µM		(Muslin et al., 1995)	
K_{cat}^{-}	900		s^{-1}		(Muslin et al., 1995)	

Table S2 (continued)

Enzyme name/ Parameter	Value	Uncer-tainty	Unit	Comment	Source	#
**(S)-malate hydro-lyase (fumarate-forming)**						**21**
Stoichiometry: (S)-malate(aq) ⇌ fumarate(aq) + H2O(l)						
K_{eq}	0.2492				eQuilibrator	
M	60000		g mol^{-1}		(Ueda et al., 1991)	
Forward reaction:						
K_m (malate)	700	100	µM		(Ueda et al., 1991)	
K_{cat}^{+}	720	40	s^{-1}		(Ueda et al., 1991)	
Backward reaction:						
K_m (fumarate)	460	80	µM		(Ueda et al., 1991)	
K_{cat}^{-}	1900	100	s^{-1}		(Ueda et al., 1991)	
**succinate:CoA ligase (ADP-forming)**						**22**
Stoichiometry: ATP(aq) + succinate(aq) + CoA(aq) ⇌ ADP(aq) + phosphate(aq) + succinyl-CoA(aq)						
K_{eq}	1.745				eQuilibrator	
M	71170		g mol^{-1}		(Nolte et al., 2014)	
Forward reaction:						
K_m (succinate)	141	3	µM		(Nolte et al., 2014)	
K_m (ATP)	55	2	µM		(Nolte et al., 2014)	
K_m (CoA)	58	5	µM		(Nolte et al., 2014)	
K_{cat}^{+}	26.7	1.1	s^{-1}		(Nolte et al., 2014)	
**(2S)-ethylmalonyl-CoA:NADP+ oxidoreductase (decarboxylating)**						**23**
Stoichiometry: (2S)-ethylmalonyl-CoA(aq) + NADP+(aq) ⇌ crotonyl-CoA(aq) + CO2(aq) + NADPH(aq)						
K_{eq}	$2.5 \cdot 10^{-8}$				eQuilibrator	
M	48476		g mol^{-1}		UniProt	
Backward reaction:						
K_m (crotonyl-CoA)	21	2	µM		(Stoffel et al., 2019)	
K_m (NADPH)	37	4	µM		(Stoffel et al., 2019)	
K_m (CO2)	90	1	µM		(Stoffel et al., 2019)	
K_{cat}^{-}	103	3	s^{-1}		(Stoffel et al., 2019)	
**(2S)-ethylmalonyl-CoA epimerase**						**24**
Stoichiometry: (2S)-ethylmalonyl-CoA(aq) ⇌ (2R)-ethylmalonyl-CoA(aq)						
K_{eq}	1				eQuilibrator	
M	14260		g mol^{-1}		UniProt	
Forward reaction:						
K_m (S-ethylmalonyl-CoA)	40		µM		(Erb et al., 2008)	
K_{cat}^{+}	26.14		s^{-1}		(Erb et al., 2008)	
**(2R)-ethylmalonyl-CoA CoA-carbonylmutase**						**25**
Stoichiometry: (2R)-Ethylmalonyl-CoA(aq) ⇌ Methylsuccinyl-CoA(aq)						
K_{eq}	1				eQuilibrator	
M	74000		g mol^{-1}		(Erb et al., 2008)	
Forward reaction:						
K_m (R-ethylmalonyl-CoA)	60		µM		(Erb et al., 2008)	
K_{cat}^{+}	8.63		s^{-1}		(Erb et al., 2008)	
**succinate:menaquinone oxidoreductase**						**26**
Stoichiometry: succinate(aq) + menaquinone(aq) + 2 H+(out) ⇌ fumarate(aq) + menaquinol(aq) + 2 H+(in)						
K_{eq}	286.8			Assuming a membrane potential of 0.15 V & translocation of 2 H^+	eQuilibrator + calculation	
M	116499		g mol^{-1}		UniProt(*B. subtilis* protein)	
Forward reaction:						
K_m (succinate)	900		µM	*B. subtilis*	(Hederstedt & Heden, 1989)	
K_m (menaquinone)	1.5		µM	*E. coli* (no data *for B. subtilis*)	(Maklashina & Cecchini, 1999)	
K_{cat}^{+}	94		s^{-1}	*B. subtilis*	(Hederstedt & Heden, 1989)	
Backward reaction:						
K_m (fumarate)	20	2	µM	*E. coli* (no data *for B. subtilis*)	(Maklashina & Cecchini, 1999)	
K_m (menaquinol)	5.4		µM	*E. coli* (no data *for B. subtilis*)	(Maklashina & Cecchini, 1999)	
K_{cat}^{-}	23.5		s^{-1}	*B. subtilis*, extrapolated from cell extract activity and protein mass fraction	(Schnorpfeil et al., 2001) + calculations	

Table S2 (continued)

Enzyme name/ Parameter	Value	Uncer-tainty	Unit	Comment	Source	#
**acetyl-CoA:hydrogencarbonate ligase (ADP-forming)**						**27**
Stoichiometry: acetyl-CoA(aq) + $HCO3^-$(aq) + ATP(aq) <=> malonyl-CoA(aq) + ADP(aq) + phosphate(aq)						
K_{eq}	33.05				eQuilibrator	
M	2270000		g mol^{-1}		(Price et al., 2003)	
Forward reaction:						
K_m (acetyl-CoA)	120	10	µM		(Price et al., 2003)	
K_m (ATP)	80	10	µM		(Price et al., 2003)	
K_m (HCO_3^-)	1009	70	µM		(Price et al., 2003)	
K_{cat}^+	8210		s^{-1}		(Price et al., 2003)	
**malonyl-CoA:NADP+ oxidoreductase (3-Hydroxypropanoate-forming)**						**28**
Stoichiometry: 3-hydroxypropanoate(aq) + CoA(aq) + 2 NADP+(aq) ⇌ malonyl-CoA(aq) + 2 NADPH(aq)						
K_{eq}	0.02967				eQuilibrator	
M	300000		g mol^{-1}		(Hügler et al., 2002)	
Backward reaction:						
K_m (malonyl-CoA)	30		µM		(Hügler et al., 2002)	
K_m (NADPH)	25		µM		(Hügler et al., 2002)	
K_{cat}^-	50		s^{-1}		(Hügler et al., 2002)	
**hydroxypropanoate:CoA ligase (AMP-forming)/acrylyl-CoA:NADP+ oxidoreductase (propanoyl-CoA-forming)**						**29**
Stoichiometry: 3-hydroxypropanoate(aq) + ATP(aq) + CoA(aq) + NADPH(aq) ⇌ propanoyl-CoA(aq) + AMP(aq) + PP_i(aq) + NADP+(aq)						
K_{eq}	$2.04 \cdot 10^{10}$				eQuilibrator	
M	200000		g mol^{-1}		(Bernhardsgrütter et al., 2018)	
Forward reaction:						
K_m (3-hydroxypropionate)	200	20	µM		(Bernhardsgrütter et al., 2018)	
K_m (ATP)	340	140	µM		(Bernhardsgrütter et al., 2018)	
K_m (CoA)	220	50	µM		(Bernhardsgrütter et al., 2018)	
K_m (NADPH)	20	3	µM		(Bernhardsgrütter et al., 2018)	
K_{cat}^+	5.1	0.2	s^{-1}		(Bernhardsgrütter et al., 2018)	
**hydroxypropanoate:CoA ligase (ADP-forming)**						**30**
Stoichiometry: 3-hydroxypropanoate(aq) + ATP(aq) + CoA(aq) ⇌ 3-hydroxypropanoyl-CoA(aq) + ADP(aq) + phosphate(aq) + NADP(aq)						
K_{eq}	0.01104				eQuilibrator	
M	76000		g mol^{-1}		(Könneke et al., 2014)	
Forward reaction:						
K_m (3-hydroxypropionate)	1200	200	µM		(Könneke et al., 2014)	
K_m (ATP)	600	100	µM		(Könneke et al., 2014)	
K_m (CoA)	160	90	µM		(Könneke et al., 2014)	
K_{cat}^+	0.747		s^{-1}		(Könneke et al., 2014)	
Backward reaction:						
K_m (phosphate)	5000	5000	µM	A parameter prior of 5 mM for phosphate was used	This study	
**propanoyl-CoA:NADP+ oxidoreductase/ hydroxypropanoyl-CoA dehydratase**						**31**
Stoichiometry: propanoyl-CoA(aq) + NADP+(aq) + H2O(l) ⇌ 3-hydroxypropanoyl-CoA(aq) + NADPH(aq)						
K_{eq}	$6.87 \cdot 10^{-11}$				eQuilibrator	
M	133000		g mol^{-1}		(Bernhardsgrütter et al., 2018), only 2/3 of the enzyme are needed	
Backward reaction:						
K_m (3-hydroxypropanoyl-CoA)	200	20	µM	It was assumed that the Km would be in the order of 3-hydroxypropanoate of the original enzyme	(Bernhardsgrütter et al., 2018)	
K_m (NADPH)	20	3	µM		(Bernhardsgrütter et al., 2018)	
K_{cat}^-	11.7	1	s^{-1}		(Bernhardsgrütter et al., 2018)	
**4-hydroxybutyrate---CoA ligase (AMP-forming)**						**32**
Stoichiometry: ATP(aq) + 4-hydroxybutanoate(aq) + CoA(aq) ⇌ AMP(aq) + diphosphate(aq) + 4-hydroxybutanoyl-CoA(aq)						
K_{eq}	$1.237 \cdot 10^5$				eQuilibrator	
M	64500		g mol^{-1}		(Hawkins et al., 2014)	
Forward reaction:						
K_m (4-hydroxybutanoate)	2000	400	µM	T = 70°C	(Hawkins et al., 2014)	
K_{cat}^+	1.82	0.12	s^{-1}	T = 70°C	(Hawkins et al., 2014)	

Table S2 (continued)

Enzyme name/ Parameter	Value	Uncer-tainty	Unit	Comment	Source	#
**(S)-3-hydroxyacyl-CoA:NAD+ oxidoreductase**						**33**
Stoichiometry: (S)-3-hydroxybutanoyl-CoA(aq) + NAD+(aq) ⇌ acetoacetyl-CoA(aq) + NADH(aq)						
K_{eq}	$3.043 \cdot 10^{-3}$				eQuilibrator	
M	79594		g mol^{-1}		UniProt	
Forward reaction:						
K_m (NAD^+)	830		µM		(Shimakata et al., 1979)	
Backward reaction:						
K_m (acetoacetyl-CoA)	66		µM		(Binstock & Schulz, 1981)	
K_m (NADH)	33		µM		(Shimakata et al., 1979)	
K_{cat}^-	84.9		s^{-1}		(Binstock & Schulz, 1981)	
**succinyl-CoA:(S)-malate CoA-transferase**						**34**
Stoichiometry: succinyl-CoA(aq) + (S)-malate(aq) ⇌ succinate(aq) + (r,s)-malyl-coa(aq)						
K_{eq}	6.877				eQuilibrator	
M	90800		g mol^{-1}		(Friedmann et al., 2006)	
Forward reaction:						
K_m (succinyl-CoA)	500	100	µM	T = 55°C	(Friedmann et al., 2006)	
K_m ((S)-malate)	1300	300	µM	T = 55°C	(Friedmann et al., 2006)	
K_{cat}^+	11.35		s^{-1}	T = 55°C	(Friedmann et al., 2006)	
**L-malyl-CoA glyoxylate-lyase (acetyl-CoA-forming)**						**35**
Stoichiometry: L-malyl-CoA(aq) ⇌ acetyl-CoA(aq) + glyoxylate(aq)						
K_{eq}	$2.801 \cdot 10^{-3}$				eQuilibrator	
M	204000		g mol^{-1}		(Meister et al., 2005)	
Forward reaction:						
K_m ((r,s)-malyl-coa(aq))	15	3	µM		(Meister et al., 2005)	
K_{cat}^+	19.38	0.68	s^{-1}		(Meister et al., 2005)	
Backward reaction:						
K_m (acetyl-CoA)	140	10	µM		(Meister et al., 2005)	
K_{cat}^-	126	3	s^{-1}		(Meister et al., 2005)	
**3-phospho-D-glycerate carboxy-lyase (dimerizing; D-ribulose-1,5-bisphosphate-forming)**						**36**
Stoichiometry: 2 3-phospho-D-glycerate(aq) ⇌ D-ribulose-1,5-bisphosphate(aq) + CO2(aq) + H2O(l)						
K_{eq}	$8.14 \cdot 10^{-7}$				eQuilibrator	
M	72268		g mol^{-1}		UniProt	
Backward reaction:						
K_m (ribulose-1,5-bisphosphate)	30	4	µM		(Carmo-Silva et al., 2010)	
K_m (CO2)	10.9	0.9	µM		(Nakano et al., 2010)	
K_{cat}^-	3.06	0.08	s^{-1}		(Carmo-Silva et al., 2010)	
**ATP:3-phospho-D-glycerate 1-phosphotransferase**						**37**
Stoichiometry: ATP(aq) + 3-phospho-D-glycerate(aq) ⇌ ADP(aq) + 3-phospho-D-glyceroyl phosphate(aq)						
K_{eq}	$5.257 \cdot 10^{-4}$				eQuilibrator	
M	41784		g mol^{-1}		UniProt	
Forward reaction:						
K_m (3-phosphoglycerate)	180	20	µM		(Tsukamoto et al., 2013)	
K_m (ATP)	210	12	µM		(Tsukamoto et al., 2013)	
K_{cat}^+	403	19	s^{-1}		(Tsukamoto et al., 2013)	
**D-glyceraldehyde-3-phosphate:NA(D)P+ oxidoreductase (phosphorylating)**						**38**
Stoichiometry:<br>D-glyceraldehyde 3-phosphate(aq) + phosphate(aq) + NA(D)P+(aq) ⇌ 3-phospho-D-glyceroyl phosphate(aq) + NA(D)PH(aq)						
K_{eq}	0.4176				eQuilibrator	
M	150000		g mol^{-1}		(Erales et al., 2008)	
Forward reaction:						
K_m (phosphate)	5000	5000	µM		This work	
Backward reaction:						
K_m (1,3-bisphosphoglycerate)	210	12	µM		(Erales et al., 2008)	
K_m (NADPH)	140	6	µM		(Erales et al., 2008)	
K_{cat}^-	815	18	s^{-1}		(Erales et al., 2008)	
**D-glyceraldehyde-3-phosphate aldose-ketose-isomerase**						**39**
Stoichiometry: D-glyceraldehyde 3-phosphate(aq) ⇌ glycerone phosphate(aq)						
K_{eq}	9.354				eQuilibrator	
M	28213		g mol^{-1}		(Mathur et al., 2006)	
Forward reaction:						
K_m (glyceraldehyde-3-phosphate)	84		µM		(Mathur et al., 2006)	
K_{cat}^+	2680		s^{-1}		(Mathur et al., 2006)	

Table S2 (continued)

Enzyme name/ Parameter	Value	Uncer-tainty	Unit	Comment	Source	#
**D-fructose-1,6-bisphosphate D-glyceraldehyde-3-phosphate-lyase (glycerone-phosphate-forming)**						**40**
Stoichiometry: D-fructose-1,6-bisphosphate(aq) ⇌ glycerone phosphate(aq) + D-glyceraldehyde 3-phosphate(aq)						
K_{eq}	$1.591 \cdot 10^{-4}$				eQuilibrator	
M	66000		g mol^{-1}		(Nakahara et al., 2003)	
Forward reaction:						
K_m (fructose-1,6-bisphosphate)	7		µM		(Nakahara et al., 2003)	
K_{cat}^{+}	15.8		s^{-1}		(Nakahara et al., 2003)	
**D-fructose-1,6-bisphosphate 1-phosphohydrolase**						**41**
Stoichiometry: D-fructose-1,6-bisphosphate(aq) + H2O(l) ⇌ D-fructose-6-phosphate(aq) + phosphate(aq)						
K_{eq}	41.47				eQuilibrator	
M	36520		g mol^{-1}		(Kelley-Loughnane et al., 2002)	
Forward reaction:						
K_m (fructose-1,6-bisphosphate)	16	2	µM		(Kelley-Loughnane et al., 2002)	
K_{cat}^{+}	14.6	0.8	s^{-1}		(Kelley-Loughnane et al., 2002)	
**D-fructose 6-phosphate:D-glyceraldehyde-3-phosphate glycolaldehyde transferase**						**42**
Stoichiometry: D-fructose-6-phosphate(aq) + D-glyceraldehyde 3-phosphate(aq) ⇌ D-erythrose 4-phosphate(aq) + D-xylulose-5-phosphate(aq)						
K_{eq}	$1.673 \cdot 10^{-2}$				eQuilibrator	
M	74143		g mol^{-1}		(Sprenger et al., 1995)	
Forward reaction:						
K_m (fructose-6-phosphate)	1100		µM		(Sprenger et al., 1995)	
K_m (glyceraldehyde-3-phosphate)	2300		µM		(Sprenger et al., 1995)	
K_{cat}^{+}	62.28		s^{-1}		(Sprenger et al., 1995)	
Backward reaction:						
K_m (erythrose-4-phosphate)	90		µM		(Sprenger et al., 1995)	
K_m (xylulose-5-phosphate)	160		µM		(Sprenger et al., 1995)	
K_{cat}^{-}	135.9		s^{-1}		(Sprenger et al., 1995)	
**sedoheptulose 1,7-bisphosphate D-glyceraldehyde-3-phosphate-lyase**						**43**
Stoichiometry: sedoheptulose-1,7-bisphosphate(aq) ⇌ glycerone phosphate(aq) + D-erythrose 4-phosphate(aq)						
K_{eq}	$3.371 \cdot 10^{-3}$				eQuilibrator	
M	33000		g mol^{-1}		(Nakahara et al., 2003)	
Forward reaction:						
K_m (sedoheptulose-1,7-bisphosphate)	47		µM		(Nakahara et al., 2003)	
K_{cat}^{+}	1.746		s^{-1}		(Nakahara et al., 2003)	
**sedoheptulose-1,7-bisphosphate 1-phosphohydrolase**						**44**
Stoichiometry: sedoheptulose-1,7-bisphosphate(aq) + H2O(l) ⇌ sedoheptulose-7-phosphate(aq) + phosphate(aq)						
K_{eq}	669.4				eQuilibrator	
M	42081		g mol^{-1}		UniProt	
Forward reaction:						
K_m (sedoheptulose-1,7-bisphosphate)	50		µM		(Cadet & Meunier, 1988)	
K_{cat}^{+}	81		s^{-1}		(Cadet & Meunier, 1988)	
**sedoheptulose-7-phosphate:D-glyceraldehyde-3-phosphate glycolaldehydetransferase**						**45**
Stoichiometry: sedoheptulose-7-phosphate(aq) + D-glyceraldehyde 3-phosphate(aq) ⇌ D-ribose-5-phosphate(aq) + D-xylulose-5-phosphate(aq)						
K_{eq}	0.2104				eQuilibrator	
M	74143		g mol^{-1}		(Sprenger et al., 1995)	
Forward reaction:						
K_m (sedoheptulose-7-phosphate)	4000		µM		(Sprenger et al., 1995)	
K_m (glyceraldehyde 3-phosphate)	2100		µM		(Sprenger et al., 1995)	
K_{cat}^{+}	7.414		s^{-1}		(Sprenger et al., 1995)	
Backward reaction:						
K_m (ribose-5-phosphate)	1500		µM		(Sprenger et al., 1995)	
K_m (xylulose-5-phosphate)	160		µM		(Sprenger et al., 1995)	
**D-ribulose-5-phosphate 3-epimerase**						**46**
Stoichiometry: D-ribulose-5-phosphate(aq) ⇌ D-xylulose-5-phosphate(aq)						
K_{eq}	3.897				eQuilibrator	
M	23334		g mol^{-1}		UniProt	
Forward reaction:						
K_m (ribulose-5-phosphate)	56.0	4.4	µM		(Le et al., 2017)	
K_{cat}^{+}	42.8	1.3	s^{-1}		(Le et al., 2017)	

Table S2 (continued)

Enzyme name/ Parameter	Value	Uncer-tainty	Unit	Comment	Source	#
**D-ribose-5-phosphate aldose-ketose-isomerase**						**47**
Stoichiometry: D-ribose-5-phosphate(aq) ⇌ D-ribulose-5-phosphate(aq)						
K_{eq}	0.4421				eQuilibrator	
M	45720		g mol^{-1}		(Zhang et al., 2003), UniProt	
Forward reaction:						
K_m (ribose-5-phosphate)	3100	200	µM		(Zhang et al., 2003)	
K_{cat}^+	2100	300	s^{-1}		(Zhang et al., 2003)	
**ATP:D-ribulose-5-phosphate 1-phosphotransferase**						**48**
Stoichiometry: ATP + D-ribulose 5-phosphate = ADP + D-ribulose 1,5-bisphosphate						
K_{eq}	9583				eQuilibrator	
M	178000		g mol^{-1}		(Wadano et al., 1998)	
Forward reaction:						
K_m (ATP)	90		µM		(Wadano et al., 1998)	
K_m (ribulose 5-phosphate)	270		µM		(Wadano et al., 1998)	
K_{cat}^+	215.4		s^{-1}		(Wadano et al., 1998)	
**phosphate:oxaloacetate carboxy-lyase (adding phosphate, phosphoenolpyruvate-forming)**						**49**
Stoichiometry: phosphate(aq) + oxaloacetate(aq) ⇌ phosphoenolpyruvate(aq) + HCO_3^-(aq)						
K_{eq}	$1.863 \cdot 10^{-6}$				eQuilibrator	
M	114000		g mol^{-1}		(Chang et al., 2014)	
Backward reaction:						
K_m (phosphoenolpyruvate)	790	90	µM		(Chang et al., 2014)	
K_m (HCO_3^-)	169		µM		(Chang et al., 2014)	
K_{cat}^-	42	3	s^{-1}		(Chang et al., 2014)	
**2-phospho-D-glycerate hydro-lyase (phosphoenolpyruvate-forming)**						**50**
Stoichiometry: 2-phospho-D-glycerate(aq) ⇌ phosphoenolpyruvate(aq) + H2O(l)						
K_{eq}	5.191				eQuilibrator	
M	46000		g mol^{-1}		(Zadvornyy et al., 2015)	
Forward reaction:						
K_m (phospho-D-glycerate)	160	10	µM		(Zadvornyy et al., 2015)	
K_{cat}^+	95	4	s^{-1}		(Zadvornyy et al., 2015)	
**D-phosphoglycerate 2,3-phosphomutase (2,3-diphosphoglycerate-independent)**						**51**
Stoichiometry: 2-phospho-D-glycerate(aq) ⇌ 3-phospho-D-glycerate(aq)						
K_{eq}	5.300				eQuilibrator	
M	64000		g mol^{-1}		(GRAÑA et al., 1989)	
Forward reaction:						
K_m (2-phospho-D-glycerate)	369	20	µM		(GRAÑA et al., 1989)	
Backward reaction:						
K_m (3-phospho-D-glycerate)	324	20	µM		(GRAÑA et al., 1989)	
K_{cat}^-	216		s^{-1}		(GRAÑA et al., 1989)	
**D-arabino-hex-3-ulose-6-phosphate formaldehyde-lyase (D-ribulose-5-phosphate-forming)**						**52**
Stoichiometry: D-arabino-hex-3-ulose-6-phosphate(aq) ⇌ D-ribulose-5-phosphate(aq) + formaldehyde(aq)						
K_{eq}	$2.312 \cdot 10^{-5}$				eQuilibrator	
M	27000		g mol^{-1}		(Arfman et al., 1990)	
Backward reaction:						
K_m (ribulose-5-phosphate)	450		µM		(Arfman et al., 1990)	
K_m (formaldehyde)	147		µM		(Arfman et al., 1990)	
K_{cat}^-	216		s^{-1}		(Arfman et al., 1990)	
**D-arabino-hex-3-ulose-6-phosphate isomerase**						**53**
Stoichiometry: D-arabino-hex-3-ulose-6-phosphate(aq) ⇌ D-fructose-6-phosphate(aq)						
K_{eq}	305.9				eQuilibrator	
M	67000		g mol^{-1}		(Ferenci et al., 1974)	
Forward reaction:						
K_m (D-arabino-hex-3-ulose-6-phosphate)	100		µM		(Ferenci et al., 1974)	
K_{cat}^+	1742		s^{-1}		(Ferenci et al., 1974)	
Backward reaction:						
K_m (fructose-6-phosphate)	1100		µM		(Ferenci et al., 1974)	
**methanol:NAD+ oxidoreductase**						**54**
Stoichiometry: methanol(aq) + NAD+(aq) ⇌ formaldehyde(aq) + NADH(aq)						
K_{eq}	$4.368 \cdot 10^{-6}$				eQuilibrator	
M	40700		g mol^{-1}		(Wu et al., 2016)	
Forward reaction:						
K_m (methanol)	21600	1500	µM		(Wu et al., 2016)	
K_{cat}^+	0.06	0.003	s^{-1}	Activity scaled down to appropriate pH	(Wu et al., 2016)	

Table S2 (continued)

Enzyme name/ Parameter	Value	Uncer-tainty	Unit	Comment	Source	#
**formaldehyde:NADP+ oxidoreductase (CoA-acetylating)**						**55**
Stoichiometry: formaldehyde(aq) + CoA(aq) + NADP+(aq) ⇌ formyl-CoA(aq) + NADPH(aq)						
K_{eq}	$5.737 \cdot 10^5$				eQuilibrator	
M	52000		g mol^{-1}		(Chou et al., 2019)	
Forward reaction:						
K_m (formaldehyde)	10400	2200	µM		(Chou et al., 2019)	
K_{cat}^+	0.99	0.07	s^{-1}		(Chou et al., 2019)	
**formyl-CoA:oxalate CoA-transferase**						**56**
Stoichiometry: formyl-CoA(aq) + succinate(aq) ⇌ formate(aq) + succinyl-CoA(aq)						
K_{eq}	0.01888				eQuilibrator	
M	44000		g mol^{-1}		(Baetz & Allison, 1990)	
Forward reaction:						
K_m (formyl-CoA)	3000	500	µM		(Baetz & Allison, 1990)	
K_m (succinate)	2300	600	µM		(Baetz & Allison, 1990)	
K_{cat}^+	14.08		s^{-1}		(Baetz & Allison, 1990)	
**ATP:D-fructose-6-phosphate 1-phosphotransferase**						**57**
Stoichiometry: ATP(aq) + D-fructose-6-phosphate(aq) ⇌ ADP(aq) + D-fructose-1,6-bisphosphate(aq)						
K_{eq}	1997				eQuilibrator	
M	37000		g mol^{-1}		(Kotlarz & Buc, 1982)	
Forward reaction:						
K_m (ATP)	50		µM		(Kotlarz & Buc, 1982)	
K_m (fructose-6-phosphate)	11		µM		(Kotlarz & Buc, 1982)	
K_{cat}^+	126.4		s^{-1}		(Kotlarz & Buc, 1982)	
**L-serine formaldehyde-lyase (glycine-forming)**						**58**
Stoichiometry: L-serine(aq) ⇌ glycine(aq) + formaldehyde(aq)						
K_{eq}	$4.855 \cdot 10^{-4}$				eQuilibrator	
M	48000		g mol^{-1}		(MIYAZAKI et al., 1987)	
Forward reaction:						
K_m (serine)	150		µM		(MIYAZAKI et al., 1987)	
K_{cat}^+	10.8		s^{-1}		(MIYAZAKI et al., 1987)	
Backward reaction:						
K_m (glycine)	46		µM		(MIYAZAKI et al., 1987)	
K_m (formaldehyde)	4800		µM		(MIYAZAKI et al., 1987)	
K_{cat}^-	264		s^{-1}		(MIYAZAKI et al., 1987)	
**Formate:tetrahydrofolate ligase (ADP-forming)**						**59**
Stoichiometry: tetrahydrofolate(aq) + formate(aq) + ATP(aq) ⇌ ADP(aq) + orthophosphate(aq) + 10-formyltetrahydrofolate(aq)						
K_{eq}	3.696				eQuilibrator	
M	240000		g mol^{-1}		(Marx et al., 2003)	
Forward reaction:						
K_m (tetrahydrofolate)	800		µM		(Marx et al., 2003)	
K_m (formate)	22000		µM		(Marx et al., 2003)	
K_m (ATP)	21		µM		(Marx et al., 2003)	
K_{cat}^+	468		s^{-1}		(Marx et al., 2003)	
**5,10-Methenyltetrahydrofolate 5-hydrolase (decyclizing)**						**60**
Stoichiometry: 5,10-methenyltetrahydrofolate(aq) + H2O(l) ⇌ 10-formyltetrahydrofolate(aq)						
K_{eq}	10.93				eQuilibrator	
M	22000		g mol^{-1}		(Pomper et al., 1999)	
Forward reaction:						
K_m (5,10-methenyltetra-hydrofolate)	35	4	µM		(Pomper et al., 1999)	
K_{cat}^+	231		s^{-1}		(Pomper et al., 1999)	
**5,10-methylenetetrahydrofolate:NADP+ oxidoreductase**						**61**
Stoichiometry: 5,10-methylenetetrahydrofolate(aq) + NADP+(aq) ⇌ 5,10-methenyltetrahydrofolate(aq) + NADPH(aq)						
K_{eq}	0.07062				eQuilibrator	
M	60000		g mol^{-1}		(Dev & Harvey, 1978)	
Forward reaction:						
K_m (NADP)	53	5	µM		(Dev & Harvey, 1978)	
K_m (5,10-methylenetetra-hydrofolate)	35	4	µM		(Dev & Harvey, 1978)	
K_{cat}^+	97.6	6	s^{-1}		(Dev & Harvey, 1978)	

Table S2 (continued)

Enzyme name/ Parameter	Value	Uncer-tainty	Unit	Comment	Source	#
**5,10-methylenetetrahydrofolate:glycine hydroxymethyltransferase**						**62**
Stoichiometry: 5,10-methylenetetrahydrofolate(aq) + glycine(aq) + H2O(l) ⇌ tetrahydrofolate(aq) + L-serine(aq)						
K_{eq}	0.06931				eQuilibrator	
M	94000		g mol^{-1}		(Schirch et al., 1985)	
Forward reaction:						
K_m (5,10-methylenetetra-hydrofolate)	33		µM		(Schirch et al., 1985)	
K_m (glycine)	850		µM		(Schirch et al., 1985)	
Backward reaction:						
K_m (tetrahydrofolate)	80		µM		(Schirch et al., 1985)	
K_m (serine)	800		µM		(Schirch et al., 1985)	
K_{cat}^-	21.3		s^{-1}		(Schirch et al., 1985)	
**L-serine:glyoxylate aminotransferase**						**63**
Stoichiometry: L-serine(aq) + glyoxylate(aq) ⇌ 3-hydroxypyruvate(aq) + glycine(aq)						
K_{eq}	5.502				eQuilibrator	
M	150000		g mol^{-1}		(IZUMI et al., 1990)	
Forward reaction:						
K_m (serine)	280	20	µM		(Karsten et al., 2001)	
K_m (glyoxylate)	230		µM		(IZUMI et al., 1990)	
K_{cat}^+	173.75		s^{-1}		(IZUMI et al., 1990)	
**D-glycerate:NAD+ oxidoreductase**						**64**
Stoichiometry: D-glycerate + NAD+ = hydroxypyruvate + NADH + H+						
K_{eq}	$1.132 \cdot 10^{-5}$				eQuilibrator	
M	71000		g mol^{-1}		(Chistoserdova & Lidstrom, 1991)	
Forward reaction:						
K_m (glycerate)	2600		µM		(Chistoserdova & Lidstrom, 1991)	
K_{cat}^+	11.98		s^{-1}		(Chistoserdova & Lidstrom, 1991)	
Backward reaction:						
K_m (hydroxypyruvate)	40		µM		(Chistoserdova & Lidstrom, 1991)	
K_m (NADH)	100		µM		(Chistoserdova & Lidstrom, 1991)	
K_{cat}^-	798.8		s^{-1}		(Chistoserdova & Lidstrom, 1991)	
**ATP:D-glycerate 2-phosphotransferase**						**65**
Stoichiometry: ATP(aq) + D-glycerate(aq) ⇌ ADP(aq) + 2-phospho-D-glycerate(aq)						
K_{eq}	49.65				eQuilibrator	
M	39000		g mol^{-1}		UniProt	
Forward reaction:						
K_m (ATP)	121	16	µM		(Bartsch et al., 2008)	
K_m (glycerate)	86	1	µM		(Bartsch et al., 2008)	
K_{cat}^+	302	7	s^{-1}		(Bartsch et al., 2008)	
**(S)-methylmalonyl-CoA:pyruvate carboxytransferase**						**66**
Stoichiometry: malonyl-CoA(aq) + pyruvate(aq) ⇌ acetyl-CoA(aq) + oxaloacetate(aq)						
K_{eq}	0.7468				eQuilibrator	
M	670000		g mol^{-1}		(Wood et al., 1969)	
Forward reaction:						
K_m (malonyl-CoA)	350		µM		(Wood et al., 1969)	
K_m (pyruvate)	760		µM		(Wood et al., 1969)	
Backward reaction:						
K_m (acetyl-CoA)	560		µM		(Wood et al., 1969)	
K_m (oxaloacetate)	570		µM		(Wood et al., 1969)	
K_{cat}^-	233		s^{-1}		(Wood et al., 1969)	
**[protein]-S8-aminomethyldihydrolipoyllysine:tetrahydrofolate aminomethyltransferase (ammonia-forming) (T-protein)**						**67**
Stoichiometry: tetrahydrofolate(aq) + S(8)-aminomethyldihydrolipoamide(aq) ⇌ NH_3(aq) + 5,10-methylenetetrahydrofolate(aq) + dihydrolipoamide(aq)						
K_{eq}	0.3198				eQuilibrator	
M	40147		g mol^{-1}		UniProt	
Backward reaction:						
K_m (NH_3)	9.2		µM		(Okamura-Ikeda et al., 2003)	
K_m (5,10-methylenetetra-hydrofolate)	88.1		µM		(Okamura-Ikeda et al., 2003)	
K_m (dihydrolipoamide)	0.69		µM		(Okamura-Ikeda et al., 2003)	
K_{cat}^-	19.4		s^{-1}		(Okamura-Ikeda et al., 2003)	

Table S2 (continued)

Enzyme name/ Parameter	Value	Uncer-tainty	Unit	Comment	Source	#
**glycine:H-protein-lipoyllysine oxidoreductase (decarboxylating, acceptor-amino-methylating) (P-protein)**						**68**
Stoichiometry: glycine(aq) + lipoamide(aq) ⇌ CO2(aq) + S(8)-aminomethyldihydrolipoamide(aq)						
K_{eq}	$9.202 \cdot 10^{-4}$				eQuilibrator	
M	100000		g mol^{-1}		(Fujiwara & Motokawa, 1983)	
Forward reaction:						
K_m (glycine)	5800		µM		(Fujiwara & Motokawa, 1983)	
K_m (lipoamide)	3.4		µM		(Fujiwara & Motokawa, 1983)	
K_{cat}^{+}	6.8		s^{-1}		(Fujiwara & Motokawa, 1983)	
Backward reaction:						
K_m (CO2)	5400		µM		(Fujiwara & Motokawa, 1983)	
K_m (S(8)-aminomethyl-dihydrolipoamide)	1.6		µM		(Fujiwara & Motokawa, 1983)	
**protein-N6-(dihydrolipoyl)lysine:NAD+ oxidoreductase (L-protein)**						**69**
Stoichiometry: lipoamide(aq) + NADH(aq) ⇌ dihydrolipoamide(aq) + NAD+(aq)						
K_{eq}	1.907				eQuilibrator	
M	126000		g mol^{-1}		(Neuburger et al., 2000)	
Backward reaction:						
K_m (dihydrolipoamide)	27	3	µM		(Neuburger et al., 2000)	
K_{cat}^{-}	345	10	s^{-1}		(Neuburger et al., 2000)	
**malyl-CoA lyase (formyl-CoA forming)**						**70**
Stoichiometry: malonate semialdehyde(aq) + formyl-CoA(aq) ⇌ malyl-CoA(aq)						
K_{eq}	110.8				eQuilibrator	
M	59116		g mol^{-1}		(Chou et al., 2019)	
Forward reaction:						
K_m (malonate semialdehyde)	16000	4000	µM	It was assumed that malonate semi-aldehyde behaves similar to propanal	(Chou et al., 2019)	
K_m (formyl-CoA)	200	50	µM		(Chou et al., 2019)	
K_{cat}^{+}	4.7	0.4	s^{-1}		(Chou et al., 2019)	
**2-hydroxyglutaryl-CoA lyase (formyl-CoA forming)**						**71**
Stoichiometry: succinic semialdehyde(aq) + formyl-CoA(aq) ⇌ 2-hydroxyglutaryl-CoA(aq)						
K_{eq}	105.1				eQuilibrator	
M	59116		g mol^{-1}		(Chou et al., 2019)	
Forward reaction:						
K_m (succinic semialdehyde)	2500	600	µM	It was assumed that succinic semialde-hyde behaves like a mixture of propanal and pentanal	(Chou et al., 2019)	
K_m (formyl-CoA)	200	50	µM		(Chou et al., 2019)	
K_{cat}^{+}	3.1	0.4	s^{-1}		(Chou et al., 2019)	
**succinyl-CoA:2-hydroxyglutarate CoA-transferase**						**72**
Stoichiometry: 2-hydroxyglutaryl-CoA(aq) + succinate(aq) ⇌ 2-hydroxyglutarate(aq) + succinyl-CoA(aq)						
K_{eq}	0.2827				eQuilibrator	
M	90800		g mol^{-1}		(Friedmann et al., 2006)	
Backward reaction:						
K_m (2-hydroxyglutarate)	1300	300	µM	It was assumed that 2-hydroxyglutarate behaves similar to 2-hydroxysuccinate (malate). Note that the CoA will be transferred to the other carboxy group	(Friedmann et al., 2006)	
K_m (succinyl-CoA)	500	100	µM		(Friedmann et al., 2006)	
K_{cat}^{-}	11.35		s^{-1}		(Friedmann et al., 2006)	

Table S2 (continued)

Enzyme name/ Parameter	Value	Uncer-tainty	Unit	Comment	Source	#
**2-hydroxyglutarate oxaloacetate transhydrogenase**						**73**
Stoichiometry: oxaloacetate(aq) + 2-hydroxyglutarate(aq) ⇌ malate(aq) + alpha-ketoglutarate(aq)						
K_{eq}	22.58				eQuilibrator	
M	99000		g mol^{-1}		(Allen, 1966)	
Forward reaction:						
K_m (oxaloacetate)	50		µM		(Allen, 1966)	
K_m (2-hydroxyglutarate)	1400		µM		(Allen, 1966)	
K_{cat}^+	1.98		s^{-1}		(Allen, 1966)	
Backward reaction:						
K_m (malate)	1400		µM		(Allen, 1966)	
K_m (alpha-ketoglutarate)	50		µM		(Allen, 1966)	
**isocitrate:NADP+ oxidoreductase (decarboxylating)**						**74**
Stoichiometry: isocitrate(aq) + NADP+(aq) ⇌ 2-oxoglutarate(aq) + CO2(aq) + NADPH(aq)						
K_{eq}	0.08955				eQuilibrator	
M	80000		g mol^{-1}		(Kanao et al., 2002)	
Forward reaction:						
K_m (isocitrate)	45	13	µM		(Kanao et al., 2002)	
K_m (NADP)	27	10	µM		(Kanao et al., 2002)	
K_{cat}^+	200	8	s^{-1}		(Kanao et al., 2002)	
Backward reaction:						
K_m (2-oxoglutarate)	1100	500	µM		(Kanao et al., 2002)	
K_m (CO2)	1300	300	µM		(Kanao et al., 2002)	
K_{cat}^-	51	12	s^{-1}		(Kanao et al., 2002)	
**isocitrate hydro-lyase (cis-aconitate-forming)**						**75**
Stoichiometry: isocitrate(aq) ⇌ cis-aconitate(aq) + H2O(l)						
K_{eq}	0.7572				eQuilibrator	
M	102000		g mol^{-1}		(Baumgart & Bott, 2011)	
Forward reaction:						
K_m (isocitrate)	552	302	µM		(Baumgart & Bott, 2011)	
K_{cat}^+	28.05		s^{-1}		(Baumgart & Bott, 2011)	
Backward reaction:						
K_m (cis-aconitate)	18.5	3.4	µM		(Baumgart & Bott, 2011)	
K_{cat}^-	69.02		s^{-1}		(Baumgart & Bott, 2011)	
**citrate hydro-lyase (cis-aconitate-forming)**						**76**
Stoichiometry: citrate(aq) ⇌ cis-aconitate(aq) + H2O(l)						
K_{eq}	0.03482				eQuilibrator	
M	102000		g mol^{-1}		(Baumgart & Bott, 2011)	
Forward reaction:						
K_m (citrate)	480	20	µM		(Baumgart & Bott, 2011)	
K_{cat}^+	19.04		s^{-1}		(Baumgart & Bott, 2011)	
Backward reaction:						
K_m (cis-aconitate)	18.5	3.4	µM		(Baumgart & Bott, 2011)	
K_{cat}^-	69.02		s^{-1}		(Baumgart & Bott, 2011)	
**acetyl-CoA:oxaloacetate C-acetyltransferase [(pro-S)-carboxymethyl-forming, ADP-phosphorylating]**						**77**
Stoichiometry: ATP(aq) + citrate(aq) + CoA(aq) ⇌ ADP(aq) + orthophosphate(aq) + acetyl-CoA(aq) + oxaloacetate(aq)						
K_{eq}	0.01334				eQuilibrator	
M	623000		g mol^{-1}		(Houston & Nimmo, 1984)	
Forward reaction:						
K_m (ATP)	222	28	µM		(Ranganathan et al., 1980)	
K_m (citrate)	40	10	µM		(Ranganathan et al., 1980)	
K_m (CoA)	2.6		µM		(Ranganathan et al., 1980)	
K_{cat}^+	141.2		s^{-1}		(Houston & Nimmo, 1984)	
**L-serine ammonia-lyase (pyruvate-forming)**						**18**
Stoichiometry: L-serine(aq) ⇌ pyruvate(aq) + NH3(aq)						
K_{eq}	$1.213 \cdot 10^5$				eQuilibrator	
M	48236		g mol^{-1}		(Gannon et al., 1977)	
Forward reaction:						
K_m (serine)	7000		µM		(Gannon et al., 1977)	
K_{cat}^+	530.6		s^{-1}		(Gannon et al., 1977)	

Table S2 (continued)

Enzyme name/ Parameter	Value	Uncer-tainty	Unit	Comment	Source	#
**malonate semialdehyde:NADP+ oxidoreductase (malonate semialdehyde-forming)**						**78**
Stoichiometry: malonyl-CoA(aq) + NADPH(aq) ⇌ malonate semialdehyde(aq) + NADP(aq) + CoA(aq)						
K_{eq}	$9.017 \cdot 10^{-3}$				eQuilibrator	
M	300000		g mol^{-1}		(Hügler et al., 2002)	
Forward reaction:						
K_m (malonyl-CoA)	30		µM		(Hügler et al., 2002)	
K_m (NADPH)	25		µM		(Hügler et al., 2002)	
K_{cat}^+	50		s^{-1}		(Hügler et al., 2002)	
**diphosphate---fructose-6-phosphate 1-phosphotransferase**						**79**
Stoichiometry: fructose-1,6-bisphosphate(aq) + phosphate(aq) ⇌ fructose-6-phosphate(aq) + pyrophosphate(aq)						
K_{eq}	0.2221				eQuilibrator	
M	90000		g mol^{-1}		(Reshetnikov et al., 2008)	
Forward reaction:						
K_m (fructose-1,6-bisphosphate)	328		µM		(Reshetnikov et al., 2008)	
K_m (phosphate)	8690		µM		(Reshetnikov et al., 2008)	
K_{cat}^+	13.5		s^{-1}		(Reshetnikov et al., 2008)	
Backward reaction:						
K_m (fructose-6-phosphate)	2270		µM		(Reshetnikov et al., 2008)	
K_m (pyrophosphate)	27		µM		(Reshetnikov et al., 2008)	
K_{cat}^-	11.4		s^{-1}		(Reshetnikov et al., 2008)	
**diphosphate---ribulose-5-phosphate 1-phosphotransferase**						**80**
Stoichiometry: ribulose-1,5-bisphosphate(aq) + phosphate(aq) ⇌ ribulose-5-phosphate(aq) + pyrophosphate(aq)						
K_{eq}	0.02098				eQuilibrator	
M	90000		g mol^{-1}		(Reshetnikov et al., 2008)	
Forward reaction:						
K_m (phosphate)	8690		µM		(Reshetnikov et al., 2008)	
K_{cat}^+	6.3		s^{-1}		(Reshetnikov et al., 2008)	
Backward reaction:						
K_m (pyrophosphate)	27		µM		(Reshetnikov et al., 2008)	
K_{cat}^-	4.5		s^{-1}		(Reshetnikov et al., 2008)	
**diphosphate---Sedoheptulose-7-phosphate 1-phosphotransferase**						**81**
Stoichiometry: sedoheptulose-bisphosphate(aq) + phosphate(aq) ⇌ sedoheptulose-7-phosphate(aq) + pyrophosphate(aq)						
K_{eq}	1.625				eQuilibrator	
M	90000		g mol^{-1}		(Reshetnikov et al., 2008)	
Forward reaction:						
K_m (phosphate)	8690		µM		(Reshetnikov et al., 2008)	
K_{cat}^+	33.75		s^{-1}		(Reshetnikov et al., 2008)	
Backward reaction:						
K_m (sedoheptulose-7-phosphate)	30		µM		(Reshetnikov et al., 2008)	
K_m (pyrophosphate)	27		µM		(Reshetnikov et al., 2008)	
K_{cat}^-	46.5		s^{-1}		(Reshetnikov et al., 2008)	
**ATP:pyruvate, water phosphotransferase (PEP synthase)**						**82**
Stoichiometry: ATP(aq) + pyruvate(aq) + H2O(l) ⇌ AMP(aq) + phosphoenolpyruvate(aq) + phosphate(aq)						
K_{eq}	2.410				eQuilibrator	
M	77000		g mol^{-1}		(NARINDRASORASAK & BRIDGER, 1977)	
Forward reaction:						
K_m (pyruvate)	83		µM		(Berman & Cohn, 1970)	
K_m (ATP)	24		µM		(Berman & Cohn, 1970)	
K_{cat}^+	10.27		s^{-1}		(NARINDRASORASAK & BRIDGER, 1977)	
**malate dehydrogenase (oxaloacetate-decarboxylating) (NADP+)**						**83**
Stoichiometry: malate(aq) + NADP(aq) ⇌ pyruvate(aq) + CO2(aq) + NADPH(aq)						
K_{eq}	0.004876				eQuilibrator	
M	330000		g mol^{-1}		(Ziegler, 1974)	
Forward reaction:						
K_m (malate)	700		µM		(Häusler et al., 1987)	
K_m (NADP)	15		µM		(Ziegler, 1974)	
K_{cat}^+	346.5		s^{-1}		(Häusler et al., 1987)	
Backward reaction:						
K_m (pyruvate)	3000		µM		(Ziegler, 1974)	
K_m (CO2)	1200		µM		(Häusler et al., 1987)	
K_m (NADPH)	45		µM		(Ziegler, 1974)	

Table S2 (continued)

**Enzyme name/ Parameter**	**Value**	**Uncer-tainty**	**Unit**	**Comment**	**Source**	**#**
**ATP:oxaloacetate carboxy-lyase (transphosphorylating; phosphoenolpyruvate-forming)**						**84**
Stoichiometry: oxaloacetate(aq) + ATP(aq) ⇌ phosphoenolpyruvate(aq) + ADP(aq) + CO2(aq)						
K_{eq}	0.01152				eQuilibrator	
M	70682		g mol^{-1}		UniProt	
Forward reaction:						
K_m (oxaloacetate)	156	2	µM		(Z. H. Chen et al., 2002)	
K_m (ATP)	25.7	3.5	µM		(Z. H. Chen et al., 2002)	
K_{cat}^+	47	6	s^{-1}		(Z. H. Chen et al., 2002)	
Backward reaction:						
K_m (phosphoenolpyruvate)	2300	250	µM		(Z. H. Chen et al., 2002)	
K_m (ADP)	36000	3300	µM		(Z. H. Chen et al., 2002)	
K_m (CO2)	55	18	µM		(Z. H. Chen et al., 2002)	
K_{cat}^-	22	2	s^{-1}		(Z. H. Chen et al., 2002)	
**Pyruvate:NAD+-oxidoreductase (decarboxylating, acceptor-acetylating) (pyruvate dehydrogenase complex)**						**85**
Stoichiometry: pyruvate(aq) + CoA(aq) + NAD+(aq) ⇌ acetyl-CoA(aq) + CO2(aq) + NADH(aq)						
K_{eq}	$1.732 \cdot 10^6$				eQuilibrator	
M	200000		g mol^{-1}		(SAUMWEBER et al., 1981)	
Forward reaction:						
K_m (pyruvate)	73		µM		(SAUMWEBER et al., 1981)	
K_m (NAD+)	70		µM		(Wang et al., 2010)Wa	
K_{cat}^+	5970		s^{-1}		(SAUMWEBER et al., 1981)	
**ATP:pyruvate 2-O-phosphotransferase**						**86**
Stoichiometry: phosphoenolpyruvate(aq) + ADP(aq) ⇌ pyruvate(aq) + ATP(aq)						
K_{eq}	$2.174 \cdot 10^3$				eQuilibrator	
M	185000		g mol^{-1}		(Abbe & Yamada, 1982)	
Forward reaction:						
K_m (phosphoenolpyruvate)	220		µM		(Abbe & Yamada, 1982)	
K_m (ADP)	390		µM		(Abbe & Yamada, 1982)	
K_{cat}^+	363.8		s^{-1}		(Abbe & Yamada, 1982)	
**isocitrate glyoxylate-lyase (succinate-forming)**						**87**
Stoichiometry: succinate(aq) + glyoxylate(aq) ⇌ isocitrate(aq)						
K_{eq}	43.42				eQuilibrator	
M	177000		g mol^{-1}		(MacKintosh & Nimmo, 1988)	
Forward reaction:						
K_m (succinate)	590		µM		(MacKintosh & Nimmo, 1988)	
K_m (glyoxylate)	130		µM		(MacKintosh & Nimmo, 1988)	
Backward reaction:						
K_m (isocitrate)	63	4	µM		(MacKintosh & Nimmo, 1988)	
K_{cat}^-	110.6		s^{-1}		(MacKintosh & Nimmo, 1988)	
**(S)-2-hydroxy carboxylate:oxygen 2-oxidoreductase**						**88**
Stoichiometry: glycolate(aq) + O2(aq) ⇌ glyoxylate(aq) + H2O2(aq)						
K_{eq}	$3.737 \cdot 10^{15}$				eQuilibrator	
M	40924		g mol^{-1}		(Pennati & Gadda, 2009)	
Forward reaction:						
K_m (glycolate)	200	10	µM		(Pennati & Gadda, 2009)	
K_m (O2)	440	20	µM		(Pennati & Gadda, 2009)	
K_{cat}^+	20	0.4	s^{-1}		(Pennati & Gadda, 2009)	
**3-phospho-D-glycerate carboxy-lyase (dimerizing; D-ribulose-1,5-bisphosphate-forming)**						**89**
Stoichiometry: ribulose-1,5-bisphosphate(aq) + O2(aq) ⇌ 3-phosphoglycerate(aq) + 2-phosphoglycolate(aq)						
K_{eq}	$2.389 \cdot 10^{92}$				eQuilibrator	
M	72268		g mol^{-1}		(Carmo-Silva et al., 2010)	
Forward reaction:						
K_m (ribulose-1,5-bisphosphate)	100		µM		(Carmo-Silva et al., 2010)	
K_m (O2)	341	33	µM		(Carmo-Silva et al., 2010)	
K_{cat}^+	0.95	0.04	s^{-1}		(Carmo-Silva et al., 2010)	
**2-phosphoglycolate phosphohydrolase**						**90**
Stoichiometry: 2-phosphoglycolate(aq) + H2O(l) ⇌ glycolate(aq) + phosphate(aq)						
K_{eq}	$1.030 \cdot 10^5$				eQuilibrator	
M	33012		g mol^{-1}		UniProt	
Forward reaction:						
K_m (2-phosphoglycolate)	26		µM		(Husic & Tolbert, 1984)	
K_{cat}^+	41.65		s^{-1}		(Husic & Tolbert, 1984)	

Table S2 (continued)

Enzyme name/ Parameter	Value	Uncer-tainty	Unit	Comment	Source	#
**(2R,3S)-beta-Hydroxyasparate hydro-lyase (Iminosuccinate forming)**						**91**
Stoichiometry: 3-hydroxyaspartate(aq) ⇌ iminosuccinate(aq) + H2O(l)						
K_{eq}	3.343				eQuilibrator	
M	34015		g mol^{-1}		(Schada von Borzyskowski et al., 2019), UniProt	
Forward reaction:						
K_m (3-hydroxyaspartate)	200	20	µM		(Schada von Borzyskowski et al., 2019)	
K_{cat}^+	35	1	s^{-1}		(Schada von Borzyskowski et al., 2019)	
**aspartate:NAD+ oxidoreductase**						**92**
Stoichiometry: iminosuccinate(aq) + NADH(aq) ⇌ aspartate(aq) + NAD+(aq)						
K_{eq}	$4.385 \cdot 10^{10}$				eQuilibrator	
M	33655		g mol^{-1}		(Schada von Borzyskowski et al., 2019), UniProt	
Forward reaction:						
K_m (iminosuccinate)	90	10	µM		(Schada von Borzyskowski et al., 2019)	
K_m (NADH)	20	3	µM		(Schada von Borzyskowski et al., 2019)	
K_{cat}^+	201	10	s^{-1}		(Schada von Borzyskowski et al., 2019)	
**L-alanine:3-oxopropanoate aminotransferase**						**93**
Stoichiometry: L-alanine(aq) + 3-oxopropanoate(aq) ⇌ pyruvate(aq) + beta-alanine(aq)						
K_{eq}	0.225				eQuilibrator	
M	48600		g mol^{-1}		UNIPROT	
Backward reaction:						
K_m (pyruvate)	14		mM		(Hayaishi et al., 1961)	
K_m (beta-alanine)	62		mM		(Hayaishi et al., 1961)	
K_{cat}^-	45.8		s^{-1}		(Hayaishi et al., 1961)	
**L-alanine 2,3-aminomutase [beta-alanine forming]**						**94**
Stoichiometry: L-alanine(aq) ⇌ beta-alanine(aq)						
K_{eq}	8.1				eQuilibrator	
M	224000			Tetrameric form	(D. Chen et al., 2000)	
Forward reaction:						
K_m (L-alanine)	8000	2200	µM	No data is available for alanine-specific variants of this enzyme; it was assumed that the parameters equal those for lysine when engineered	(D. Chen et al., 2000)	
K_{cat}^+	2.464		s^{-1}		(D. Chen et al., 2000)	
**6-phospho-D-gluconate:NADP+ 2-oxidoreductase (decarboxylating)**						**95**
Stoichiometry: 6-phospogluconate(aq) + NADP+ ⇌ ribulose-5-phosphate(aq) + CO2(aq) + NADPH						
K_{eq}	0.0156				eQuilibrator	
M	103000		g mol^{-1}	Homodimer	UNIPROT	
Forward reaction:						
K_m (6-phospogluconate)	35	2	µM		(Satanowski et al., 2020)	
K_m (NADP+)	12	3	µM		(Satanowski et al., 2020)	
K_{cat}^+	47.5	1	s^{-1}	Geometric mean	(Satanowski et al., 2020)	
Backward reaction:						
K_m (ribulose-5-phosphate)	2800	200	µM		(Satanowski et al., 2020)	
K_m (CO2)	900	100	µM		(Satanowski et al., 2020)	
K_m (NADPH)	1000	100	µM		(Satanowski et al., 2020)	
K_{cat}^-	5.24	0.2	s^{-1}	Geometric mean	(Satanowski et al., 2020)	
**6-phospho-D-gluconate hydro-lyase (2-dehydro-3-deoxy-6-phospho-D-gluconate-forming)**						**96**
Stoichiometry: 6-phospho-D-gluconate(aq) ⇌ 2-dehydro-3-deoxy-6-phospho-D-gluconate(aq) + H2O(l)						
K_{eq}	$3.2 \cdot 10^7$				eQuilibrator	
M	130000		g mol^{-1}	Homodimer		
Forward reaction:						
K_m (6-phospho-D-gluconate)	300		µM			
K_{cat}^+	133.5		s^{-1}			

Table S2 (continued)

**2-dehydro-3-deoxy-6-phospho-D-gluconate D-glyceraldehyde-3-phosphate-lyase (pyruvate-forming)**					**97**
Stoichiometry: 2-dehydro-3-deoxy-6-phospho-D-gluconate(aq) ⇌ D-glyceraldehyde 3-phosphate(aq) + pyruvate(aq)					
K_{eq}	$1.4 \cdot 10^{-3}$				eQuilibrator
M	72000			Homotrimer	(Hammerstedt et al., 1975)
Forward reaction:					
K_m (2-dehydro-3-deoxy-6-phospho-D-gluconate)	73		µM		(Hammerstedt et al., 1975)
K_{cat}^{+}	396		s^{-1}		(Hammerstedt et al., 1975)
**glyoxylate carboxy-lyase (dimerizing; 2-hydroxy-3-oxopropanoate-forming)**					**98**
Stoichiometry: 2 glyoxylate(aq) ⇌ 2-hydroxy-3-oxopropanoate(aq) + CO2(aq)					
K_{eq}	620				eQuilibrator
M	64800		$g\ mol^{-1}$		(Kaplun et al., 2008)
Forward reaction:					
K_m (glyoxylate)	900	400	µM		(Kaplun et al., 2008)
K_{cat}^{+}	18.9	2.7	s^{-1}		(Kaplun et al., 2008)
**D-glycerate:NAD(P)+ oxidoreductase**					**99**
Stoichiometry: D-glycerate(aq) + NAD+(aq) ⇌ 2-hydroxy-3-oxopropanoate(aq) + NADH(aq)					
K_{eq}	$6.1 \cdot 10^{-6}$				eQuilibrator
M	91000		$g\ mol^{-1}$	tetramer	(Gotto & Kornberg, 1961)
Forward reaction:					
K_m (glycerate)	400		µM		(Gotto & Kornberg, 1961)
Backward reaction:					
K_m (NADH)	20		µM		(Gotto & Kornberg, 1961)
K_m (2-hydroxy-3-oxopropanoate)	200		µM		(Gotto & Kornberg, 1961)
K_{cat}^{-}	243		s^{-1}		(Gotto & Kornberg, 1961)
**formaldehyde:NAD+ oxidoreductase**					**100**
Stoichiometry: formaldehyde(aq) + NAD+(aq) + H2O(l) ⇌ formate(aq) + NADH(aq)					
K_{eq}	$1.2 \cdot 10^{9}$				eQuilibrator
M	250000		$g\ mol^{-1}$		(Tate & Dalton, 1999)
Forward reaction:					
K_m (formaldehyde)	1200	100	µM		(Tate & Dalton, 1999)
K_{cat}^{+}	975		s^{-1}		(Tate & Dalton, 1999)
**formate:NADP+ oxidoreductase**					**101**
Stoichiometry: formate(aq) + NADP(aq) ⇌ CO2(aq) + NADPH(aq)					
K_{eq}	340				eQuilibrator
M	358000		$g\ mol^{-1}$		(Yu et al., 2017)
Forward reaction:					
K_m (formate)	310	6.5	µM	Assuming that a NADPH-dependent variant can be engineered with equal characteristics	(Yu et al., 2017)
K_m (NADP)	130	4.4	µM		(Yu et al., 2017)
K_{cat}^{+}	201	6.1	s^{-1}		(Yu et al., 2017)
Backward reaction:					
K_m (CO2)	2700	340	µM	Assuming that a NADPH-dependent variant can be engineered with equal characteristics	(Yu et al., 2017)
K_m (NADPH)	46	4.3	µM		(Yu et al., 2017)
K_{cat}^{-}	11	0.37	s^{-1}		(Yu et al., 2017)
**D-glucose-6-phosphate aldose-ketose-isomerase**					**102**
Stoichiometry: D-glucose-6-phosphate(aq) ⇌ D-fructose-6-phosphate(aq)					
K_{eq}	0.3				eQuilibrator
M	126000		$g\ mol^{-1}$		(Lin et al., 2009)
Forward reaction:					
K_{cat}^{+}	1000	50	s^{-1}		(Lin et al., 2009)
Backward reaction:					
K_m (D-fructose-6-phosphate)	37	3	µM		(Lin et al., 2009)
K_{cat}^{-}	650	50	s^{-1}		(Lin et al., 2009)
**D-glucose-6-phosphate:NADP+ 1-oxidoreductase**					**103**
Stoichiometry: D-glucose-6-phosphate(aq) + NADP+(aq) ⇌ 6-phospho-D-glucono-1,5-lactone(aq) + NADPH(aq)					
K_{eq}	15.7				eQuilibrator
M	60200		$g\ mol^{-1}$		(Jortzik et al., 2011)
Forward reaction:					
K_m (D-glucose-6-phosphate)	116	8.5	µM		(Jortzik et al., 2011)
K_m (NADP)	17.5	2.8	µM		(Jortzik et al., 2011)
K_{cat}^{+}	60.75	15	s^{-1}		(Jortzik et al., 2011)

Table S2 (continued)

**6-phospho-D-glucono-1,5-lactone lactonohydrolase**					**104**
Stoichiometry: D-glucono-1,5-lactone(aq) + H2O(l) ⇌ D-gluconate(aq)					
K_{eq}	$2.8 \cdot 10^4$				eQuilibrator
M	27547		g mol^{-1}	monomer	UNIPROT
Forward reaction:					
K_m (D-glucono-1,5-lactone)	242	48	µM		(Jortzik et al., 2011)
K_{cat}^{+}	489	45	s^{-1}		(Jortzik et al., 2011)
**NADPH:NAD+ oxidoreductase (Re/Si-specific)**					**105**
Stoichiometry: NADPH(aq) + NAD+(aq) + H+(in) ⇌ NADP+(aq) + NADH(aq) + H+(out)					
K_{eq}	$3.2 \cdot 10^{-3}$			Assuming transmembrane potential of 0.15 V	eQuilibrator
M	206692		g mol^{-1}	Dimer of heterodimer	UNIPROT
Backward reaction:					
K_m (NADP+)	13		µM		(Hu et al., 1995)
K_m (NADH)	3		µM		(Hu et al., 1995)
K_{cat}^{-}	46.5		s^{-1}	This was estimated from the 12-15 µmol/(mg min) in the reference. It should be a fair estimate since the difference in redox potential of the assay substrates imitates the driving force of the membrane potential	(Hu et al., 1995)
**methanol:ferricytochrome-c oxidoreductase**					**106**
Stoichiometry: methanol(aq) + 2 ferricytochrome cL(aq) ⇌ formaldehyde(aq) + 2 ferrocytochrome cL(aq)					
K_{eq}	$1.8 \cdot 10^6$			Calculated from redox potential +256 mV of the ferricytochrome/ ferrocytochrome redox couple	eQuilibrator (Williams et al., 2006)
M	129000		g mol^{-1}	Dimer of heterodimer	(Featherston et al., 2019)
Forward reaction:					
K_m (methanol)	22	3	µM		(Featherston et al., 2019)
K_m (ferricytochrome cL)	2.5	0.1	µM		(Featherston et al., 2019)
K_{cat}^{+}	2.19		s^{-1}	Geometric mean of different v(max) values	(Featherston et al., 2019)
**hydrogen:NAD+ oxidoreductase**					**107**
Stoichiometry: H2(aq) + NAD(aq) ⇌ NADH(aq)					
K_{eq}	$5.9 \cdot 10^6$				eQuilibrator
M	204 500		g mol^{-1}		(Schneider & Schlegel, 1976)
Forward reaction:					
K_m (NAD)	560		µM		(Schneider & Schlegel, 1976)
K_m (H2)	37		µM		(Schneider & Schlegel, 1976)
K_{cat}^{+}	185.6		s^{-1}	Geometric mean of different v(max) values	(Schneider & Schlegel, 1976)

2.2. Concentration bounds for metabolites

Table S3: Concentration ranges used for the Enzyme Cost Minimization algorithm

Compound name	Compound ID	KEGG entry	Lower bound, [M]	Upper bound, [M]	Comment
H2O	C_h2o	C00001	1	1	
ATP	C_atp	C00002	0.005	0.005	ATP/ADP ratio of 100
NAD	C_nad	C00003	0.001	0.001	
NADH	C_nadh	C00004	0.00001	0.0002	
NADPH	C_nadph	C00005	0.001	0.001	
NADP	C_nadp	C00006	1.00E-05	2.00E-04	
ADP	C_adp	C00008	0.00005	0.00005	ATP/ADP ratio of 100
phosphate	C_orthophosphate	C00009	0.01	0.01	
CoA	C_coa	C00010	0.001	0.001	
CO2	C_co2	C00011	1.00E-05	1.00E-05	Order of magnitude of air saturated water (~350-400 ppm CO2)
pyrophosphate	C_diphosphate	C00013	0.001	0.001	
NH3	C_nh3	C00014	0.0001	0.01	
AMP	C_amp	C00020	0.0005	0.0005	
pyruvate	C_pyruvate	C00022	1.00E-06	0.01	
acetyl-CoA	C_acetyl_coa	C00024	1.00E-06	0.01	
2-oxoglutarate	C_2_oxoglutarate	C00026	1.00E-06	0.01	
oxaloacetate	C_oxaloacetate	C00036	1.00E-06	0.01	
glycine	C_glycine	C00037	1.00E-06	0.01	
succinate	C_succinate	C00042	1.00E-06	0.01	
glyoxylate	C_glyoxylate	C00048	1.00E-06	0.01	
aspartate	C_l_aspartate	C00049	1.00E-06	0.01	
formate	C_formate	C00058	1.00E-06	0.01	
serine	C_l_serine	C00065	1.00E-06	0.01	
formaldehyde	C_formaldehyde	C00067	1.00E-09	0.01	
phosphoenolpyruvate	C_phosphoenolpyruvate	C00074	1.00E-06	0.01	
malonyl-CoA	C_malonyl_coa	C00083	1.00E-06	0.01	
fructose-6-phosphate	C_d_fructose_6_phosphoric_acid	C00085	1.00E-06	0.01	
succinyl-CoA	C_succinyl_coa	C00091	1.00E-06	0.01	
propanoyl-CoA	C_propanoyl_coa	C00100	1.00E-06	0.01	
tetrahydrofolate	C_tetrahydrofolate	C00101	1.00E-06	0.01	
glycerone phosphate	C_glycerone_phosphate	C00111	1.00E-06	0.01	
ribose-5-phosphate	C_d_ribose_5_phosphate	C00117	1.00E-06	0.01	
glyceraldehyde-3-phosphate	C_d_glyceraldehyde_3_phosphate	C00118	1.00E-06	0.01	
fumarate	C_fumarate	C00122	1.00E-06	0.01	
methanol	C_methanol	C00132	1.00E-06	0.1	Methanol can be used at high concentrations in a bioreactor
5,10-methylenetetrahydrofolate	C_510_methylenetetrahydrofolate	C00143	1.00E-06	0.01	
malate	C_s_malate	C00149	1.00E-06	0.01	
citrate	C_citrate	C00158	1.00E-06	0.01	
hydroxypyruvate	C_hydroxypyruvate	C00168	1.00E-06	0.01	

Table S3 (continued)

Compound name	Compound ID	KEGG entry	Lower bound, [M]	Upper bound, [M]	Comment
3-phosphoglycerate	C_3_phospho_d_glycerate	C00197	1.00E-06	0.01	
ribulose-5-phosphate	C_d_ribulose_5_phosphate	C00199	1.00E-06	0.01	
malonate semialdehyde	C_3_oxopropanoate	C00222	1.00E-06	0.01	
xylulose-5-phosphate	C_d_xylulose_5_phosphate	C00231	1.00E-06	0.01	
succinate semialdehyde	C_succinate_semialdehyde	C00232	1.00E-06	0.01	
10-formyltetrahydrofolate	C_10_formyltetrahydrofolate	C00234	1.00E-06	0.01	
1,3-bisphosphoglycerate	C_3_phospho_d_glyceroyl_phosphate	C00236	1.00E-06	0.01	
lipoamide	C_lipoamide	C00248	1.00E-08	0.0005	Bounds for lipoamide are lower as it is supposed to be attached to the H-protein
glycerate	C_d_glycerate	C00258	1.00E-06	0.01	
erythrose-4-phosphate	C_d_erythrose_4_phosphate	C00279	1.00E-06	0.01	
isocitrate	C_isocitrate	C00311	1.00E-06	0.01	
acetoacetyl-CoA	C_acetoacetyl_coa	C00332	1.00E-06	0.01	
fructose-1,6-bisphosphate	C_d_fructose_16_bisphosphate	C00354	1.00E-06	0.01	
ubiquinol	C_ubiquinol	C00390	1.00E-07	1.00E-04	Quinones are located in the inner membrane and thus the concentration is assumed to be lower
ubiquinone	C_ubiquinone	C00399	1.00E-07	1.00E-04	
cis-aconitate	C_cis_aconitate	C00417	1.00E-06	0.01	
5,10-methenyltetrahydrofolate	C_510_methenyltetrahydrofolate	C00445	1.00E-06	0.01	
seduheptulose-1,7-bisphosphate	C_d_altro_heptulose_17_biphosphate	C00447	1.00E-06	0.01	
benzoquinone	C_p_benzoquinone	C00472	1.00E-06	0.01	
hydroquinone	C_hydroquinone	C00530	1.00E-06	0.01	
dihydrolipoamide	C_dihydrolipoamide	C00579	1.00E-08	0.0005	Bounds for lipoamide are lower as it is supposed to be attached to the H-protein
2-phosphoglycerate	C_2_phospho_d_glycerate	C00631	1.00E-06	0.01	
S-methylmalonyl-CoA	C_s_methylmalonyl_coa	C00683	1.00E-06	0.01	
formyl-CoA	C_formyl_coa	C00798	1.00E-06	0.01	
menaquinone	C_menaquinone	C00828	1.00E-07	1.00E-04	Quinones are located in the inner membrane and thus the concentration is assumed to be lower
crotonyl-CoA	C_crotonoyl_coa	C00877	1.00E-06	0.01	
acrylyl-CoA	C_propenoyl_coa	C00894	1.00E-06	0.01	
4-hydroxybutanoate	C_4_hydroxybutanoic_acid	C00989	1.00E-06	0.01	
citramalyl-CoA	C_citramalyl_coa	C01011	1.00E-06	0.01	
3-hydroxypropanoate	C_3_hydroxypropanoate	C01013	1.00E-06	0.01	
3S-hydroxybutanoyl-CoA	C_s_3_hydroxybutanoyl_coa	C01144	1.00E-06	0.01	
ribulose-1,5-bisphosphate	C_d_ribulose_15_biphosphate	C01182	1.00E-06	0.01	
R-Methylmalonyl-CoA	C_r_methylmalonyl_coa	C01213	1.00E-06	0.01	
$HCO3^{-}$	C_hco3_	C01353	1.00E-06	0.00026	Order of magnitude of air saturated water (~350-400 ppm CO2)
2-hydroxyglutarate	C_2_hydroxyglutarate	C02630	1.00E-06	0.01	

Table S3 (continued)

Compound name	Compound ID	KEGG entry	Lower bound, [M]	Upper bound, [M]	Comment
2-hydroxyglutaryl-CoA	C_2_hydroxyglutaryl_coa	C03058	1.00E-06	0.01	
β-hydroxyasparatate	C_erythro_3_hydroxy_ls_aspartate	C03961	1.00E-06	0.01	
malyl-CoA	C_3s_3_carboxy_3_hydroxypropanoyl_coa	C04348	1.00E-06	0.01	
seduheptulose-7-phosphate	C_d_sedoheptulose_7_phosphate	C05382	1.00E-06	0.01	
3-hydroxypropanoyl-CoA	C_3_hydroxypropionyl_coa	C05668	1.00E-06	0.01	
menaquinol	C_menaquinol	C05819	1.00E-07	1.00E-04	Quinones are located in the inner membrane and thus the concentration is assumed to be lower
iminoaspartate	C_iminoaspartate	C05840	1.00E-06	0.01	
hexulose-6-phosphate	C_d_arabino_6_phospho_hex_3_ulose	C06019	1.00E-06	0.01	
β-methylmalyl-CoA	C_l_erythro_3_methylmalyl_coa	C06027	1.00E-06	0.01	
mesaconyl-C1-CoA	C_mesaconyl_coa	C06028	1.00E-06	0.01	
4-hydroxybutanoyl-CoA	C_4_hydroxybutyryl_coa	C11062	1.00E-06	0.01	
S-ethylmalonyl-CoA	C_s_ethylmalonyl_coa	C18026	1.00E-06	0.01	
mesaconyl-C4-CoA	C_mesaconyl_c4_coa	C18323	1.00E-06	0.01	
methylsuccinyl-CoA	C_methylsuccinyl_coa	C18324	1.00E-06	0.01	
R-ethylmalonyl-CoA	C_r_ethylmalonyl_coa	C20238	1.00E-06	0.01	
S(8)-aminomethyldihydrolipoamide	C_s8_aminomethyldihydrolipoamide	C90640	1.00E-08	0.0005	Bounds for lipoamide are lower as it is supposed to be attached to the H-protein
O2	C_oxygen	C00007	0.000273	0.000273	
H2O2	C_hydrogen_peroxide	C00027	1.00E-06	0.01	
glycolate	C_glycolate	C00160	1.00E-06	0.01	
2-phosphoglycolate	C_2_phosphoglycolate	C00988	1.00E-06	0.01	
L-alanine	C_l_alanine	C00041	1.00E-06	0.01	
B-alanine	C_beta_alanine	C00099	1.00E-06	0.01	
6-phosphogluconate	C_6_phosphogluconate	C00345	1.00E-06	0.01	
2-dehydro-3-deoxy-6-phosphogluconate	C_2_dehydro_3_deoxy_6_phosphoglucona te	C04442	1.00E-06	0.01	
2-hydroxy-3-oxopropanoate	C_2_hydroxy_3_oxopropanoate	C01146	1.00E-06	0.01	
D-glucose-6-phosphate	C_d_glucose_6_phosphoric_acid	C00092	1.00E-06	0.01	
D-6-phosphoglucono-1,5-lactone	C_d_6_phospho_D_glucono_15_lactone	C01236	1.00E-06	0.01	
ferricytochrome cL	C_ferricytochrome_cL	C18233	1.00E-07	1.00E-04	Lower as standard since this is a protein associated cofactor
ferrocytochrome cL	C_ferrocytochrome_cL	C18234	1.00E-07	1.00E-04	
H2	C_h2	C00282	1.00E-07	1.00E-04	Upper bound is in the order of saturated 10% H2 atmosphere

3. Correction of the kinetics of the C5-carboxylic acid subnetwork

For some reactions in the data structure, estimates of the equilibrium constants that were derived from eQuilibrator had a very high uncertainty at the time the data was collected. This was especially pronounced for the reactions that formed or cleaved C5-dicarboxylic acids, for instance the reaction of β-methylmalyl-CoA lyase:

β-methylmalyl-CoA ⇌ propanoyl-CoA + glyoxylate

eQuilibrator predicted a free energy of $\Delta_r G^0$ = 15.9 ± 16.4 kJ/mol for this reaction translating to a standard deviation of a factor of around 750 in the equilibrium constant, which has a considerable impact on the directionality of the reactions. The same was observed for the crotonyl-CoA reductase/carboxylase ($\Delta_r G'^\circ$ = -43±17 kJ/mol), the β-methylmalyl-CoA lyase ($\Delta_r G'^\circ$ = +16±16 kJ/mol) and citramalyl-CoA lyase ($\Delta_r G'^\circ$ = +11±15 kJ/mol) as shown in Figure S11. These reactions correspond to the in- and outlet of the C5 dicarboxylic acid platform which are thus coupled by Wegscheider conditions. For these reactions, the equilibrium constants were adjusted by using the kinetic information that was available for these reaction: As the equilibrium constant is mathematically related to the other parameters (compare Equation S4 in the main manuscript), this could be done by estimating a new equilibrium constant for β-methylmalyl-CoA lyase by Parameter Balancing (a complete set of parameters was determined for this reaction (Erb et al., 2010)). An equilibrium constant of K_{eq} = 11.8±1.5 (geometric standard deviation) was calculated by the algorithm, corresponding to a standard free reaction energy of $\Delta_r G'^\circ$ = +6.1±1.0 kJ/mol. Based on the measured kinetic data, this reaction is thus considerably more exergonic than predicted by eQuilibrator which is in favor of the thermodynamics for the ethylmalonyl-CoA pathway and the CETCH Cycle that use this reaction for β-methylmalyl-CoA cleavage. Next, the other reactions of C5-carboxylic acids with high uncertainties were corrected by the relative change of the new estimate for the equilibrium constant compared to the original constant of β-methylmalyl-CoA lyase. This was necessary to fulfil Wegscheider conditions that were otherwise violated by the change of the equilibrium constant of β-methylmalyl-CoA lyase. Fortunately, the C5 dicarboxylic acid platform is enclosed by a small set of reactions, so it sufficed to adjust the free energies and equilibrium constants of crotonyl-CoA reductase/carboxylase (new $\Delta_r G'^\circ$ = -33 kJ/mol) and citramalyl-CoA lyase (new $\Delta_r G'^\circ$ = +1 kJ/mol) as described in Material and Methods. The original uncertainties in these reactions were actually higher than the change applied to them which justifies the correction. An alternative approach would be to feed the new balanced equilibrium constant of β-methylmalyl-CoA lyase with the eQuilibrator API or another component contribution method which should result in a similar estimation for the remaining equilibrium constants. For the crotonyl-CoA reductase/carboxylase, the change makes hardly any difference since the reaction stays highly exergonic. The adjustment for citramalyl-CoA lyase change the thermodynamics in favor of the reductive citramalyl-CoA cycle.

Figure S11 also illustrates the thermodynamics of the conversion of propionyl-CoA and glyoxylate to pyruvate and acetyl-CoA in the Hydroxypropionate Bicycle which is exergonic overall. It is also clear that when comparing the two exits of the C5 dicarboxylic acid platform, the route towards acetyl-CoA and pyruvate is more exergonic in total which gives the rCCC a slight edge over the CETCH Cycle on a thermodynamic level.

By just changing the equilibrium constants of the reactions with high uncertainties, the Haldane relationship in Equation S4 did not hold anymore, however, since only the left side of the equation was changed. A conservative way to adjust the right side of Equation S4 is to modify those parameters that are estimated instead of the values that were actually experimentally determined. As an example: If the forward reaction rate k_{cat}^{+} of a certain reaction is known from experimental data and the

equilibrium constant is corrected to be twice as high as estimated by eQuilibrator, the Haldane relationship can be fulfilled by dividing the backward reaction rate k_{cat}^{-} by 2.

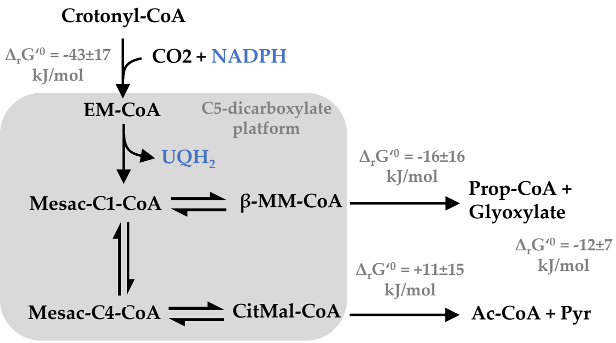

Figure S11: Schematic overview of the C5 dicarboxylic acid platform. The free reaction energies as predicted by eQuilibrator (pH 7.5, I = 0.25 M) are indicated for the influx and efflux reactions of the platform. Note the high uncertainties of the free energies. Additionally, the free energy of the hypothetical conversion of propionyl-CoA and glyoxylate to pyruvate and acetyl-CoA is indicated (dashed reaction arrow). Abbreviations: EM-CoA, ethylmalonyl-CoA; Mesac-C1-CoA, mesaconyl-C1-CoA; β-MM-CoA, β-methylmalyl-CoA; Prop-CoA, propanoyl-CoA; Mesac-C4-CoA, mesaconyl-C4-CoA; CitMal-CoA, citramalyl-CoA; Ac-CoA, acetyl-CoA; Pyr, pyruvate.

All reactions that were affected by the change of the equilibrium constant of β-methylmalyl-CoA lyase, were adjusted by the before mentioned approach. After the corrections, the reactions again fulfilled Wegscheider conditions and Haldane relationships.

4. Background information on parameter sampling with Parameter Balancing

The SBtab files with the collected data were used as the input for the Parameter Balancing algorithm to estimate the missing kinetic parameters. To this end, a kinetic model for the reaction has to be defined. Since the exact reaction mechanisms are not known for every enzyme, a modular rate law for the flux v was used that is flexible enough to give good estimations (Liebermeister et al., 2010):

$$\mathrm{v} = E\,\frac{k_{cat}^{+}\prod\frac{S_i}{K_{S_i}} - k_{cat}^{-}\prod\frac{P_j}{K_{P_j}}}{\prod\left(\frac{S_i}{K_{S_i}}+1\right)+\prod\left(\frac{P_j}{K_{P_j}}+1\right)-1} \qquad \text{(Equation S1)}$$

with E: enzyme concentration, S_i: concentration of substrate *i*, P_j: concentration of product *j*, K_{S_i}: half-saturation constant of substrate *i*, K_{P_j}: half-saturation constant of product *j*, k_{cat}^{+}: forward rate constant, k_{cat}^{-}: backward rate constant

Following the derivation by (Noor et al., 2016), this can be refactored to:

$$\mathrm{v} = \underbrace{E\,k_{cat}^{+}}_{v_{max}}\underbrace{\left(1-\frac{k_{cat}^{-}\prod\frac{P_j}{K_{P_j}}}{k_{cat}^{+}\prod\frac{S_i}{K_{S_i}}}\right)}_{\eta_{rev}}\underbrace{\frac{\prod\frac{S_i}{K_{S_i}}}{\prod\left(\frac{S_i}{K_{S_i}}+1\right)+\prod\left(\frac{P_j}{K_{P_j}}+1\right)-1}}_{\eta_{kin}} \qquad \text{(Equation S2)}$$

v_{max} is the maximal velocity of the reaction or the "capacity" of the enzyme that is decreased by the reversibility factor η_{rev} and the kinetic factor η_{kin}. These factors are numbers between 0 and 1. This holds for positive fluxes – for negative fluxes, the equation is refactored to:

$$\mathrm{v} = \underbrace{-E\,k_{cat}^{-}}_{v_{max}}\underbrace{\left(1-\frac{k_{cat}^{+}\prod\frac{S_i}{K_{S_i}}}{k_{cat}^{-}\prod\frac{P_j}{K_{P_j}}}\right)}_{\eta_{rev}}\underbrace{\frac{\prod\frac{P_j}{K_{P_j}}}{\prod\left(\frac{S_i}{K_{S_i}}+1\right)+\prod\left(\frac{P_j}{K_{P_j}}+1\right)-1}}_{\eta_{kin}} \qquad \text{(Equation S3)}$$

The conditional factors give information on why reactions are not running at their maximum capacity: A low reversibility factor η_{rev} indicates that the thermodynamics are limiting the reaction velocity, e.g. because of a low equilibrium constant. A low kinetic factor η_{kin} indicates a kinetic limitation because of substrate or product saturation (Noor et al., 2016). This kinetic law was used as a template to estimate the remaining parameters with the Parameter Balancing algorithm. After parameter balancing, the resulting parameters should fulfil the Haldane relationship which can be formulated as follows (Noor et al., 2016):

$$K_{eq} = \frac{k_{cat}^{+}\prod_i (K_i^P)^{m_i^P}}{k_{cat}^{-}\prod_i (K_i^S)^{m_i^S}} \qquad \text{(Equation S4)}$$

with K_{eq}: equilibrium constant, k_{cat}: forward and backward rate constants, K_i^P: Michaelis constant of product i, m_i^P: stoichiometric factor of product i, K_i^S: Michaelis constant of substrate i, m_i^S: stoichiometric factor of substrate i.

5. Summary of pathway stoichiometries

Table S4: Total stoichiometry and free Gibbs energy of CO_2- and C1-fixing pathways for the production of acetyl-CoA/glyoxylate

Originating Pathway [e]	Stoichiometry	$\Delta_rG'^0$, kJ/mol
**CBB cycle variants**		
CBB | GAPtoOAA | C3to2C2(4HB)(T)| -PyrC	5 ATP + 3.5 NADPH + CoA + 1.5 CO_2 + 0.5 HCO_3^- + 0.5 MQH_2 + H^+_{in} → 3.5 $NADP^+$ + 5 ADP + 5 P_i + acetyl-CoA + 0.5 MQ + H^+_{out}	-108.7 ± 12.8 [a]
CBB | Photorespiration | GAPtoOAA | C3to2C2(4HB)(T) | -PyrC	6.35 ATP + 0.15 NADH + 4.25 NADPH + CoA + 1.5 CO_2 + 0.5 HCO_3^- + 0.5 MQH_2 + 0.6 O_2 + H^+_{in} → 0.15 NAD^+ + 4.25 $NADP^+$ + 6.35 ADP + 6.35 P_i + acetyl-CoA + 0.5 MQ + 0.3 H_2O_2 + H^+_{out}	-322 ± 14 [a]
CBB | GAPtoOAA | PyrKin | PEPCarb | PyrDehCompl	7 ATP + NAD^+ + 5 NADPH + CoA + 2 CO_2 → NADH + 5 $NADP^+$ + 7 ADP + 7 P_i + acetyl-CoA	-205.6 ± 13.0
**HP/HB (Crenarchaea) cycle variants**		
HB/HP-Cren	4 ATP + NAD^+ + 5 NADPH + CoA + 2 HCO_3^- → NADH + 5 $NADP^+$ + 2 ADP + 2 P_i + 2 PP_i + 2 AMP + acetyl-CoA	-143.7 ± 12.8
**HP/HB (Thaumarchaea) cycle variants**		
HB/HP-Thaum	4 ATP + NAD+ + 5 NADPH + CoA + 2 HCO_3^- → NADH + 5 $NADP^+$ + 4 ADP + 4 P_i + acetyl-CoA	-116.6 ± 12.6
**HP bicycle variants**		
HP-Bicycle | C3to2C2(4HB)(T)	4 ATP + 4 NADPH + CoA + 2 HCO_3^- → 4 $NADP^+$ + 3 ADP + 3 P_i + PP_i + AMP + acetyl-CoA	-130.2 ± 12.6
**Serine cycle variants**		
Serine-Cycle	3 ATP + 2 NADH + NADPH + CoA + formate + HCO_3^- → 2 NAD^+ + $NADP^+$ + 3 ADP + 3 P_i + acetyl-CoA	-101.4 ± 7.4
Serine-Cycle (w/o SHMT)	3 ATP + 2 NADH + NADPH + CoA + formate + HCO_3^- → 2 NAD^+ + $NADP^+$ + 3 ADP + 3 P_i + acetyl-CoA	-101.4 ± 7.4
**RuMP cycle variants**		
RuMP-Cycle | GAPtoOAA | C3to2C2(4HB)(T) | PyrC	1.5 ATP + 1.5 NAD^+ + 0.5 NADPH + CoA + 1.5 methanol + 0.5 HCO_3^- + 0.5 MQH_2 + H^+_{in} → 1.5 NADH + 0.5 $NADP^+$ + 1.5 ADP + 1.5 P_i + acetyl-CoA + 0.5 MQ + H^+_{out}	-58.4± 9.9
RuMP-Cycle(Formate/THF) | GAPtoOAA | C3to2C2(4HB)(T) | -PyrC	3 ATP + 2 NADPH + CoA + 1.5 formate + 0.5 HCO_3^- + 0.5 MQH_2 + H^+_{in} → 2 $NADP^+$ + 3 ADP + 3 P_i + acetyl-CoA + 0.5 MQ + H^+_{out}	-71.0 ± 6.7 [a]
**rCCC variants**		
rCCC	3 ATP + NADH + 3 NADPH + CoA + CO_2 + UQ + HCO_3^- + MQH_2 + 2 H^+_{in} → NAD^+ + 3 $NADP^+$ + 3 ADP + 3 P_i + acetyl-CoA + UQH_2 + MQ + 2 H^+_{out}	-94.2 ± 14.8 [a]
**MOG cycle variants**		
MOG(PyrC)	3 ATP + NADH + NADPH + 2 HCO_3^- → → NAD^+ + $NADP^+$ + 3 ADP + 3 P_i + glyoxylate	-12 ± 12
**CETCH cycle variants**		
CETCH | b-OH-AspCycle | C3to2C2(4HB)(T)',0.5,'-PyrC	3 ATP + 0.5 NADH + 4 NADPH + CoA + CO_2 + UQ + HCO_3^- + 0.5 MQH_2 + H^+_{in} → 0.5 NAD^+ + 4 $NADP^+$ + 3 ADP + 3 P_i + Acetyl-CoA + UQH_2 + 0.5 MQ + H^+_{out}	-131.7 ± 14.1 [a]
CETCH | b-OH-AspCycle | MCG-like-cycle	3 ATP + 2 NADH + 3 NADPH + CoA + CO_2 + UQ + HCO_3^- → 2 NAD^+ + 3 $NADP^+$ + 3 ADP + 3 P_i + acetyl-CoA + UQH_2	-169.3 ± 13.9
CETCH | RevGlyoxShunt	3 ATP + NADH + 3 NADPH + CoA + CO_2 + UQ + HCO_3^- + MQH_2 + 2 H^+_{in} → NAD^+ + 3 $NADP^+$ + 3 ADP + 3 P_i + acetyl-CoA + UQH_2 + MQ + 2 H^+_{out}	-94.2 ± 14.8 [a]
**rGlyP variants**		
rGlyP | PyrDehCompl	2 ATP + 2 NADPH + CoA + 2 formate → 2 $NADP^+$ + 2 ADP + 2 P_i + acetyl-CoA	-86.2 ± 6.3
rGlyP (w/o SHMT) | PyrDehCompl	2 ATP + 2 NADPH + CoA + 2 formate → 2 $NADP^+$ + 2 ADP + 2 P_i + acetyl-CoA	-86.2 ± 6.3
**2-HG-rTCA cycle variants**		
2-HG-rTCA	2 ATP + 2 NADPH + CoA + CO_2 + formate + MQH_2 + 2 H^+_{in} → 2 $NADP^+$ + 2 ADP + 2 P_i + acetyl-CoA + MQ + 2 H^+_{out}	+3.4 ± 9.3 [a]

[a] the free energy was calculated assuming a transmembrane potential of -150 mV

Table S5: Total stoichiometry and free Gibbs energy of CO2- and C1-fixing pathways for the production of glyceraldeyhde-3-phosphate

**Originating Pathway** [e]	**Stoichiometry**	**$\Delta_r G'^0$, kJ/mol**
**CBB cycle variants**		
CBB	9 ATP + 6 NADPH + 3 CO_2 → 6 $NADP^+$ + 9 ADP + 8 Pi + glyceraldehyde-3-phosphate	-128.6 ± 18.8
CBB(Photorespiration)	11.7 ATP + 0.3 NADH + 7.5 NADPH + 3 CO_2 + 1.20 O_2 → 0.3 NAD^+ + 7.5 $NADP^+$ + 11.7 ADP + 10.7 P_i + glyceraldehyde-3-phosphate + 0.6 H_2O_2	-556 ± 21
CBB(PTS)	7 ATP + 6 NADPH + 3 CO_2 → 6 $NADP^+$ + 7 ADP + 6 P_i + glyceraldehyde-3-phosphate	-69.3 ± 18.5
**HP/HB (Crenarchaea) cycle variants**		
HB/HP-Cren | rCC-AcCoAtoPyr | PyrC | OAAtoGAP	7 ATP + NAD^+ + 8 NADPH + UQ + 3 HCO_3^- → NADH + 8 $NADP^+$ + 5 ADP + 4 P_i + 2 PP_i + 2 AMP + glyceraldehyde-3-phosphate + UQH_2	-178.6 ± 20.0
HB/HP-Cren | AcCoAtoOAA(HP)(AMP) | OAAtoGA	8 ATP + 2 NAD^+ + 9 NADPH + MQ + 4 HCO_3^- + 2 H^+_{out} → 2 NADH + 9 $NADP^+$ + 5 ADP + 4 P_i + CO_2 + 3 PP_i + 3 AMP + glyceraldehyde-3-phosphate + MQH_2 + 2 H^+_{in}	-214.8 ± 20.4 [a]
**HP/HB (Thaumarchaea) cycle variants**		
HB/HP-Thaum | rCC-AcCoAtoPyr | PyrC | OAAtoGAP	7 ATP + NAD^+ + 8 NADPH + UQ + 3 HCO_3^- → NADH + 8 $NADP^+$ + 7 ADP + 6 P_i + glyceraldehyde-3-phosphate + UQH_2	-151.6 ± 19.8
HB/HP-Thaum | AcCoAtoOAA(HP)(ADP) | OAAtoGAP	8 ATP + 2 NAD^+ + 9 NADPH + MQ + 4 HCO_3^- + 2 H^+_{out} → 2 NADH + 9 $NADP^+$ + 8 ADP + 7 P_i + CO_2 + glyceraldehyde-3-phosphate + MQH_2 + 2 H^+_{in}	-174.2 ± 19.8 [a]
**HP bicycle variants**		
HP-Bicycle | PyrC | OAAtoGAP	8 ATP + 7 NADPH + MQ + 4 HCO_3^- + 2 H^+_{out} → 7 $NADP^+$ + 6 ADP + 5 P_i + CO2 + 2 PP_i + 2 AMP + glyceraldehyde-3-phosphate + MQH_2 + 2 H^+_{in}	-201.1 ± 20.0 [a]
HP-Bicycle(ADP) | PyrC | OAAtoGAP	8 ATP + 7 NADPH + MQ + 4 HCO3- + 2 H^+_{out} → 7 NADP+ + 8 ADP + 7 P_i + CO_2 + glyceraldehyde-3-phosphate + MQH_2 + 2 H^+_{in}	-174.1 ± 19.8 [a]
HP-Bicycle(glyox) | b-OH-AspCycle | OAAtoGAP	8 ATP + NADH + 7 NADPH + 2 MQ + 4 HCO_3^- + 4 H^+_{out} → NAD+ + 7 NADP+ + 6 ADP + 5 P_i + CO_2 + 2 PP_i + 2 AMP + glyceraldehyde-3-phosphate + 2 MQH_2 + 4 H^+_{in}	-276.4 ± 22.5 [a]
HP-Bicycle(glyox)(ADP) | b-OH-AspCycle | OAAtoGAP	8 ATP + NADH + 7 NADPH + 2 MQ + 4 HCO_3^- + 4 H^+_{out} → NAD^+ + 7 $NADP^+$ + 8 ADP + 7 P_i + CO_2 + glyceraldehyde-3-phosphate + 2 MQH_2 + 4 H^+_{in}	-249.3 ± 22.4 [a]
**Serine cycle variants**		
Serine-Cycle (w/o SHMT) | EtMalOAAGlyox | GlyoxToOAA(Ser) | OAAtoGAP	6 ATP + 2 NADH + 3.5 NADPH + 1.5 Formate + 0.5 UQ + 0.5 MQ + 2 HCO_3^- + H^+_{out} → 2 NAD^+ + 3.5 $NADP^+$ + 6 ADP + 5 P_i + 0.5 CO_2 + glyceraldehyde-3-phosphate + 0.5 UQH_2 + 0.5 MQH_2 + H^+_{in}	-140.0 ± 11.5 [a]
Serine-Cycle | EtMalOAAGlyox | GlyoxToOAA(Ser) | OAAtoGAP	6 ATP + 2 NADH + 3.5 NADPH + 1.5 Formate + 0.5 UQ + 0.5 MQ + 2 HCO_3^- + H^+_{out} → 2 NAD^+ + 3.5 $NADP^+$ + 6 ADP + 5 P_i + 0.5 CO_2 + glyceraldehyde-3-phosphate + 0.5 UQH_2 + 0.5 MQH_2 + H^+_{in}	-140.0 ± 11.5 [a]
**RuMP cycle variants**		
RuMP-Cycle	2 ATP + 3 NAD^+ + 3 Methanol → 3 NADH + 2 ADP + P_i + glyceraldehyde-3-phosphate	-28.0 ± 17.7
RuMP-Cycle(Formate/THF)	5 ATP + 3 NADPH + 3 Formate → 3 $NADP^+$ + 5 ADP + 4 P_i + glyceraldehyde-3-phosphate	-53.3 ± 8.4
**rCCC variants**		
rCCC | rCC-AcCoAtoPyr | PyrC | OAAtoGAP	6 ATP + NADH + 6 NADPH + CO_2 + 2 UQ + 2 HCO_3^- + MQH_2 + 2 H^+_{in} → NAD^+ + 6 $NADP^+$ + 6 ADP + 5 P_i + glyceraldehyde-3-phosphate + 2 UQH_2 + MQ + 2 H^+_{out}	-129.1 ± 8.4 [a]
**GED cycle variants**		
GED cycle	6 ATP + 6 NADPH + 3 CO_2 → 6 $NADP^+$ + 3 ADP + 8 P_i + 3 AMP + glyceraldehyde 3-phosphate	-129.6 ± 18.7
**CETCH cycle variants**		
CETCH | b-OH-AspCycle | OAAtoGAP	6 ATP + NADH + 7 NADPH + CO_2 + 2 UQ + 2 HCO_3^- → NAD^+ + 7 $NADP^+$ + 6 ADP + 5 P_i + glyceraldehyde-3-phosphate + 2 UQH_2	-204.2 ± 22.2
CETCH | GlyoxToOAA-HPbicycle(AMP) | OAAtoGAP	7 ATP + 7 NADPH + UQ + 3 HCO_3^-→ 7 $NADP^+$ + 6 ADP + 5 P_i + PP_i + AMP + glyceraldehyde-3-phosphate + UQH_2	-165.1 ± 19.8
**rGlyP variants**		
rGlyP | PyrC | OAAtoGAP	5 ATP + NADH + 3 NADPH + 2 Formate + HCO_3^- → NAD^+ + 3 $NADP^+$ + 5 ADP + 4 P_i + glyceraldehyde-3-phosphate	-38.9 ± 8.7
rGlyP (w/o SHMT) | PyrC | OAAtoGAP	5 ATP + NADH + 3 NADPH + 2 Formate + HCO_3^- → NAD^+ + 3 $NADP^+$ + 5 ADP + 4 P_i + glyceraldehyde-3-phosphate	-38.9 ± 8.7

Table S5 (continued)

**2-HG-rTCA cycle variants**		
2-HG-rTCA | rCC-AcCoAtoPyr | PyrC | OAAtoGAP	5 ATP + 5 NADPH + CO_2 + Formate + UQ + HCO_3^- + MQH_2 + 2 H^+_{in} → 5 $NADP^+$ + 5 ADP + 4 P_i + glyceraldehyde-3-phosphate + UQH_2 + MQ + 2 H^+_{out}	-31.6 ± 15.3 [a]

[a] the free energy was calculated assuming a transmembrane potential of -150 mV

6. Additional pathway analysis

6.1. Pathway analysis for acetyl-CoA production at atmospheric CO2 levels

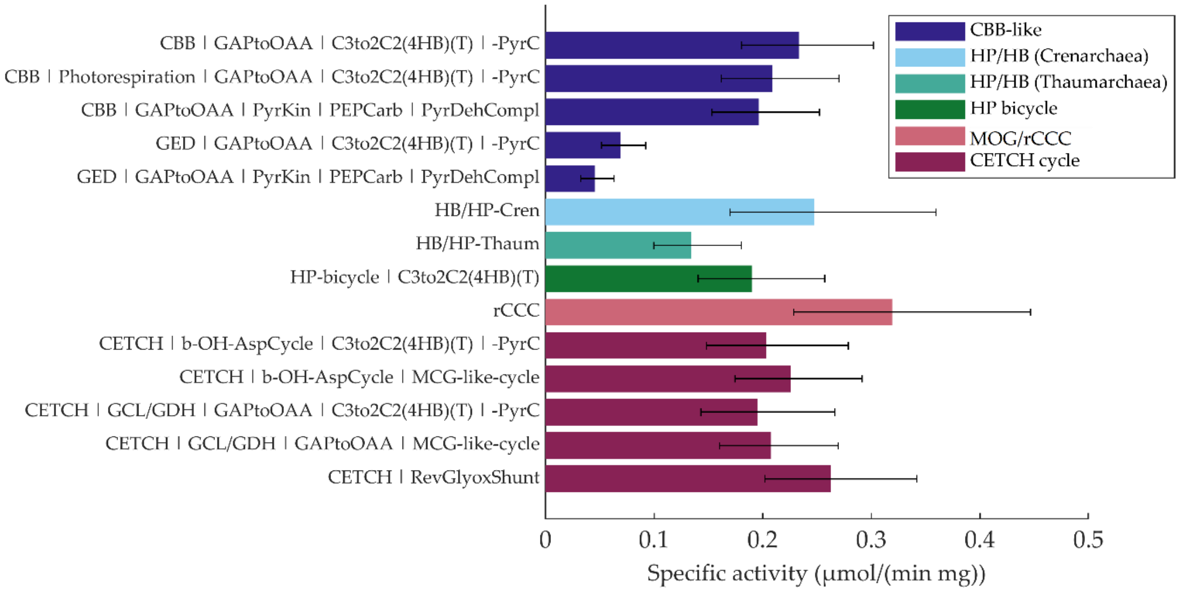

Figure S12: Comparison carbon fixation pathways for the production of **acetyl-CoA** at elevated CO_2 and HCO_3^- concentrations with the Enzyme Cost Minimization algorithm. (w/o SHMT) indicates that the cost of the serine hydroxymethyltransferase are not included in the respective pathway's activity. The concentration of CO_2 was assumed to be 1 mM and the concentration of HCO_3^- to be 10 mM. Pathway specific activities with standard deviations and the full name of the main pathways and the connecting modules to transform their primary product to pyruvate. Pathway abbreviations: CBB(PTS), phosphatase-free CBB cycle; HP-bicycle(Glyox), glyoxylate producing cycle of the 3-HP bicycle; GAPtoPyr, glucolysis; FCL, formaldehyde:NADP+ oxidoreductase; GlyoxToPyr, GCL/GDH route; b-OH-AspCycle, β-hydroxyaspartate cycle; AcCoAtoOAA(HP), acetyl-CoA to oxaloacetate converting module derived from the 3-HP/4-HB cycle using either ADP or AMP producing CoA-ester synthases; rCC-AcCoAtoPyr, acetyl-CoA to pyruvate converting module derived from the rCCC; GlyoxToOAA, glyoxylate to oxaloacetate converting module based on the Serine cycle.

6.2. Pathway analysis using methanol as substrate for pyruvate production with NAD-dependent methanol dehydrogenase

A)

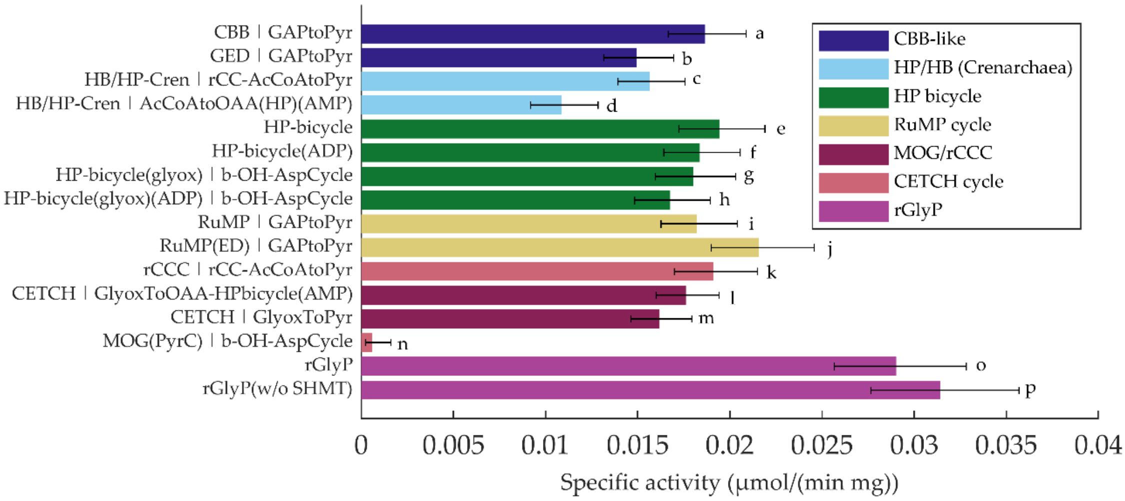

B)

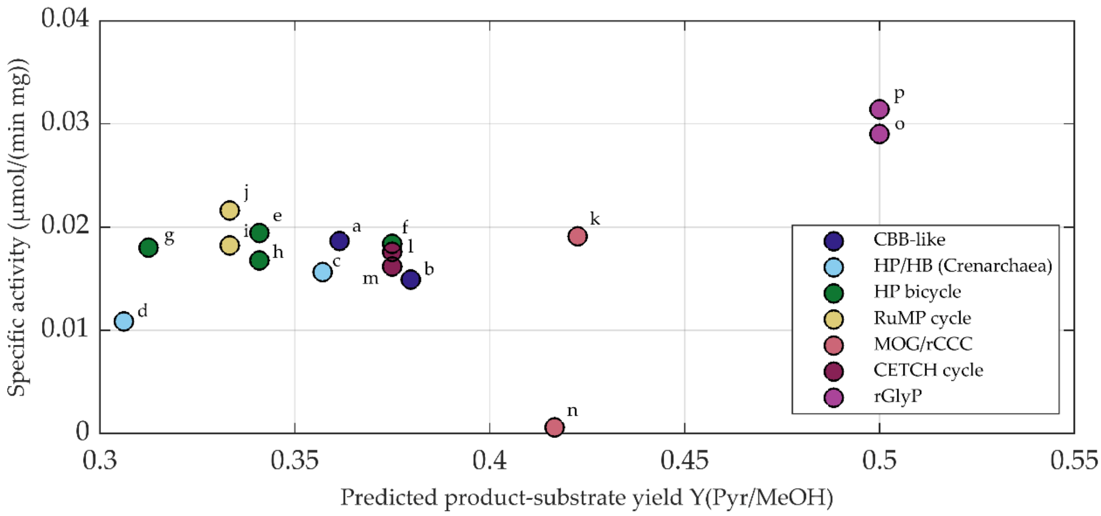

Figure S13: Comparison carbon fixation pathways using **methanol** as a substrate for the production of **pyruvate** with the Enzyme Cost Minimization algorithm. Here, only pathways with the NAD-dependent methanol dehydrogenase are shown. (w/o SHMT) indicates that the cost of the serine hydroxymethyltransferase are not included in the respective pathway's activity. The concentration of CO_2 was assumed to be 1 mM, HCO_3^- to be 10 mM. **A)** Pathway specific activities with standard deviations and the full name of the main pathways and the connecting modules to transform their primary product to pyruvate. Pathway abbreviations: CBB(PTS), phosphatase-free CBB cycle; HP-bicycle(Glyox), glyoxylate producing cycle of the 3-HP bicycle; GAPtoPyr, glucolysis; FCL, formaldehyde:NADP+ oxidoreductase; GlyoxToPyr, GCL/GDH route; b-OH-AspCycle, β-hydroxyaspartate cycle; AcCoAtoOAA(HP), acetyl-CoA to oxaloacetate converting module derived from the 3-HP/4-HB cycle using either ADP or AMP producing CoA-ester synthases; rCC-AcCoAtoPyr, acetyl-CoA to pyruvate converting module derived from the rCCC; GlyoxToOAA, glyoxylate to oxaloacetate converting module based on the Serine cycle. **B)** Pathway specific activities compared to product-substrate yield. The overall stoichiometries of all pathways are listed in supplementary Table S5. The small letters label each pathway and correspond to the labels and the respective pathway combinations in panel A).

6.3. Pathway analysis of biotechnological scenarios for acetyl-CoA production

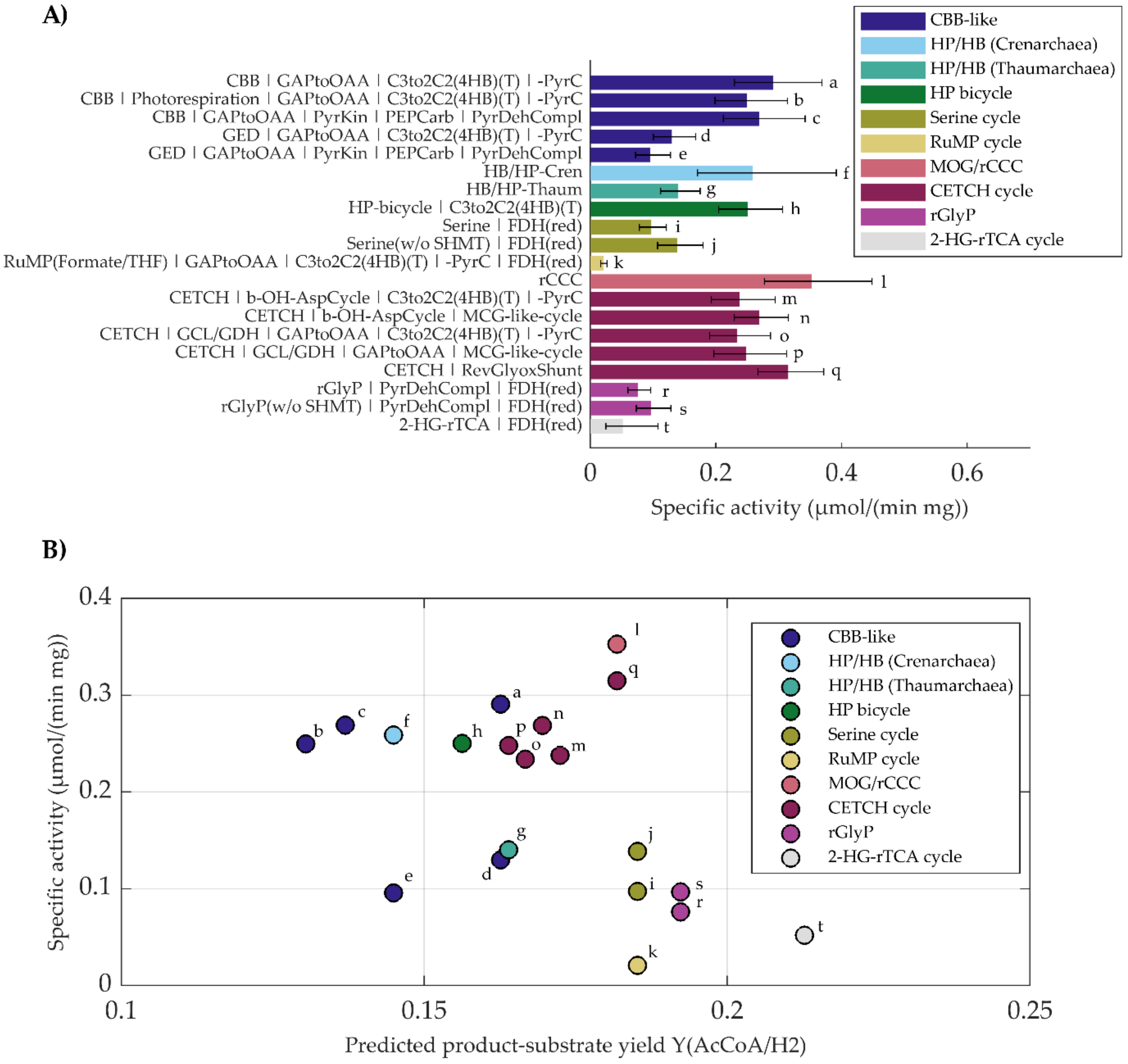

Figure S14: Comparison carbon fixation pathways using **H_2/CO_2** as a substrate for the production of **acetyl-CoA** with the Enzyme Cost Minimization algorithm. (w/o SHMT) indicates that the cost of the serine hydroxymethyltransferase are not included in the respective pathway's activity. The concentration of CO_2 was assumed to be 1 mM, HCO_3^- to be 10 mM. **A)** Pathway specific activities with standard deviations and the full name of the main pathways and the connecting modules to transform their primary product to acetyl-CoA. Pathway abbreviations: CBB(PTS), phosphatase-free CBB cycle; HP-bicycle(Glyox), glyoxylate producing cycle of the 3-HP bicycle; GAPtoPyr, glucolysis; FCL, formaldehyde:NADP+ oxidoreductase; GlyoxToPyr, GCL/GDH route; b-OH-AspCycle, β-hydroxyaspartate cycle; AcCoAtoOAA(HP), acetyl-CoA to oxaloacetate converting module derived from the 3-HP/4-HB cycle using either ADP or AMP producing CoA-ester synthases; rCC-AcCoAtoPyr, acetyl-CoA to pyruvate converting module derived from the rCCC; GlyoxToOAA, glyoxylate to oxaloacetate converting module based on the Serine cycle. **B)** Pathway specific activities compared to product-substrate yield. The overall stoichiometries of all pathways are listed in supplementary Table S5. The small letters label each pathway and correspond to the labels and the respective pathway combinations in panel A).

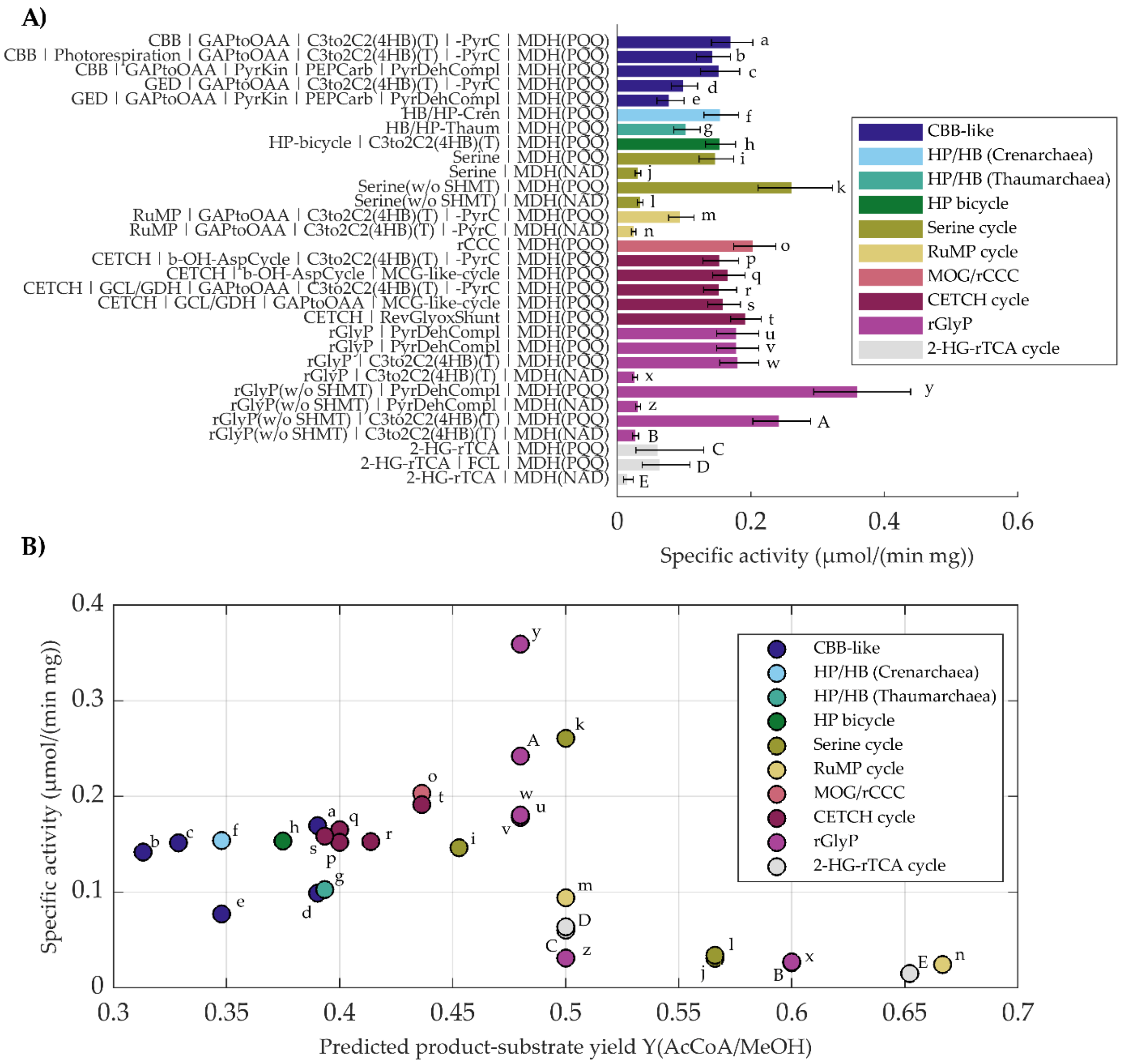

Figure S15: Comparison carbon fixation pathways using **methanol** as a substrate for the production of **acetyl-CoA** with the Enzyme Cost Minimization algorithm. (w/o SHMT) indicates that the cost of the serine hydroxymethyltransferase are not included in the respective pathway's activity. The concentration of CO_2 was assumed to be 1 mM, HCO_3^- to be 10 mM. **A)** Pathway specific activities with standard deviations and the full name of the main pathways and the connecting modules to transform their primary product to acetyl-CoA. Pathway abbreviations: CBB(PTS), phosphatase-free CBB cycle; HP-bicycle(Glyox), glyoxylate producing cycle of the 3-HP bicycle; GAPtoPyr, glucolysis; FCL, formaldehyde:NADP+ oxidoreductase; GlyoxToPyr, GCL/GDH route; b-OH-AspCycle, β-hydroxyaspartate cycle; AcCoAtoOAA(HP), acetyl-CoA to oxaloacetate converting module derived from the 3-HP/4-HB cycle using either ADP or AMP producing CoA-ester synthases; rCC-AcCoAtoPyr, acetyl-CoA to pyruvate converting module derived from the rCCC; GlyoxToOAA, glyoxylate to oxaloacetate converting module based on the Serine cycle. **B)** Pathway specific activities compared to product-substrate yield The overall stoichiometries of all pathways are listed in supplementary Table S5. The small letters label each pathway and correspond to the labels and the respective pathway combinations in panel A).

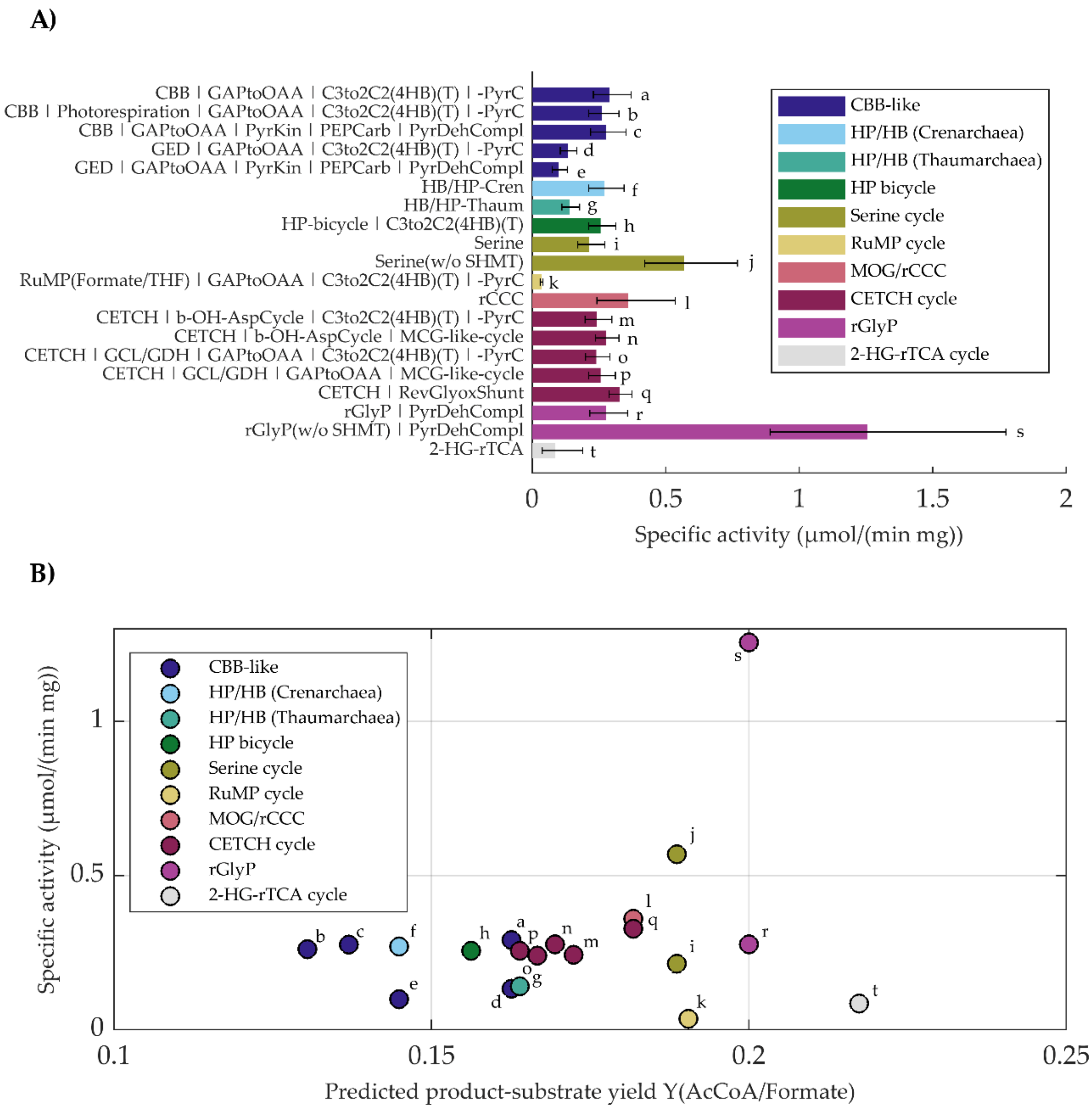

Figure S16: Comparison carbon fixation pathways using **formate** as a substrate for the production of **acetyl-CoA** with the Enzyme Cost Minimization algorithm. (w/o SHMT) indicates that the cost of the serine hydroxymethyltransferase are not included in the respective pathway's activity. The concentration of CO_2 was assumed to be 1 mM, HCO_3^- to be 10 mM. **A)** Pathway specific activities with standard deviations and the full name of the main pathways and the connecting modules to transform their primary product to acetyl-CoA. Pathway abbreviations: CBB(PTS), phosphatase-free CBB cycle; HP-bicycle(Glyox), glyoxylate producing cycle of the 3-HP bicycle; GAPtoPyr, glucolysis; FCL, formaldehyde:NADP+ oxidoreductase; GlyoxToPyr, GCL/GDH route; b-OH-AspCycle, β-hydroxyaspartate cycle; AcCoAtoOAA(HP), acetyl-CoA to oxaloacetate converting module derived from the 3-HP/4-HB cycle using either ADP or AMP producing CoA-ester synthases; rCC-AcCoAtoPyr, acetyl-CoA to pyruvate converting module derived from the rCCC; GlyoxToOAA, glyoxylate to oxaloacetate converting module based on the Serine cycle. **B)** Pathway specific activities compared to product-substrate yield. The overall stoichiometries of all pathways are listed in supplementary Table S5. The small letters label each pathway and correspond to the labels and the respective pathway combinations in panel A).

7. Detailed pathway analysis with single enzyme contributions

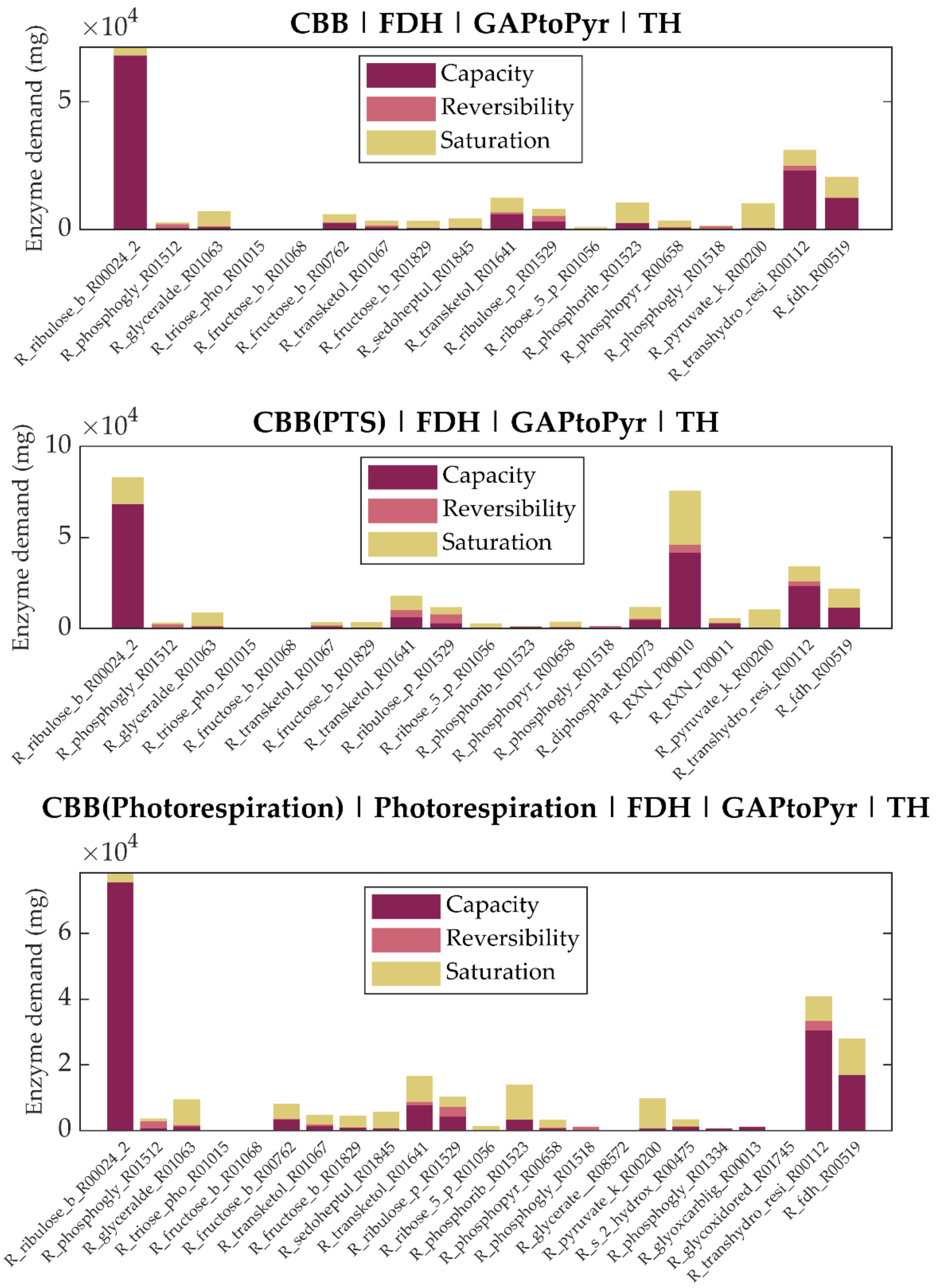

Figure S17: Enzyme demands to sustain a total pathway activity of 1 mmol product per second when using formate as a substrate. Contribution of the capacity, the reversibility and the saturation with substrates or products of each reaction to the demand of enzymes. The figure follows the wording of (Noor et al., 2016). “Capacity”: demand of enzyme caused by a limitation by the catalytic rate constant. “Reversibility”: extra amount of enzyme needed because of a backward flux. “Saturation”: additional enzyme necessary because of undersaturation with a substrate or oversaturation with a product. The values present the optimized state as predicted by the ECM algorithm assuming a CO2 concentration of 1 mM and a HCO_3^- concentration of 10 mM. Pyruvate was chosen as a product for the cycles in this case. Reaction names correspond to their identifiers in the SBtab model file (Supplementary file Reactions_Composite22_model.tsv)

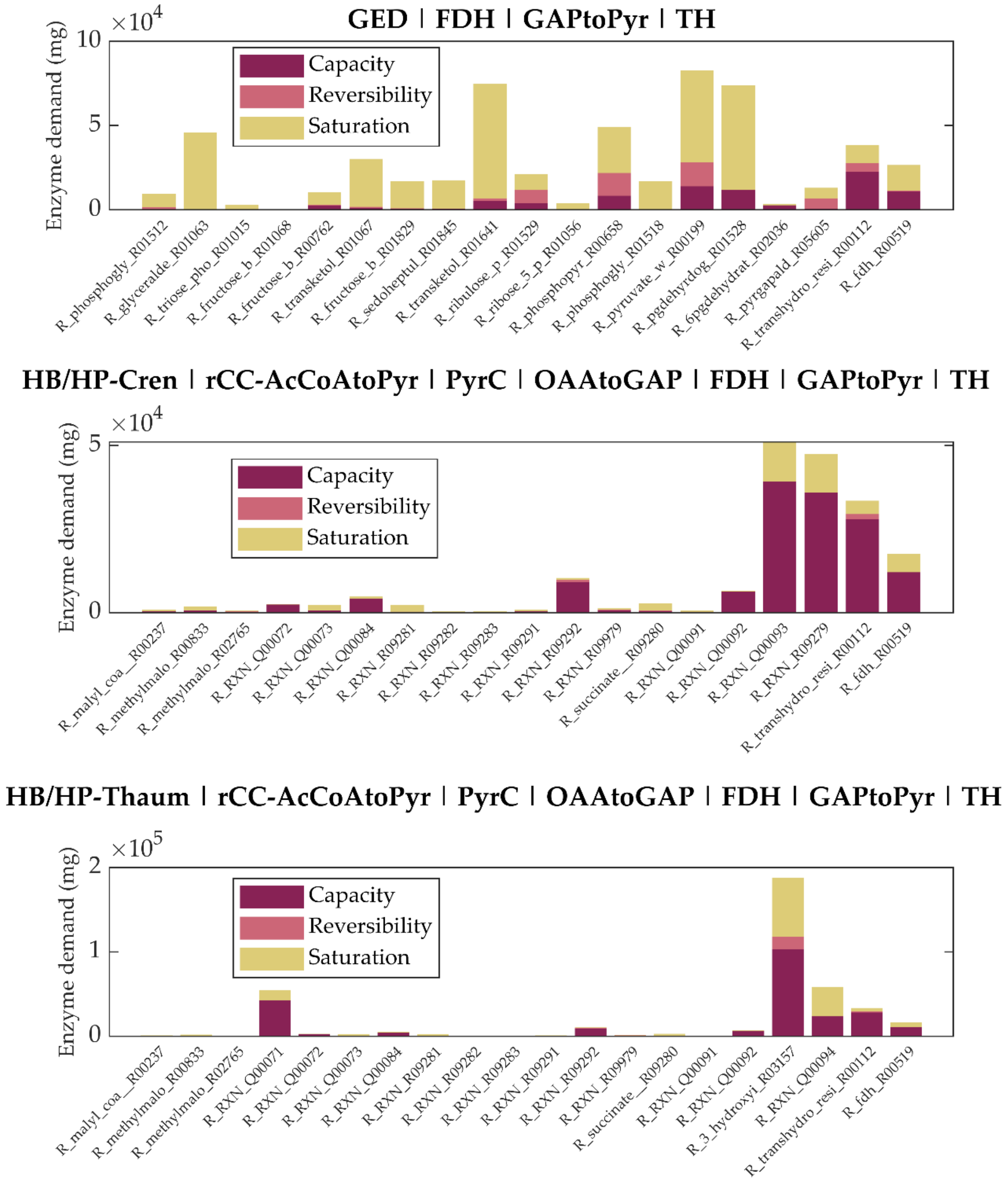

Figure S18: Enzyme demands to sustain a total pathway activity of 1 mmol product per second when using formate as a substrate. Contribution of the capacity, the reversibility and the saturation with substrates or products of each reaction to the demand of enzymes. The figure follows the wording of (Noor et al., 2016). “Capacity”: demand of enzyme caused by a limitation by the catalytic rate constant. “Reversibility”: extra amount of enzyme needed because of a backward flux. “Saturation”: additional enzyme necessary because of undersaturation with a substrate or oversaturation with a product. The values present the optimized state as predicted by the ECM algorithm assuming a CO2 concentration of 1 mM and a HCO_3^- concentration of 10 mM. Pyruvate was chosen as a product for the cycles in this case. Reaction names correspond to their identifiers in the SBtab model file (Supplementary file Reactions_Composite22_model.tsv)

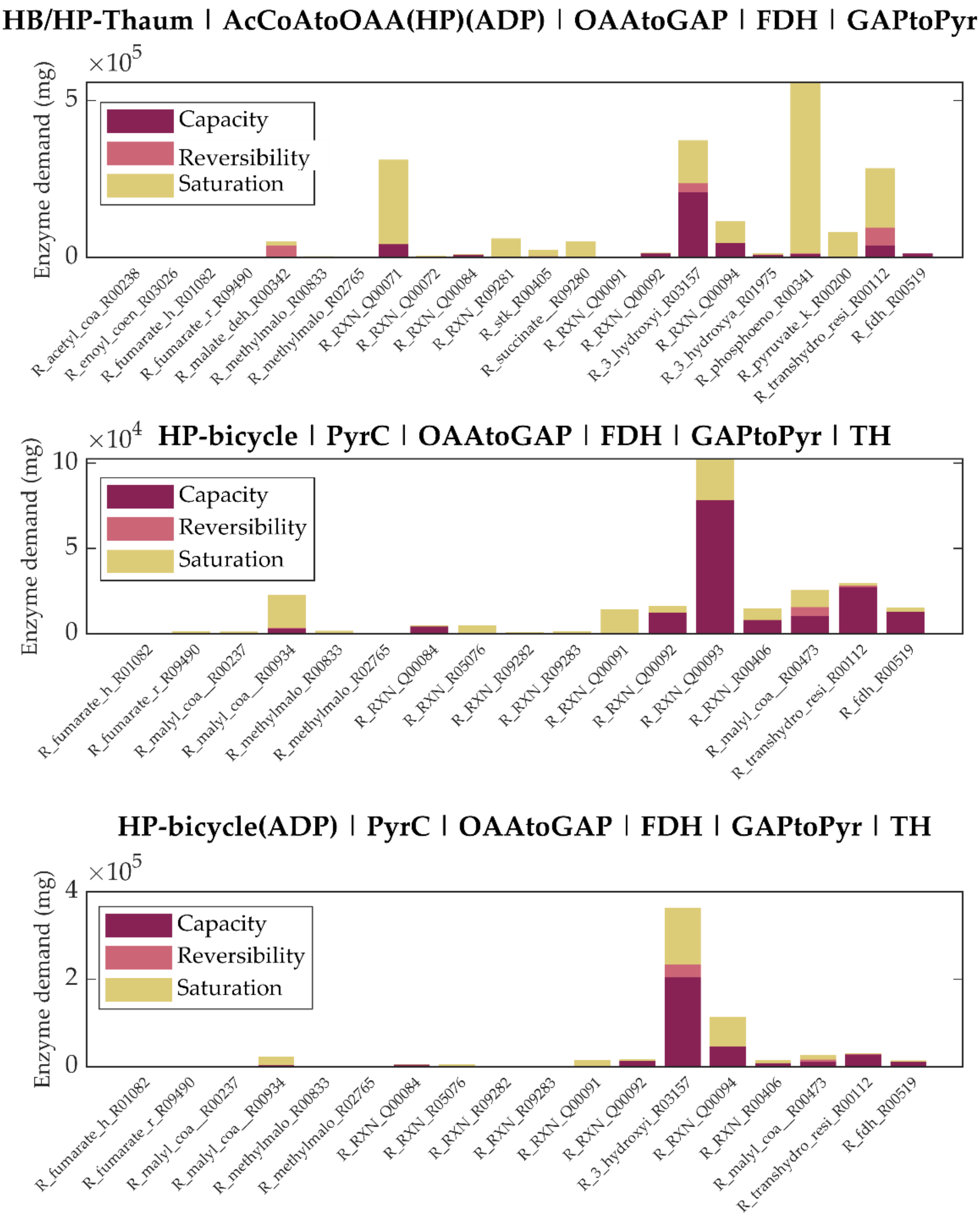

Figure S19: Enzyme demands to sustain a total pathway activity of 1 mmol product per second when using formate as a substrate. Contribution of the capacity, the reversibility and the saturation with substrates or products of each reaction to the demand of enzymes. The figure follows the wording of (Noor et al., 2016). “Capacity”: demand of enzyme caused by a limitation by the catalytic rate constant. “Reversibility”: extra amount of enzyme needed because of a backward flux. “Saturation”: additional enzyme necessary because of undersaturation with a substrate or oversaturation with a product. The values present the optimized state as predicted by the ECM algorithm assuming a CO_2 concentration of 1 mM and a HCO_3^- concentration of 10 mM. Pyruvate was chosen as a product for the cycles in this case. Reaction names correspond to their identifiers in the SBtab model file (Supplementary file Reactions_Composite22_model.tsv)

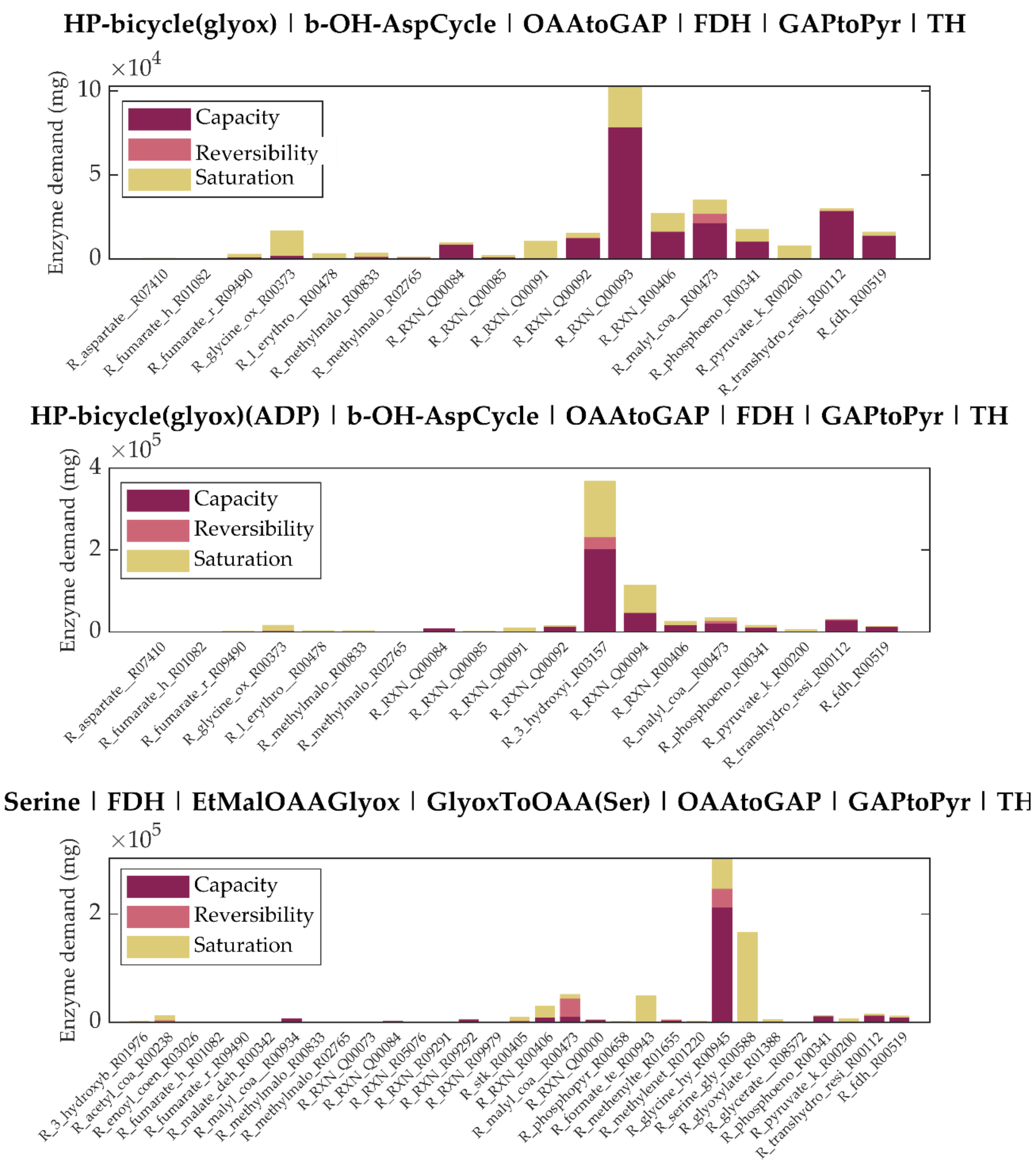

Figure S20: Enzyme demands to sustain a total pathway activity of 1 mmol product per second when using formate as a substrate. Contribution of the capacity, the reversibility and the saturation with substrates or products of each reaction to the demand of enzymes. The figure follows the wording of (Noor et al., 2016). “Capacity”: demand of enzyme caused by a limitation by the catalytic rate constant. “Reversibility”: extra amount of enzyme needed because of a backward flux. “Saturation”: additional enzyme necessary because of undersaturation with a substrate or oversaturation with a product. The values present the optimized state as predicted by the ECM algorithm assuming a CO2 concentration of 1 mM and a HCO_3^- concentration of 10 mM. Pyruvate was chosen as a product for the cycles in this case. Reaction names correspond to their identifiers in the SBtab model file (Supplementary file Reactions_Composite22_model.tsv)

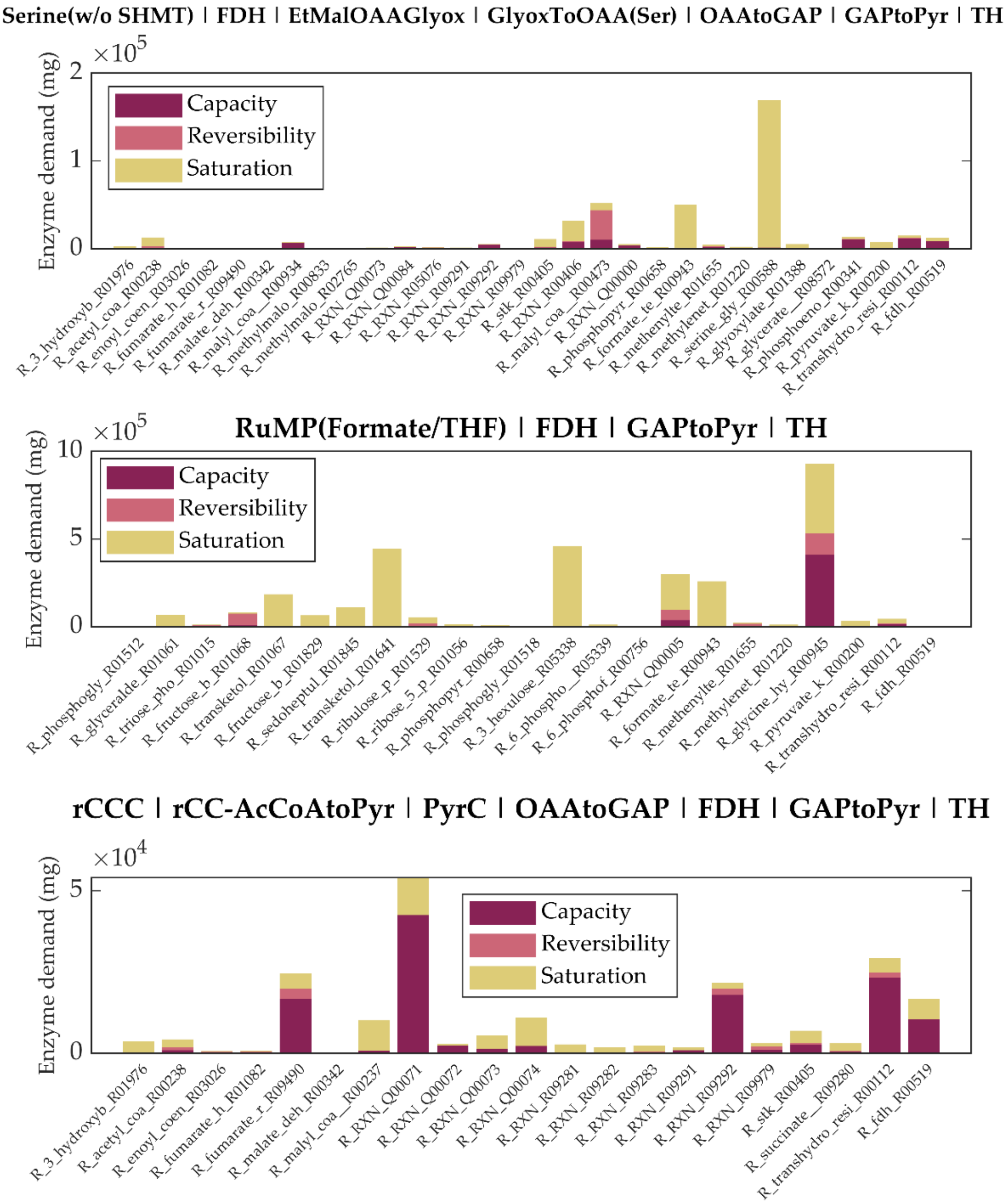

Figure S21: Enzyme demands to sustain a total pathway activity of 1 mmol product per second when using formate as a substrate. Contribution of the capacity, the reversibility and the saturation with substrates or products of each reaction to the demand of enzymes. The figure follows the wording of (Noor et al., 2016). “Capacity”: demand of enzyme caused by a limitation by the catalytic rate constant. “Reversibility”: extra amount of enzyme needed because of a backward flux. “Saturation”: additional enzyme necessary because of undersaturation with a substrate or oversaturation with a product. The values present the optimized state as predicted by the ECM algorithm assuming a CO2 concentration of 1 mM and a HCO_3^- concentration of 10 mM. Pyruvate was chosen as a product for the cycles in this case. Reaction names correspond to their identifiers in the SBtab model file (Supplementary file Reactions_Composite22_model.tsv)

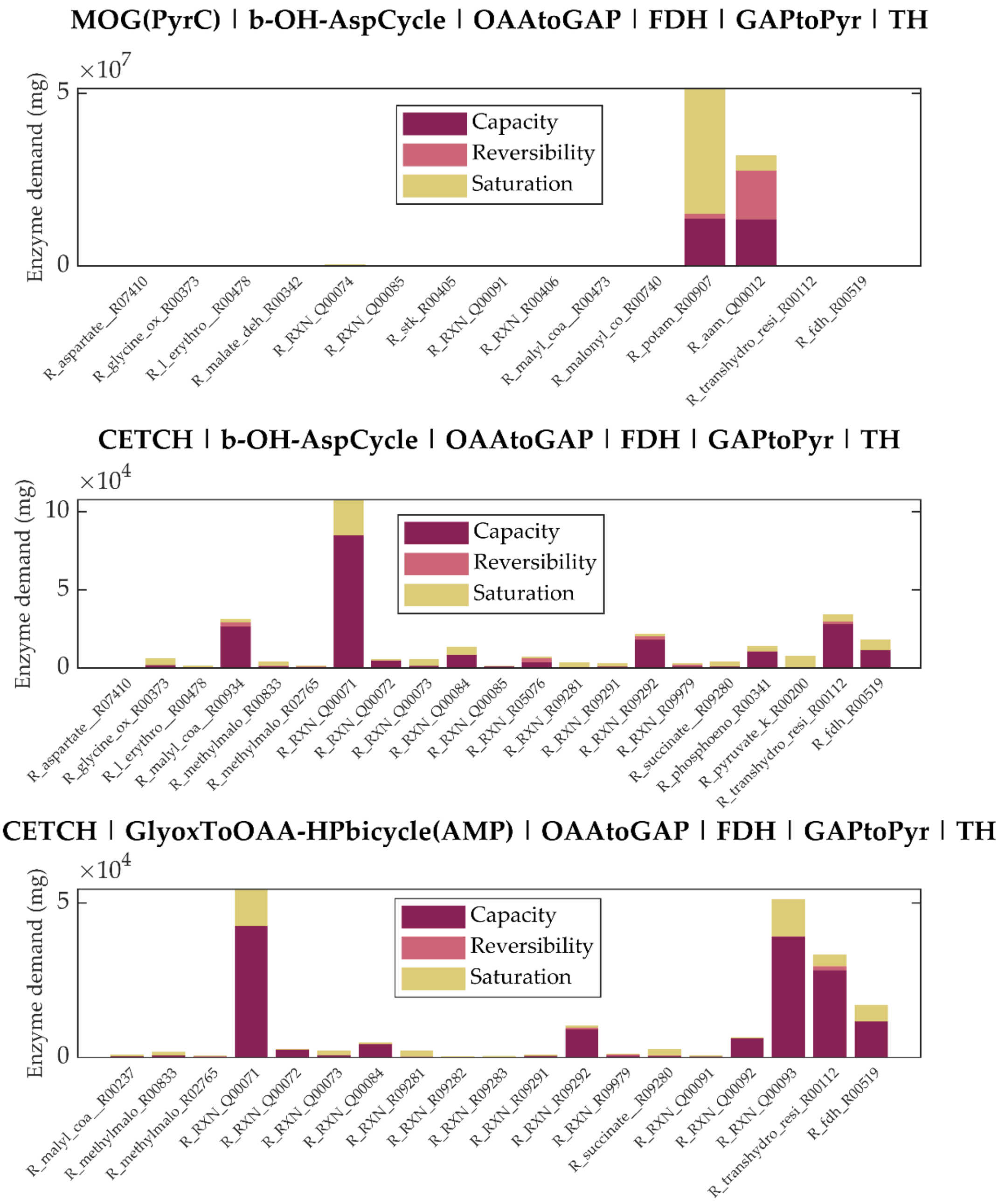

Figure S22: Enzyme demands to sustain a total pathway activity of 1 mmol product per second when using formate as a substrate. Contribution of the capacity, the reversibility and the saturation with substrates or products of each reaction to the demand of enzymes. The figure follows the wording of (Noor et al., 2016). "Capacity": demand of enzyme caused by a limitation by the catalytic rate constant. "Reversibility": extra amount of enzyme needed because of a backward flux. "Saturation": additional enzyme necessary because of undersaturation with a substrate or oversaturation with a product. The values present the optimized state as predicted by the ECM algorithm assuming a CO2 concentration of 1 mM and a HCO_3^- concentration of 10 mM. Pyruvate was chosen as a product for the cycles in this case. Reaction names correspond to their identifiers in the SBtab model file (Supplementary file Reactions_Composite22_model.tsv)

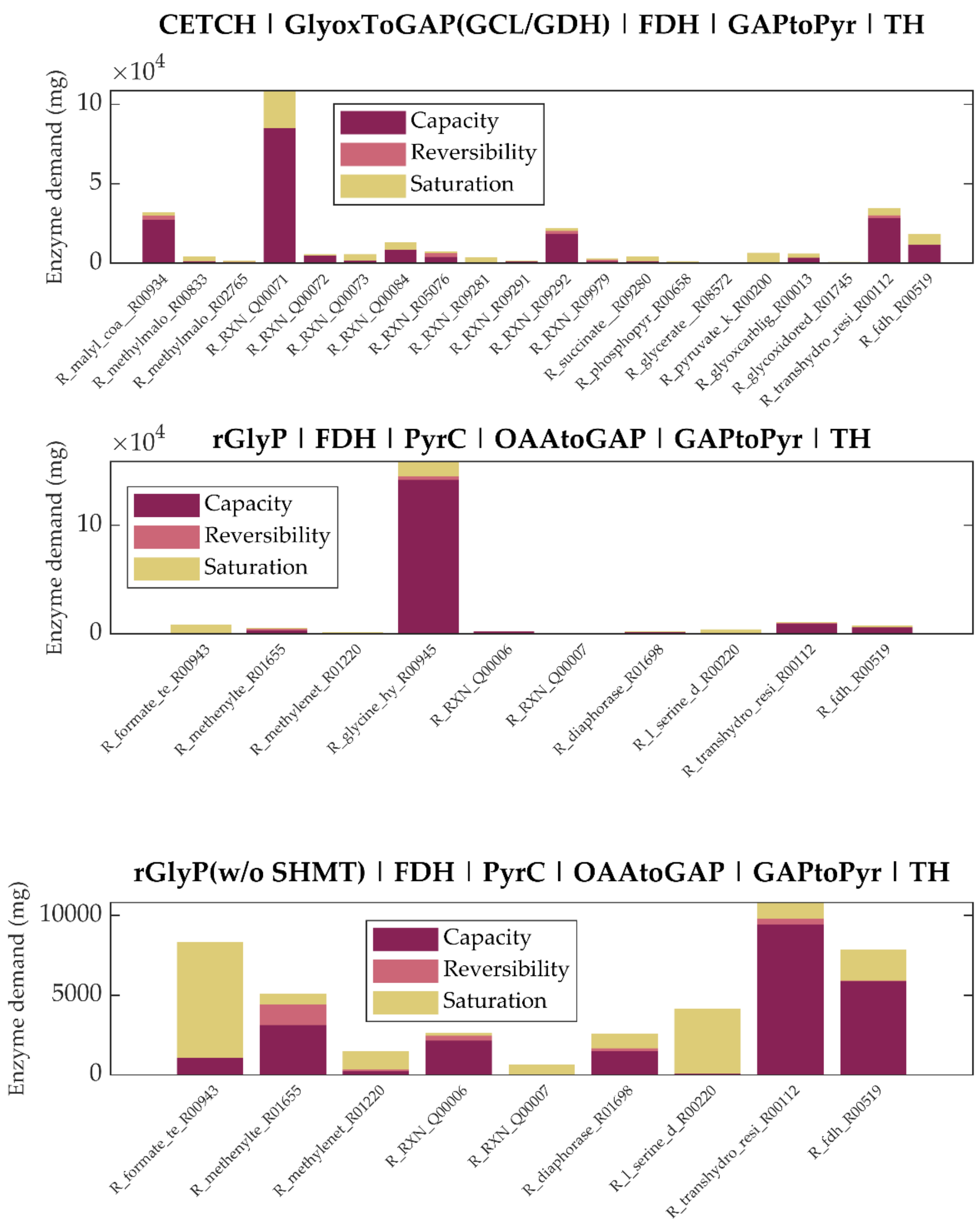

Figure S23: Enzyme demands to sustain a total pathway activity of 1 mmol product per second when using formate as a substrate. Contribution of the capacity, the reversibility and the saturation with substrates or products of each reaction to the demand of enzymes. The figure follows the wording of (Noor et al., 2016). “Capacity”: demand of enzyme caused by a limitation by the catalytic rate constant. “Reversibility”: extra amount of enzyme needed because of a backward flux. “Saturation”: additional enzyme necessary because of undersaturation with a substrate or oversaturation with a product. The values present the optimized state as predicted by the ECM algorithm assuming a CO2 concentration of 1 mM and a HCO_3^- concentration of 10 mM. Pyruvate was chosen as a product for the cycles in this case. Reaction names correspond to their identifiers in the SBtab model file (Supplementary file Reactions_Composite22_model.tsv)

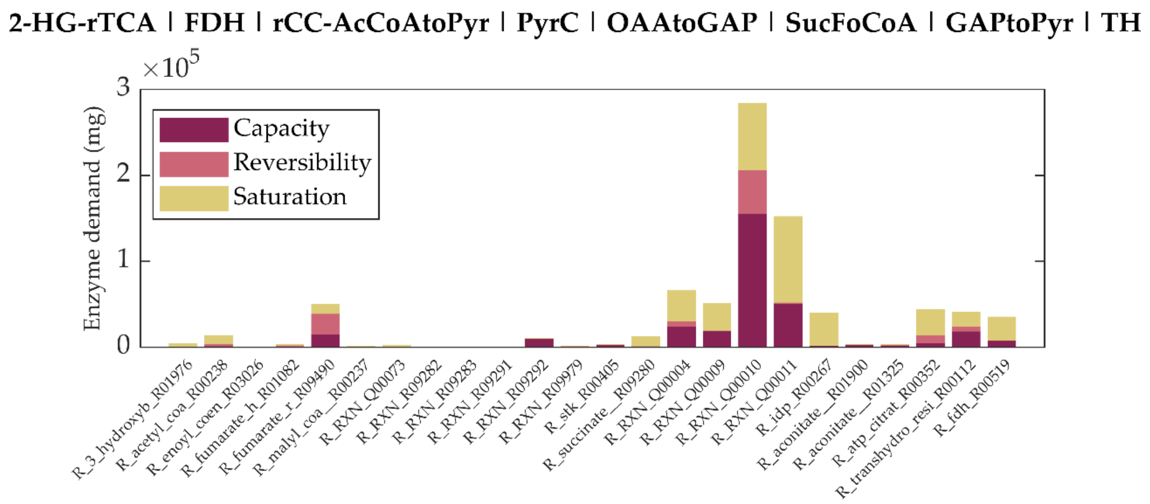

Figure S24: Enzyme demands to sustain a total pathway activity of 1 mmol product per second when using formate as a substrate. Contribution of the capacity, the reversibility and the saturation with substrates or products of each reaction to the demand of enzymes. The figure follows the wording of (Noor et al., 2016). “Capacity”: demand of enzyme caused by a limitation by the catalytic rate constant. “Reversibility”: extra amount of enzyme needed because of a backward flux. “Saturation”: additional enzyme necessary because of undersaturation with a substrate or oversaturation with a product. The values present the optimized state as predicted by the ECM algorithm assuming a CO2 concentration of 1 mM and a HCO_3^- concentration of 10 mM. Pyruvate was chosen as a product for the cycles in this case. Reaction names correspond to their identifiers in the SBtab model file (Supplementary file Reactions_Composite22_model.tsv)

References (Supplement only)

Abbe, K., & Yamada, T. (1982). Purification and properties of pyruvate kinase from Streptococcus mutans. *Journal of Bacteriology*, *149*(1), 299–305. https://doi.org/10.1128/jb.149.1.299-305.1982

Allen, S. H. . (1966). The isolation and characterization of malate-lactate transhydrogenase from *Micrococcus lactilyticus*. *The Journal of Biological Chemistry*, *241(22)*(Nov 25), 5266–5275.

Arfman, N., Bystrykh, L., Govorukhina, N. I., & Dijkhuizen, L. (1990). 3-Hexulose-6-phosphate synthase from thermotolerant methylotroph Bacillus C1. *Methods in Enzymology*, *188*(C), 391–397. https://doi.org/10.1016/0076-6879(90)88062-F

Baetz, A. L., & Allison, M. J. (1990). Purification and characterization of formyl-coenzyme A transferase from Oxalobacter formigenes. *Journal of Bacteriology*, *172*(7), 3537–3540. https://doi.org/10.1128/jb.172.7.3537-3540.1990

Bartsch, O., Hagemann, M., & Bauwe, H. (2008). Only plant-type (GLYK) glycerate kinases produce <scp>d</scp> -glycerate 3-phosphate. *FEBS Letters*, *582*(20), 3025–3028. https://doi.org/10.1016/j.febslet.2008.07.038

Bateman, A. (2019). UniProt: A worldwide hub of protein knowledge. *Nucleic Acids Research*, *47*(D1), D506–D515. https://doi.org/10.1093/nar/gky1049

Baumgart, M., & Bott, M. (2011). Biochemical characterisation of aconitase from Corynebacterium glutamicum. *Journal of Biotechnology*, *154*(2–3), 163–170. https://doi.org/10.1016/j.jbiotec.2010.07.002

Belova, L. L., Sokolov, A. P., Sidorov, I. A., & Trotsenko, Y. A. (1997). Purification and characterization of NADPH-dependent acetoacetyl-CoA reductase from Methylobacterium extorquens. *FEMS Microbiology Letters*, *156*(2), 275–279. https://doi.org/10.1016/S0378-1097(97)00441-2

Berman, K. M., & Cohn, M. (1970). Phosphoenolpyruvate synthetase of *Escherichia coli*. Purification, some properties, and the role of divalent metal ions. *Journal of Biological Chemistry*, *245(20)*(Oct 25), 5309–5318.

Bernhardsgrütter, I., Vögeli, B., Wagner, T., Peter, D. M., Cortina, N. S., Kahnt, J., Bange, G., Engilberge, S., Girard, E., Riobé, F., Maury, O., Shima, S., Zarzycki, J., & Erb, T. J. (2018). The multicatalytic compartment of propionyl-CoA synthase sequesters a toxic metabolite. *Nature Chemical Biology*, *14*(12), 1127–1132. https://doi.org/10.1038/s41589-018-0153-x

Binstock, J. F., & Schulz, H. (1981). Fatty Acid Oxidation Complex from Escherichia coli. *Methods in Enzymology*, *71*(C), 403–411. https://doi.org/10.1016/0076-6879(81)71051-6

Borjian, F., Johnsen, U., Schönheit, P., & Berg, I. A. (2017). Succinyl-CoA: Mesaconate CoA-transferase and mesaconyl-CoA hydratase, enzymes of the methylaspartate cycle in Haloarcula hispanica. *Frontiers in Microbiology*, *8*(SEP). https://doi.org/10.3389/fmicb.2017.01683

Cadet, F., & Meunier, J. C. (1988). pH and kinetic studies of chloroplast sedoheptulose-1,7-bisphosphatase from spinach (Spinacia oleracea). *The Biochemical Journal*, *253*(1), 249–254. https://doi.org/10.1042/bj2530249

Carmo-Silva, A. E., Keys, A. J., Andralojc, P. J., Powers, S. J., Arrabaça, M. C., & Parry, M. A. J. (2010). Rubisco activities, properties, and regulation in three different C4 grasses under drought. *Journal of Experimental Botany*, *61*(9), 2355–2366. https://doi.org/10.1093/jxb/erq071

Chang, K. S., Jeon, H., Seo, S., Lee, Y., & Jin, E. S. (2014). Improvement of the phosphoenolpyruvate carboxylase activity of Phaeodactylum tricornutum PEPCase 1 through protein engineering.

Enzyme and Microbial Technology, *60*, 64–71. https://doi.org/10.1016/j.enzmictec.2014.04.007

Chen, D., Ruzicka, F. J., & Frey, P. A. (2000). A novel lysine 2,3-aminomutase encoded by the yodO gene of Bacillus subtilis: Characterization and the observation of organic radical intermediates. *Biochemical Journal*, *348*(3), 539–549. https://doi.org/10.1042/0264-6021:3480539

Chen, Z. H., Walker, R. P., Acheson, R. M., & Leegood, R. C. (2002). Phosphoenolpyruvate carboxykinase assayed at physiological concentrations of metal ions has a high affinity for CO2. *Plant Physiology*, *128*(1), 160–164. https://doi.org/10.1104/pp.010431

Chistoserdova, L. V., & Lidstrom, M. E. (1991). Purification and characterization of hydroxypyruvate reductase from the facultative methylotroph Methylobacterium extorquens AM1. *Journal of Bacteriology*, *173*(22), 7228–7232. https://doi.org/10.1128/jb.173.22.7228-7232.1991

Chou, A., Clomburg, J. M., Qian, S., & Gonzalez, R. (2019). 2-Hydroxyacyl-CoA lyase catalyzes acyloin condensation for one-carbon bioconversion. *Nature Chemical Biology*, *15*(9), 900–906. https://doi.org/10.1038/s41589-019-0328-0

Dayem, L. C., Carney, J. R., Santi, D. V., Pfeifer, B. A., Khosla, C., & Kealey, J. T. (2002). Metabolic engineering of a methylmalonyl-CoA mutase-epimerase pathway for complex polyketide biosynthesis in Escherichia coli. *Biochemistry*, *41*(16), 5193–5201. https://doi.org/10.1021/bi015593k

Dev, I. K., & Harvey, R. J. (1978). A complex of N5,N10-methylentetrahydrofolate dehydrogenase and N5,N10-methenyltetrahydrofolate cyclohydrolase in Escherichia coli. Purification subunit structure, and allosteric inhibition by N10-formyltetrahydrofolate. *Journal of Biological Chemistry*, *253*(12), 4245–4253. https://doi.org/10.1016/S0021-9258(17)34711-7

Erales, J., Gontero, B., & Maberly, S. C. (2008). SPECIFICITY AND FUNCTION OF GLYCERALDEHYDE-3-PHOSPHATE DEHYDROGENASE IN A FRESHWATER DIATOM, *ASTERIONELLA FORMOSA* (BACILLARIOPHYCEAE) [1]. *Journal of Phycology*, *44*(6), 1455–1464. https://doi.org/10.1111/j.1529-8817.2008.00600.x

Erb, T. J., Frerichs-Revermann, L., Fuchs, G., & Alber, B. E. (2010). The apparent malate synthase activity of Rhodobacter sphaeroides is due to two paralogous enzymes, (3S)-malyl-coenzyme A (CoA)/β-methylmalyl-CoA lyase and (3S)-malyl-CoA thioesterase. *Journal of Bacteriology*, *192*(5), 1249–1258. https://doi.org/10.1128/JB.01267-09

Erb, T. J., Rétey, J., Fuchs, G., & Alber, B. E. (2008). Ethylmalonyl-CoA mutase from Rhodobacter sphaeroides defines a new subclade of coenzyme B12-dependent Acyl-CoA mutases. *Journal of Biological Chemistry*, *283*(47), 32283–32293. https://doi.org/10.1074/jbc.M805527200

Featherston, E. R., Rose, H. R., McBride, M. J., Taylor, E. M., Boal, A. K., & Cotruvo, J. A. (2019). Biochemical and Structural Characterization of XoxG and XoxJ and Their Roles in Lanthanide-Dependent Methanol Dehydrogenase Activity. *ChemBioChem*, *20*(18), 2360–2372. https://doi.org/10.1002/cbic.201900184

Ferenci, T., Strom, T., & Quayle, J. R. (1974). Purification and properties of 3 hexulose phosphate synthase and phospho 3 hexuloisomerase from Methylococcus capsulatus. *Biochemical Journal*, *144*(3), 477–486. https://doi.org/10.1042/bj1440477

Flamholz, A., Noor, E., Bar-Even, A., & Milo, R. (2012). eQuilibrator - The biochemical thermodynamics calculator. *Nucleic Acids Research*, *40*(D1), D770–D775. https://doi.org/10.1093/nar/gkr874

Friedmann, S., Steindorf, A., Alber, B. E., & Fuchs, G. (2006). Properties of succinyl-coenzyme A:L-malate coenzyme A transferase and its role in the autotrophic 3-hydroxypropionate cycle of Chloroflexus aurantiacus. *Journal of Bacteriology*, *188*(7), 2646–2655.

https://doi.org/10.1128/JB.188.7.2646-2655.2006

Fujiwara, K., & Motokawa, Y. (1983). Mechanism of the glycine cleavage reaction. Steady state kinetic studies of the P-protein-catalyzed reaction. *The Journal of Biological Chemistry*, *258(13)*(July 10), 8156–8162.

Gannon, F., Bridgeland, E. S., & Jones, K. M. (1977). L Serine dehydratase from Arthrobacter globiformis. *Biochemical Journal*, *161*(2), 345–355. https://doi.org/10.1042/bj1610345

Gotto, A. M., & Kornberg, H. L. (1961). The metabolism of C2 compounds in micro-organisms. 7. Preparation and properties of crystalline tartronic semialdehyde reductase. *The Biochemical Journal*, *81*, 273–284. https://doi.org/10.1042/bj0810273

GRAÑA, X., UREÑA, J., LUDEVID, D., CARRERAS, J., & CLIMENT, F. (1989). Purification, characterization and immunological properties of 2,3-bisphosphoglycerate-independent phosphoglycerate mutase from maize (Zea mays) seeds. *European Journal of Biochemistry*, *186*(1–2), 149–153. https://doi.org/10.1111/j.1432-1033.1989.tb15189.x

Gurr, J. A., & Jones, K. M. (1977). Purification and characterization of pyruvate carboxylase from Arthrobacter globiformis. *Archives of Biochemistry and Biophysics*, *179*(2), 444–455. https://doi.org/10.1016/0003-9861(77)90132-1

Hammerstedt, R. H., Möhler, H., Decker, K. A., Ersfeld, D., & Wood, W. A. (1975). 2-Keto-3-deoxy-6-phosphogluconic Aldolase from Pseudomonas putida. *Methods in Enzymology*, *42*(C), 258–264. https://doi.org/10.1016/0076-6879(75)42123-1

Häusler, R. E., Holtum, J. A. M., & Latzko, E. (1987). CO2 is the inorganic carbon substrate of NADP malic enzymes from *Zea mays* and from wheat germ. *European Journal of Biochemistry*, *163*(3), 619–626. https://doi.org/10.1111/j.1432-1033.1987.tb10911.x

Hawkins, A. B., Adams, M. W. W., & Kelly, R. M. (2014). Conversion of 4-hydroxybutyrate to acetyl coenzyme A and its anapleurosis in the Metallosphaera sedula 3-hydroxypropionate/4-hydroxybutyrate carbon fixation pathway. *Applied and Environmental Microbiology*, *80*(8), 2536–2545. https://doi.org/10.1128/AEM.04146-13

Hayaishi, O., Nishizuka, Y., Tatibana, M., Takeshita, M., & Kuno, S. (1961). Enzymatic studies on the metabolism of beta-alanine. *The Journal of Biological Chemistry*, *236*(3), 781–790. https://doi.org/10.1016/s0021-9258(18)64309-1

Hederstedt, L., & Heden, L. O. (1989). New properties of Bacillus subtilis succinate dehydrogenase altered at the active site. The apparent active site thiol of succinate oxidoreductases is dispensable for succinate oxidation. *Biochemical Journal*, *260*(2), 491–497. https://doi.org/10.1042/bj2600491

Hedl, M., Sutherlin, A., Imogen Wilding, E., Mazzulla, M., McDevitt, D., Lane, P., Burgner, J. W., Lehnbeuter, K. R., Stauffacher, C. V., Gwynn, M. N., & Rodwell, V. W. (2002). Enterococcus faecalis acetoacetyl-coenzyme A thiolase/3-hydroxy-3-methylglutaryl-coenzyme A reductase, a dual-function protein of isopentenyl diphosphate biosynthesis. *Journal of Bacteriology*, *184*(8), 2116–2122. https://doi.org/10.1128/JB.184.8.2116-2122.2002

Houston, B., & Nimmo, H. G. (1984). Purification and some kinetic properties of rat liver ATP citrate lyase. *Biochemical Journal*, *224*(2), 437–443. https://doi.org/10.1042/bj2240437

Hu, X., Zhang, J. W., Persson, A., & Rydström, J. (1995). Characterization of the interaction of NADH with proton pumping E. coli transhydrogenase reconstituted in the absence and in the presence of bacteriorhodopsin. *BBA - Bioenergetics*, *1229*(1), 64–72. https://doi.org/10.1016/0005-2728(94)00187-A

Hügler, M., Menendez, C., Schägger, H., & Fuchs, G. (2002). Malonyl-coenzyme a reductase from Chloroflexus aurantiacus, a key enzyme of the 3-hydroxypropionate cycle for autotrophic CO2 fixation. *Journal of Bacteriology*, *184*(9), 2404–2410. https://doi.org/10.1128/JB.184.9.2404-2410.2002

Husic, H. D., & Tolbert, N. E. (1984). Anion and divalent cation activation of phosphoglycolate phosphatase from leaves. *Archives of Biochemistry and Biophysics*, *229*(1), 64–72. https://doi.org/10.1016/0003-9861(84)90130-9

IZUMI, Y., YOSHIDA, T., & YAMADA, H. (1990). Purification and characterization of serine-glyoxylate aminotransferase from a serine-producing methylotroph, Hyphomicrobium methylovorum GM2. *European Journal of Biochemistry*, *190*(2), 285–290. https://doi.org/10.1111/j.1432-1033.1990.tb15574.x

Jeske, L., Placzek, S., Schomburg, I., Chang, A., & Schomburg, D. (2019). BRENDA in 2019: A European ELIXIR core data resource. *Nucleic Acids Research*, *47*(D1), D542–D549. https://doi.org/10.1093/nar/gky1048

Jortzik, E., Mailu, B. M., Preuss, J., Fischer, M., Bode, L., Rahlfs, S., & Becker, K. (2011). Glucose-6-phosphate dehydrogenase-6-phosphogluconolactonase: A unique bifunctional enzyme from Plasmodium falciparum. *Biochemical Journal*, *436*(3), 641–650. https://doi.org/10.1042/BJ20110170

Kanao, T., Kawamura, M., Fukui, T., Atomi, H., & Imanaka, T. (2002). Characterization of isocitrate dehydrogenase from the green sulfur bacterium *Chlorobium limicola*. *European Journal of Biochemistry*, *269*(7), 1926–1931. https://doi.org/10.1046/j.1432-1033.2002.02849.x

Kanehisa, M., & Goto, S. (2000). KEGG: Kyoto Encyclopedia of Genes and Genomes. In *Nucleic Acids Research* (Vol. 28, Issue 1, pp. 27–30). Oxford University Press. https://doi.org/10.1093/nar/28.1.27

Kaplun, A., Binshtein, E., Vyazmensky, M., Steinmetz, A., Barak, Z., Chipman, D. M., Tittmann, K., & Shaanan, B. (2008). Glyoxylate carboligase lacks the canonical active site glutamate of thiamine-dependent enzymes. *Nature Chemical Biology*, *4*(2), 113–118. https://doi.org/10.1038/nchembio.62

Karsten, W. E., Cook, P. F., Ohshiro, T., & Izumi, Y. (2001). Initial velocity, spectral, and pH studies of the serine-glyoxylate aminotransferase from Hyphomicrobiuim methylovorum. *Archives of Biochemistry and Biophysics*, *388*(2), 267–275. https://doi.org/10.1006/abbi.2001.2294

Kelley-Loughnane, N., Biolsi, S. A., Gibson, K. M., Lu, G., Hehir, M. J., Phelan, P., & Kantrowitz, E. R. (2002). Purification, kinetic studies, and homology model of Escherichia coli fructose-1,6-bisphosphatase. *Biochimica et Biophysica Acta - Protein Structure and Molecular Enzymology*, *1594*(1), 6–16. https://doi.org/10.1016/S0167-4838(01)00261-8

Kimura, Y., Kojyo, T., Kimura, I., & Sato, M. (1998). Propionyl-coA carboxylase of Myxococcus xanthus: Catalytic properties and function in developing cells. *Archives of Microbiology*, *170*(3), 179–184. https://doi.org/10.1007/s002030050631

Könneke, M., Schubert, D. M., Brown, P. C., Hügler, M., Standfest, S., Schwander, T., Schada Von Borzyskowski, L., Erb, T. J., Stahl, D. A., & Berg, I. A. (2014). Ammonia-oxidizing archaea use the most energy-efficient aerobic pathway for CO2 fixation. *Proceedings of the National Academy of Sciences of the United States of America*, *111*(22), 8239–8244. https://doi.org/10.1073/pnas.1402028111

Kotlarz, D., & Buc, H. (1982). Phosphofructokinases from Escherichia coli. *Methods in Enzymology*, *90*(C), 60–70. https://doi.org/10.1016/S0076-6879(82)90107-0

Le, S. B., Heggeset, T. M. B., Haugen, T., Nærdal, I., & Brautaset, T. (2017). 6-Phosphofructokinase and ribulose-5-phosphate 3-epimerase in methylotrophic Bacillus methanolicus ribulose monophosphate cycle. *Applied Microbiology and Biotechnology*, *101*(10), 4185–4200. https://doi.org/10.1007/s00253-017-8173-0

Liebermeister, W., Uhlendorf, J., & Klipp, E. (2010). Modular rate laws for enzymatic reactions: Thermodynamics, elasticities and implementation. *Bioinformatics*, *26*(12), 1528–1534. https://doi.org/10.1093/bioinformatics/btq141

Lin, H. Y., Kao, Y. H., Chen, S. T., & Meng, M. (2009). Effects of inherited mutations on catalytic activity and structural stability of human glucose-6-phosphate isomerase expressed in Escherichia coli. *Biochimica et Biophysica Acta - Proteins and Proteomics*, *1794*(2), 315–323. https://doi.org/10.1016/j.bbapap.2008.11.004

MacKintosh, C., & Nimmo, H. G. (1988). Purification and regulatory properties of isocitrate lyase from Escherichia coli ML308. *The Biochemical Journal*, *250*(1), 25–31. https://doi.org/10.1042/bj2500025

Maklashina, E., & Cecchini, G. (1999). Comparison of catalytic activity and inhibitors of quinone reactions of succinate dehydrogenase (succinate-ubiquinone oxidoreductase) and fumarate reductase (menaquinol-fumarate oxidoreductase) from Escherichia coli. *Archives of Biochemistry and Biophysics*, *369*(2), 223–232. https://doi.org/10.1006/abbi.1999.1359

Marx, C. J., Laukel, M., Vorholt, J. A., & Lidstrom, M. E. (2003). Purification of the Formate-Tetrahydrofolate Ligase from Methylobacterium extorquens AM1 and Demonstration of Its Requirement for Methylotrophic Growth. *Journal of Bacteriology*, *185*(24), 7169–7175. https://doi.org/10.1128/JB.185.24.7169-7175.2003

Mathur, D., Malik, G., & Garg, L. C. (2006). Biochemical and functional characterization of triosephosphate isomerase from Mycobacterium tuberculosis H37Rv. *FEMS Microbiology Letters*, *263*(2), 229–235. https://doi.org/10.1111/j.1574-6968.2006.00420.x

Meister, M., Saum, S., Alber, B. E., & Fuchs, G. (2005). L-malyl-coenzyme A/β-methylmalyl-coenzyme A lyase is involved in acetate assimilation of the isocitrate lyase-negative bacterium Rhodobacter capsulatus. *Journal of Bacteriology*, *187*(4), 1415–1425. https://doi.org/10.1128/JB.187.4.1415-1425.2005

Meyer, M., Schweiger, P., & Deppenmeier, U. (2015). Succinic semialdehyde reductase Gox1801 from Gluconobacter oxydans in comparison to other succinic semialdehyde-reducing enzymes. *Applied Microbiology and Biotechnology*, *99*(9), 3929–3939. https://doi.org/10.1007/s00253-014-6191-8

MIYAZAKI, S. S., TOKI, S., IZUMI, Y., & YAMADA, H. (1987). Purification and characterization of a serine hydroxymethyltransferase from an obligate methylotroph, Hyphomicrobium methylovorum GM2. *European Journal of Biochemistry*, *162*(3), 533–540. https://doi.org/10.1111/j.1432-1033.1987.tb10672.x

Moskowitz, G. J., & Merrick, J. M. (1969). Metabolism of Poly-β-hydroxybutyrate. II. Enzymatic Synthesis of D-(-)-β-Hydroxybutyryl Coenzyme A by an Enoyl Hydrase from Rhodospirillum rubrum. *Biochemistry*, *8*(7), 2748–2755. https://doi.org/10.1021/bi00835a009

Muslin, E. H., Li, D., Stevens, F. J., Donnelly, M., Schiffer, M., & Anderson, L. E. (1995). Engineering a domain-locking disulfide into a bacterial malate dehydrogenase produces a redox-sensitive enzyme. *Biophysical Journal*, *68*(6), 2218–2223. https://doi.org/10.1016/S0006-3495(95)80430-3

Nakahara, K., Yamamoto, H., Miyake, C., & Yokota, A. (2003). Purification and characterization of

class-I and class-II fructose-1,6-bisphosphate aldolases from the cyanobacterium Synechocystis sp. PCC6803. *Plant and Cell Physiology*, *44*(3), 326–333. https://doi.org/10.1093/pcp/pcg044

Nakano, T., Ashida, H., Mizohata, E., Matsumura, H., & Yokota, A. (2010). An evolutionally conserved Lys122 is essential for function in Rhodospirillum rubrum bona fide RuBisCO and Bacillus subtilis RuBisCO-like protein. *Biochemical and Biophysical Research Communications*, *392*(2), 212–216. https://doi.org/10.1016/j.bbrc.2010.01.017

NARINDRASORASAK, S., & BRIDGER, W. A. (1977). Phosphoenolpyruvate Synthetase of *Escherichia coli*: molecular weight, subunit composition, and identification of phosphohistidine in phosphoenzyme intermediate. *Journal of Biological Chemistry*, *252(10)*(May 25), 3121–3127.

Neuburger, M., Polidori, A. M., Piètre, E., Faure, M., Jourdain, A., Bourguignon, J., Pucci, B., & Douce, R. (2000). Interaction between the lipoamide-containing H-protein and the lipoamide dehydrogenase (L-protein) of the glycine decarboxylase multienzyme system. *European Journal of Biochemistry*, *267*(10), 2882–2889. https://doi.org/10.1046/j.1432-1327.2000.01301.x

Nolte, J. C., Schürmann, M., Schepers, C. L., Vogel, E., Wübbeler, J. H., & Steinbüchel, A. (2014). Novel characteristics of succinate coenzyme a (succinate-coa) ligases: Conversion of malate to malyl-coa and coa-thioester formation of succinate analogues in vitro. *Applied and Environmental Microbiology*, *80*(1), 166–176. https://doi.org/10.1128/AEM.03075-13

Noor, E., Flamholz, A., Bar-Even, A., Davidi, D., Milo, R., & Liebermeister, W. (2016). The Protein Cost of Metabolic Fluxes: Prediction from Enzymatic Rate Laws and Cost Minimization. *PLoS Computational Biology*, *12*(11), e1005167. https://doi.org/10.1371/journal.pcbi.1005167

Okamura-Ikeda, K., Kameoka, N., Fujiwara, K., & Motokawa, Y. (2003). Probing the H-protein-induced conformational change and the function of the N-terminal region of Escherichia coli T-protein of the glycine cleavage system by limited proteolysis. *Journal of Biological Chemistry*, *278*(12), 10067–10072. https://doi.org/10.1074/jbc.M210853200

Padovani, D., & Banerjee, R. (2006). Assembly and protection of the radical enzyme, methylmalonyl-CoA mutase, by its chaperone. *Biochemistry*, *45*(30), 9300–9306. https://doi.org/10.1021/bi0604532

Pennati, A., & Gadda, G. (2009). Involvement of ionizable groups in catalysis of human liver glycolate oxidase. *Journal of Biological Chemistry*, *284*(45), 31214–31222. https://doi.org/10.1074/jbc.M109.040063

Pomper, B. K., Vorholt, J. A., Chistoserdova, L., Lidstrom, M. E., & Thauer, R. K. (1999). A methenyl tetrahydromethanopterin cyclohydrolase and a methenyl tetrahydrofolate cyclohydrolase in Methylobacterium extorquens AM1. *European Journal of Biochemistry*, *261*(2), 475–480. https://doi.org/10.1046/j.1432-1327.1999.00291.x

Price, L. J., Herbert, D., Moss, S. R., Cole, D. J., & Harwood, J. L. (2003). Graminicide insensitivity correlates with herbicide-binding co-operativity on acetyl-CoA carboxylase isoforms. *Biochemical Journal*, *375*(2), 415–423. https://doi.org/10.1042/BJ20030665

Ranganathan, N. S., Srere, P. A., & Linn, T. C. (1980). Comparison of phospho- and dephospho-ATP citrate lyase. *Archives of Biochemistry and Biophysics*, *204*(1), 52–58. https://doi.org/10.1016/0003-9861(80)90006-5

Reshetnikov, A. S., Rozova, O. N., Khmelenina, V. N., Mustakhimov, I. I., Beschastny, A. P., Murrell, J. C., & Trotsenko, Y. A. (2008). Characterization of the pyrophosphate-dependent 6-phosphofructokinase from Methylococcus capsulatus Bath. *FEMS Microbiology Letters*, *288*(2), 202–210. https://doi.org/10.1111/j.1574-6968.2008.01366.x

Sasikaran, J., Ziemski, M., Zadora, P. K., Fleig, A., & Berg, I. A. (2014). Bacterial itaconate degradation

promotes pathogenicity. *Nature Chemical Biology*, *10*(5), 371–377. https://doi.org/10.1038/nchembio.1482

Satanowski, A., Dronsella, B., Noor, E., Vögeli, B., He, H., Wichmann, P., Erb, T. J., Lindner, S. N., & Bar-Even, A. (2020). Awakening a latent carbon fixation cycle in Escherichia coli. *Nature Communications*, *11*(1). https://doi.org/10.1038/s41467-020-19564-5

SAUMWEBER, H., BINDER, R., & BISSWANGER, H. (1981). Pyruvate Dehydrogenase Component of the Pyruvate Dehydrogenase Complex from Escherichia coli K12. Purification and Characterization. *European Journal of Biochemistry*, *114*(2), 407–411. https://doi.org/10.1111/j.1432-1033.1981.tb05161.x

Schada von Borzyskowski, L., Severi, F., Krüger, K., Hermann, L., Gilardet, A., Sippel, F., Pommerenke, B., Claus, P., Cortina, N. S., Glatter, T., Zauner, S., Zarzycki, J., Fuchs, B. M., Bremer, E., Maier, U. G., Amann, R. I., & Erb, T. J. (2019). Marine Proteobacteria metabolize glycolate via the β-hydroxyaspartate cycle. *Nature*, *575*(7783), 500–504. https://doi.org/10.1038/s41586-019-1748-4

Schirch, V., Hopkins, S., Villar, E., & Angelaccio, S. (1985). Serine hydroxymethyltransferase from Escherichia coli: Purification and properties. *Journal of Bacteriology*, *163*(1), 1–7. https://doi.org/10.1128/jb.163.1.1-7.1985

Schneider, K., & Schlegel, H. G. (1976). Purification and properties of soluble hydrogenase from Alcaligenes eutrophus H 16. *BBA - Enzymology*, *452*(1), 66–80. https://doi.org/10.1016/0005-2744(76)90058-9

Schnorpfeil, M., Janausch, I. G., Biel, S., Kröger, A., & Unden, G. (2001). Generation of a proton potential by succinate dehydrogenase of *Bacillus subtilis* functioning as a fumarate reductase. *European Journal of Biochemistry*, *268*(10), 3069–3074. https://doi.org/10.1046/j.1432-1327.2001.02202.x

Schwander, T., McLean, R., Zarzycki, J., & Erb, T. J. (2018). Structural basis for substrate specificity of methylsuccinyl-CoA dehydrogenase, an unusual member of the acyl-CoA dehydrogenase family. *Journal of Biological Chemistry*, *293*(5), 1702–1712. https://doi.org/10.1074/jbc.RA117.000764

Shimakata, T., Fujita, Y., & Kusaka, T. (1979). Purification and characterization of 3-hydroxyacyl-coa dehydrogenase of Mycobacterium smegmatis. *Journal of Biochemistry*, *86*(5), 1191–1198. https://doi.org/10.1093/oxfordjournals.jbchem.a132634

Sprenger, G. A., Schorken, U., Sprenger, G., & Sahm, H. (1995). Transketolase a of Escherichia coli K12. Purification and Properties of the Enzyme from Recombinant Strains. *European Journal of Biochemistry*, *230*(2), 525–532. https://doi.org/10.1111/j.1432-1033.1995.0525h.x

Stoffel, G. M. M., Saez, D. A., DeMirci, H., Vögeli, B., Rao, Y., Zarzycki, J., Yoshikuni, Y., Wakatsuki, S., Vöhringer-Martinez, E., & Erb, T. J. (2019). Four amino acids define the CO2 binding pocket of enoyl-CoA carboxylases/reductases. *Proceedings of the National Academy of Sciences of the United States of America*, *116*(28), 13964–13969. https://doi.org/10.1073/pnas.1901471116

Tate, S., & Dalton, H. (1999). A low-molecular-mass protein from Methylococcus capsulatus (Bath) is responsible for the regulation of formaldehyde dehydrogenase activity in vitro. *Microbiology*, *145*(1), 159–167. https://doi.org/10.1099/13500872-145-1-159

Tsukamoto, Y., Fukushima, Y., Hara, S., & Hisabori, T. (2013). Redox control of the activity of phosphoglycerate kinase in synechocystis sp. PCC6803. *Plant and Cell Physiology*, *54*(4), 484–491. https://doi.org/10.1093/pcp/pct002

Ueda, Y., Yumoto, N., Tokushige, M., Fukui, K., & Ohya-nishiguchi, H. (1991). Purification and characterization of two types of fumarase from Escherichia coli. *Journal of Biochemistry*, *109*(5),

728–733. https://doi.org/10.1093/oxfordjournals.jbchem.a123448

Wadano, A., Nishikawa, K., Hirahashi, T., Satoh, R., & Iwaki, T. (1998). Reaction mechanism of phosphoribulokinase from a cyanobacterium, Synechococcus PCC7942. *Photosynthesis Research*, *56*(1), 27–33. https://doi.org/10.1023/A:1005979801741

Wang, Q., Ou, M. S., Kim, Y., Ingram, L. O., & Shanmugam, K. T. (2010). Metabolic flux control at the pyruvate node in an anaerobic escherichia coli strain with an active pyruvate dehydrogenase. *Applied and Environmental Microbiology*, *76*(7), 2107–2114. https://doi.org/10.1128/AEM.02545-09

Williams, P., Coates, L., Mohammed, F., Gill, R., Erskine, P., Bourgeois, D., Wood, S. P., Anthony, C., & Cooper, J. B. (2006). The 1.6 Å X-ray structure of the unusual c-type cytochrome, cytochrome cL, from the methylotrophic bacterium Methylobacterium extorquens. *Journal of Molecular Biology*, *357*(1), 151–162. https://doi.org/10.1016/j.jmb.2005.12.055

Wood, H. G., Jacobson, B., Gerwin, B. I., & Northrop, D. B. (1969). [36] Oxaloacetate transcarboxylase from Propionibacterium. *Methods in Enzymology*, *13*(C), 215–230. https://doi.org/10.1016/0076-6879(69)13041-4

Wu, T. Y., Chen, C. T., Liu, J. T. J., Bogorad, I. W., Damoiseaux, R., & Liao, J. C. (2016). Characterization and evolution of an activator-independent methanol dehydrogenase from Cupriavidus necator N-1. *Applied Microbiology and Biotechnology*, *100*(11), 4969–4983. https://doi.org/10.1007/s00253-016-7320-3

Yoshida, Y., Sato, M., Kezuka, Y., Hasegawa, Y., Nagano, K., Takebe, J., & Yoshimura, F. (2016). Acyl-CoA reductase PGN-0723 utilizes succinyl-CoA to generate succinate semialdehyde in a butyrate-producing pathway of Porphyromonas gingivalis. *Archives of Biochemistry and Biophysics*, *596*, 138–148. https://doi.org/10.1016/j.abb.2016.03.014

Yu, X., Niks, D., Mulchandani, A., & Hille, R. (2017). Efficient reduction of CO2 by the molybdenum-containing formate dehydrogenase from Cupriavidus necator (Ralstonia eutropha). *Journal of Biological Chemistry*, *292*(41), 16872–16879. https://doi.org/10.1074/jbc.M117.785576

Zadvornyy, O. A., Boyd, E. S., Posewitz, M. C., Zorin, N. A., & Peters, J. W. (2015). Biochemical and structural characterization of enolase from Chloroflexus aurantiacus: Evidence for a thermophilic origin. *Frontiers in Bioengineering and Biotechnology*, *3*(JUN), 74. https://doi.org/10.3389/fbioe.2015.00074

Zarzycki, J., Brecht, V., Müller, M., & Fuchs, G. (2009). Identifying the missing steps of the autotrophic 3-hydroxypropionate CO2 fixation cycle in Chloroflexus aurantiacus. *Proceedings of the National Academy of Sciences of the United States of America*, *106*(50), 21317–21322. https://doi.org/10.1073/pnas.0908356106

Zhang, R. G., Andersson, C. E., Savchenko, A., Skarina, T., Evdokimova, E., Beasley, S., Arrowsmith, C. H., Edwards, A. M., Joachimiak, A., & Mowbray, S. L. (2003). Structure of Escherichia coli ribose-5-phosphate isomerase: A ubiquitous enzyme of the pentose phosphate pathway and the Calvin cycle. *Structure*, *11*(1), 31–42. https://doi.org/10.1016/S0969-2126(02)00933-4

Ziegler, I. (1974). Malate dehydrogenase in zea may: Properties and inhibition by sulfite. *BBA - Enzymology*, *364*(1), 28–37. https://doi.org/10.1016/0005-2744(74)90129-6
